# Detecting Narcissistic Personality Disorder Traits on Forums: Proof-of-Concept Study

**DOI:** 10.2196/75799

**Published:** 2026-07-29

**Authors:** Ahmet Emre Aladağ, Arzucan Özgür, Naz Berfu Akbaş, Oguzhan Zahmacioglu, Haluk O Bingol

**Affiliations:** 1 Department of Computer Engineering Boğaziçi University İstanbul Turkey; 2 Department of Psychiatry Medical School Yeditepe University İstanbul Turkey; 3 Department of Child and Adolescent Psychiatry Medical School Yeditepe University İstanbul Turkey; 4 Faculty of Computer and Information Sciences Yeditepe University İstanbul Turkey

**Keywords:** narcissistic personality disorder, narcissism, pathological grandiosity, machine learning, text mining, artificial intelligence, natural language processing, transformer embeddings

## Abstract

**Background:**

Identifying traits of narcissistic personality disorder (NPD) is clinically challenging, yet early detection can significantly improve outcomes. Online forums have become a major source of self-expression, offering new opportunities to understand mental health. However, analyzing this complex language requires new tools.

**Objective:**

This study aims to determine whether a machine learning model could be trained to reliably detect language patterns associated with NPD traits in Reddit posts. Specifically, we sought to identify both authors who exhibit these traits and posts discussing individuals with these traits.

**Methods:**

We analyzed 75,985 posts from 4 Reddit communities (r/Narcissism, r/DeepThoughts, r/Showerthoughts, and r/ImposterSyndrome). A subset of 966 posts was annotated by 2 psychiatrists to create a reliable dataset. Using a range of machine learning techniques, from traditional text analysis to modern transformer-based embedding models, we trained the system to distinguish posts containing NPD-trait markers from general reflective posts. This secondary analysis of deidentified public Reddit data was exempted by the Boğaziçi University FMINAREK (Fen Bilimleri ve Mühendislik Alanları İnsan Araştırmaları Etik Kurulu) Institutional Review Board (exemption/application number 2025-13).

**Results:**

Our models demonstrated high accuracy. The modern embedding-based models were particularly effective, achieving a mean F1-score of 0.90, indicating a strong balance between recall and precision by correctly identifying posts with NPD-trait markers while minimizing false positives. The models remained effective even when common keywords such as “narcissism” were removed, with a mean relative F1-score decrease of 3.6% for the frequency-based baseline classifier and less than 2% (1.9% and 0.8%) for the embedding models.

**Conclusions:**

This study demonstrates that automated analysis of online posts is a promising approach for understanding and identifying NPD traits. Although this technology has potential for future clinical applications, it is currently a research tool and should be used only with strict ethical oversight, not for public-facing diagnosis.

## Introduction

### Background

Online platforms have become the de facto space for self-expression, discussion, and sharing. Along with the opinions we share, we leave digital traces that reflect our personality traits [1], emotional expressions [2], and even personality disorder traits [3].

Pioneering work by De Choudhury et al [4] showed that population-level shifts in social media language reliably anticipate depressive symptom trajectories, demonstrating that unobtrusive monitoring of mental health signals is both technically feasible and clinically meaningful. Extending this premise, Kosinski et al [1] showed that Facebook “Likes” patterns predict Big Five personality scores with near-clinical accuracy, confirming that everyday digital behavior encodes stable trait information, not just transient mood.

Digital health research has shown that once linguistic or affective markers are formalized, they can drive emotion-aware conversational agents and, ultimately, clinical decision-making pipelines [2,4]. We adopt this approach by translating markers of narcissistic grandiosity into embedding-based classifiers.

Building on this foundation, we leverage transformer-based natural language processing (NLP) to detect linguistic markers of narcissistic personality disorder (NPD) traits in long-form Reddit posts. By examining how individuals with elevated NPD signals talk about themselves and others, this work aims to establish the feasibility of automated NPD-trait detection as a foundation for future clinical applications in online mental health contexts.

### Understanding NPD

NPD is a psychological disorder characterized by grandiosity, a need for admiration, and a lack of empathy. Individuals with NPD often have a distorted self-image that differs markedly from reality. They have an inflated sense of self-worth and seek preferential attention and validation from those around them. NPD can significantly affect interpersonal relationships and broader social functioning [5,6].

### Diagnostic Criteria for NPD

The Diagnostic and Statistical Manual of Mental Disorders, Fifth Edition (DSM-5) [7] defines the diagnostic criteria for NPD, including grandiose self-importance; preoccupation with fantasies of unlimited success, power, beauty, or ideal love; and other characteristic features. Although the DSM-5 defines NPD as a single disorder, some research suggests that it comprises distinct subtypes, including grandiose, vulnerable, and exhibitionist [8-10]. In this study, we focused on the grandiose subtype, which aligns with the DSM-5 description.

### Challenges in Diagnosing NPD and Treating NPD

The lifetime prevalence of NPD in the United States is estimated to be 6.2% [11]. However, diagnosis and treatment remain challenging because of the disorder’s complex presentation. Recent text-mining studies of borderline personality disorder and bipolar disorder have shown that early algorithmic flagging of complex conditions can shorten diagnostic delays, underscoring the potential value of a similar detector for NPD [12]. Individuals with NPD frequently present with comorbid conditions, most commonly borderline personality disorder, depression, and anxiety, which further complicate accurate diagnosis [13].

Furthermore, denial of symptoms, withholding of information, and distortion of one’s personal narrative may further increase diagnostic complexity [14]. Symptom overlap with other disorders adds to this complexity [15].

Early treatment dropout is also common among individuals with NPD, interrupting both diagnostic clarification and therapeutic progress [16,17]. Taken together, these challenges indicate that identifying NPD requires a detailed, multimodal approach [18]. Even after diagnosis, resistance to change is frequently observed, necessitating specialized therapeutic strategies [19].

### Personal and Societal Impact of NPD

Individuals with NPD frequently display impaired empathy and exploitative interpersonal behaviors, which clinical studies have shown to increase tension and emotional distress in close relationships [5,6]. Roark [20] further documented how manipulative tactics and a persistent need for admiration can erode trust, leading to cycles of psychological harm within families and workplaces. Moreover, at the societal level, NPD traits are associated with increased organizational cynicism and reduced collaboration, contributing to broader public health and economic burdens [19]. These patterns motivate computational research on detecting NPD-related markers in online discourse, with the long-term goal of supporting early identification and targeted mental health outreach.

### Importance of Early Diagnosis and Tailored Therapeutic Approaches

Recent meta-analyses have shown that early intervention for personality and mood disorders measurably improves long-term outcomes, with digital screening tools reducing symptom severity by up to 30% when deployed during the prodromal stage [21]. Christensen et al [22] further demonstrated that eHealth platforms improve treatment adherence and engagement among individuals with anxiety and depression, confirming the value of scalable, technology-mediated outreach.

Building on these insights, we demonstrate that NLP methods applied to online forum language can identify linguistic markers associated with narcissistic patterns, providing a necessary technical foundation for future integration into clinical screening workflows [23].

### Aims, Objectives, and Methodological Approach

Building on Pennebaker et al’s [24] demonstration that lexical markers reveal psychological states and Reece and Danforth’s [25] success in predicting depression from Instagram embeddings, our study aims to develop and rigorously evaluate embedding-based classifiers for detecting narcissistic grandiosity in Reddit discourse. We compare traditional frequency- and sentiment-based features with transformer embeddings using an 80:20 train-test split and stratified 5-fold cross-validation, optimizing logistic regression (LR), support vector machine (SVM), random forest (RF), and Extreme Gradient Boosting (XGB) models on our manually annotated corpus. This proof-of-concept study not only benchmarks diverse feature representations and classification models but also establishes a replicable methodology and baseline for future research on automated NPD-trait detection in digital mental health contexts.

### Potential Applications of the Predictive System

If validated in clinical settings, embedding-based detection could be integrated into digital health platforms to assist clinicians in identifying individuals who may benefit from further assessment [22]. At the population level, such methods could support epidemiological research on personality disorder–related discourse and, with appropriate calibration, inform resource allocation decisions [21]. Content moderation represents another potential application, provided that fairness, false-positive rates (FPRs), and transparency are carefully evaluated [26,27]. Finally, researchers could leverage this framework for longitudinal and cross-cultural studies of narcissistic expression across digital communities, advancing both theory and practice in clinical personality science.

### Prior Work

Computational psycholinguistics has evolved rapidly. Pennebaker et al’s [24] linguistic inquiry and word count (LIWC) analyses first showed that lexical markers reveal emotional and personality signals, and De Choudhury et al [4] leveraged similar methods for depression detection on Twitter. Embedding-based approaches soon followed, with Reece and Danforth’s [25] demonstrating that vector representations of Instagram images and captions can predict depression [27]. Building on our previous proof-of-concept study of suicidal ideation detection in Reddit posts [28], we extend the same pipeline to the understudied domain of narcissistic grandiosity using text-embedding models.

Lexical models of personality suggest that fundamental personality traits are naturally reflected in the language people use. Accordingly, written text should contain strong signals of these underlying traits [29]. Sumner et al [30] and Preoțiuc-Pietro et al [31] applied machine learning to Twitter text to infer dark triad traits, including narcissism, achieving *F*_1_-scores of approximately 0.70. These advances demonstrate that both surface-level features and deep embeddings capture stable trait information. However, few studies have examined pathological grandiosity in longer, self-reflective forum posts. Our study addresses this gap by systematically comparing frequency-based and embedding-based classifiers for detecting NPD markers in Reddit narratives.

### Personality Trait Prediction Using Machine Learning

Across blogs, Facebook, and Reddit, researchers have shown that stylistic and semantic cues, such as pronoun use, emotion words, and discourse coherence, consistently reflect Big Five personality traits [32-36]. This body of work suggests that embedding models, which capture contextual nuances beyond n-gram counts, can robustly infer stable dispositional attributes from text. We therefore compare transformer embeddings with traditional term frequency-inverse document frequency (TF-IDF) and sentiment features to evaluate their relative performance in detecting pathological grandiosity.

### Personality Disorder Prediction Using Machine Learning

Previous research on clinically related conditions supports the feasibility of algorithmic detection. Twitter-based models have achieved moderate *F*_1_-scores (approximately 0.70) for dark triad traits, including narcissism [30,31], and LIWC-plus-regression approaches have correlated language use with narcissism scores in personal essays [37]. More recently, Bidirectional Encoder Representations from Transformers (BERT)–based classifiers have achieved approximately 75% accuracy across multiple disorders [38]. However, these studies relied on short texts or clinical assessment instruments rather than the rich, long-form narratives characteristic of Reddit. By contrast, our experiments leverage embeddings derived from long-form Reddit posts, hypothesizing that markers of grandiosity, which are subtler and more context dependent, are captured more reliably through deeper contextual representations.

### Ethical Context of Using Social-Media Data

Alongside these advances, concerns about privacy and the potential for misuse have also emerged. Binns et al [26] emphasized the need for transparency, fairness, and accountability in systems that predict personality traits. It is also important to acknowledge the risk that such predictive systems could be deployed in nonclinical settings, such as targeted advertising, workplace surveillance, or malicious profiling without individuals’ consent. As highlighted by Horvitz and Mulligan [27], a responsible and collaborative approach is essential to minimize the risks of stigmatization, discrimination, and unjust treatment. Although this study aims to provide a foundation for ethical applications, such tools should be deployed only under strict ethical guidelines and in collaboration with qualified mental health professionals.

Although Reddit posts are publicly accessible, contributors did not explicitly consent to their use in research and may not expect their content to inform mental health algorithms. This raises questions about users’ privacy expectations, data ownership, and the need for deidentification beyond simple username removal [39-41]. In this study, we obtained an institutional review board (IRB) exemption for the secondary analysis of publicly available data (see the “Methods” section) and applied best practices for anonymization and aggregate reporting throughout the study.

## Methods

### Data Collection

Reddit is a popular online discussion platform organized into topic-specific communities known as subreddits. Each subreddit focuses on a particular subject related to its name. Users can create posts consisting of a title and body text (referred to as selftext) within a subreddit. They can also upvote or downvote posts and leave comments. Most users participate under anonymous or pseudonymous usernames [42].

### Data Extraction and Temporal Coverage

We queried the Google BigQuery Reddit archive [42] using GoogleSQL to retrieve the title and body (selftext) of every post from r/Narcissism and the selected control subreddits between January 2008 and September 2019. At the time of data extraction, the archive provided full access to all subreddit tables. However, access to these data was revoked shortly thereafter, precluding the inclusion of posts from 2020 onward. We acknowledge that language use and community norms may have evolved since 2019, which could limit the applicability of our model to more recent online discourse.

### Subreddit Selection and Post Filtering

We selected r/Narcissism as our target community because it was the largest dedicated forum for discussions of pathological grandiosity, encompassing self-reports, third-party observations, and diagnostic queries. By contrast, related subreddits (eg, r/NPD and r/LifeAfterNarcissism) lacked sufficient post volume or topical breadth for robust model training at the time of data collection. To ensure linguistic consistency, we restricted our analysis to original post titles and bodies (selftext), excluding comments, which often contain reactive or more heterogeneous language.

The likelihood of narcissistic manifestations in general-purpose communities is low, given the population prevalence of NPD. We selected 3 subreddits as control communities: r/DeepThoughts, where users share philosophical reflections; r/Showerthoughts, where users post spontaneous realizations and observations; and r/ImposterSyndrome, where users discuss self-doubt and fears of being exposed as fraudulent. Collectively, these communities represent psychological themes that contrast with narcissistic grandiosity.

We extracted posts from these subreddits and excluded those with empty or deleted content or with body text containing fewer than 100 characters to ensure sufficient context for embedding-based feature extraction and reliable human annotation.

### Ethical Considerations

#### Ethics Review and Approval

This study used publicly available, deidentified Reddit posts and was determined to be exempt from full IRB review at Boğaziçi University (exemption/application number 2025-13).

#### Informed Consent

As this study involved the secondary analysis of publicly available data, the IRB waived the requirement for informed consent.

#### Privacy and Confidentiality

All posts were analyzed in aggregate, and no usernames or other identifying information was retained. The data were stored on secure computers. To prevent search-based reidentification, we paraphrased all illustrative post excerpts in the main manuscript and Multimedia Appendix 1 while preserving the linguistic features that each excerpt was selected to illustrate. Local Interpretable Model-Agnostic Explanations (LIME) attributions and all quantitative analyses were generated using the original, unparaphrased text.

#### Compensation

No compensation was provided because all data were obtained from publicly available sources.

#### Use of Identifiable Images

No images of individual users were included.

### Data Labeling

A total of 9 labels (L1-L9; Table 1) were defined a priori through an iterative pilot process led by our computational science team (AEA and HOB), allowing multilabel annotation. Drawing on the DSM-5 diagnostic criteria for NPD [7], we first coded 50 randomly sampled posts from the r/Narcissism subreddit to develop an initial label glossary. This preliminary schema was then reviewed and approved by 2 psychiatrists: NBA (an adult psychiatrist with clinical expertise in bipolar disorder, obsessive-compulsive disorder, depression, and anxiety) and OZ (a child psychiatrist), both of whom routinely encounter NPD in clinical practice. The 4 authors then collaboratively refined the label definitions and decision rules. A “Notes” field was included during annotation to flag posts that might require additional labels, although no new labels were ultimately identified.

**Table 1:** Labels and their corresponding descriptions.

Label	Description
L1	Normal talk (NPD^a^-trait-negative).
L2	The post contains markers of pathological grandiosity
L3	The author is asking whether the mentioned person exhibits NPD-consistent traits.
L4	The author is talking about a person displaying pathological grandiosity traits.
L5	The author is discussing NPD/pathological grandiosity concepts.
L6	The author may exhibit underlying pathological grandiosity markers, though none are explicitly present in this post.
L7	The author expresses markers of pathological grandiosity in this post.
L8	The author claims to exhibit NPD-consistent traits.
L9	The author is clinically diagnosed with NPD.

^a^NPD: narcissistic personality disorder.

Following finalization of the annotation schema, NBA and OZ completed a structured calibration session facilitated by AEA and HOB that included the following:

- Review of the DSM-5 trait definitions alongside the study-specific label glossary.
- Discussion of ambiguous or borderline cases.
- Joint review and annotation of a curated set of representative posts to align interpretation and coding decisions.

During the initial annotation phase, NBA and OZ each independently annotated 483 distinct posts from the r/Narcissism subreddit (966 annotations in total). To comprehensively assess annotation reliability, we adopted a dual approach.

### Dual Approach to Assess Annotation Reliability

#### Control-Subreddit Reliability

We randomly selected 30 posts from each of 3 control subreddits (r/Showerthoughts, r/DeepThoughts, and r/ImposterSyndrome), with all identifying metadata removed. Both annotators independently coded these 90 blinded posts.

#### Within-r/Narcissism Reliability

For the r/Narcissism subreddit, we assessed both intrarater reliability and interrater agreement. Each annotator independently completed 30 blinded annotations: 15 reannotations of posts they had previously labeled to assess intrarater reliability and 15 annotations of posts originally labeled by the other annotator to assess interrater agreement.

Labels L7 (expresses markers) and L8 (claims traits) were not assigned solely based on users’ self-reports. Instead, 2 experienced psychiatrists (with 23 and 20 years of clinical practice) evaluated each post for clinically meaningful markers consistent with the DSM-5 diagnostic criteria, including grandiosity, need for admiration, lack of empathy, entitlement, and interpersonal exploitation. This clinical validation ensured that our positive class (n=185) comprised posts containing psychiatrist-validated NPD markers across all categories, distinguishing our approach from studies that rely solely on self-reported data.

Using multiple, fine-grained labels allowed us to capture different aspects of the problem. Specifically, the labeling scheme makes it possible to:

- Predict whether an individual exhibiting NPD traits is involved in the post.
- Predict whether the author of the post appears to exhibit NPD traits.
- Predict whether the post contains markers consistent with NPD traits.
- Predict whether an individual with NPD traits is mentioned (eg, in the context of questions or complaints).
- Predict whether the post discusses the concept of NPD in general.
- Analyze the language used by individuals who exhibit NPD traits in their posts.

**Figure 1:**
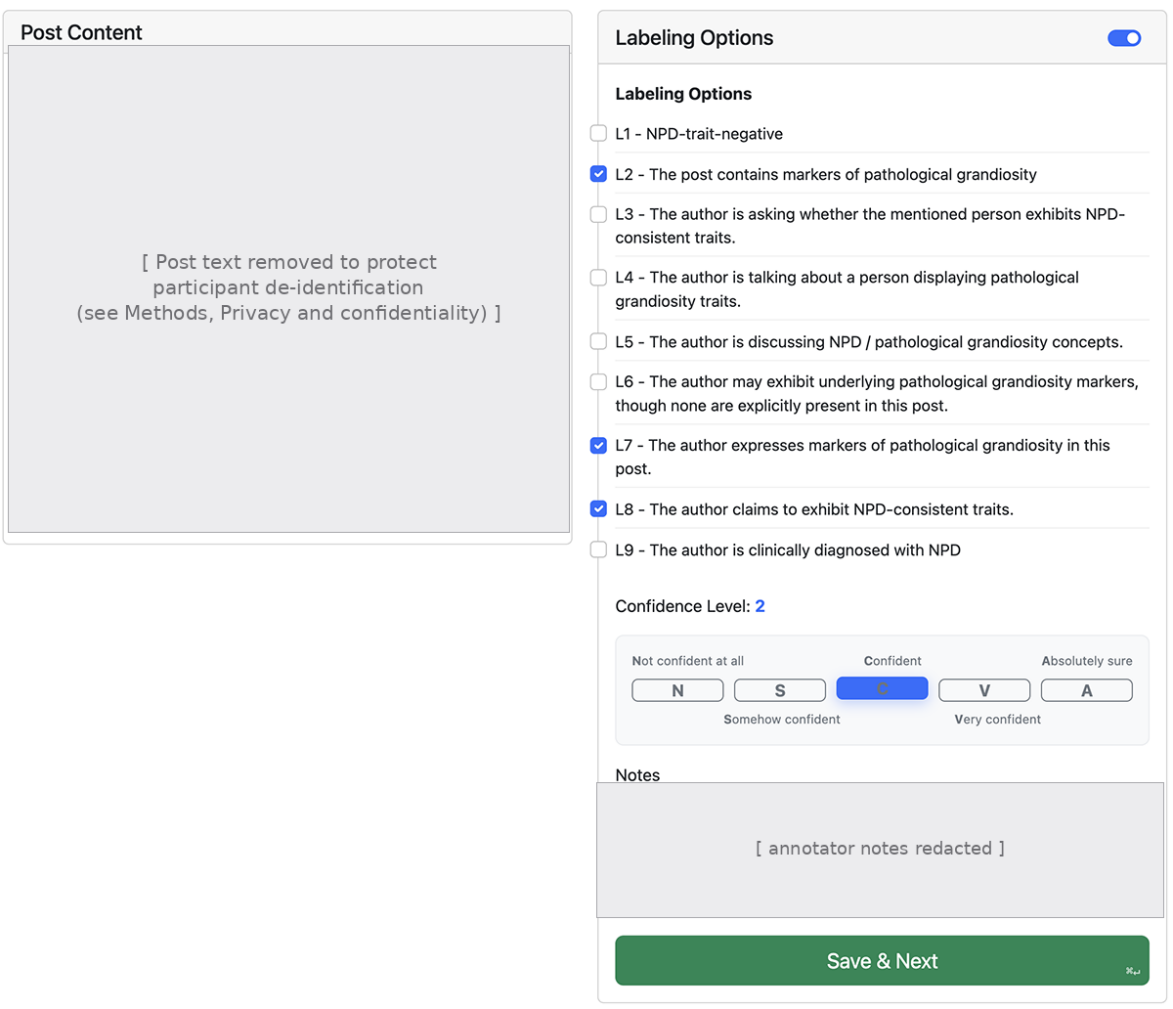
Labeling software [43]. The left panel displays the post, and the right panel contains the labeling tools and a notes field in which annotators record the rationale for their decisions. The example post content and annotator notes are redacted to protect privacy (see the "Privacy and Confidentiality" section).

We developed a custom software application [43] to support multilabel annotation. Figure 1 shows the annotation interface.

### Design of Experiments

#### Overview

We designed 3 experimental setups (A, B, and C). The experimental design for setup A is presented in Table 2.

For clarity, we define posts exhibiting linguistic markers of pathological grandiosity consistent with the DSM-5 diagnostic criteria for NPD as NPD-trait-positive and posts without such markers as trait-negative control posts (see the “Limitations” section for a discussion of construct interpretation). Depending on the experimental setup, the specific label assignments to the NPD-trait-positive and trait-negative classes varied.

**Table 2:** Experiment design for setup A and its objective^a^.

Experiment	Positive^b^	Negative
A1	NPD^c^-trait-positive	r/DeepThoughts
A2	NPD-trait-positive	r/ImposterSyndrome
A3	NPD-trait-positive	r/Showerthoughts
A4	NPD-trait-positive	NPD-trait-negative and r/Showerthoughts
A5	NPD-trait-positive	NPD-trait-negative

^a^The objective was to classify whether a post contains linguistic markers of pathological grandiosity (NPD-trait-positive vs trait-negative control posts).

^b^These positive-class posts were combined with negatively labeled posts in various data compositions in each experiment in setup A. See Table 3 for the dataset design for setup A.

^c^NPD: narcissistic personality disorder.

#### Setup A: Authors Exhibiting Pathological Grandiosity Markers

##### Binary Classification of NPD Traits

We first predicted whether the author of a post exhibited markers of pathological grandiosity (ie, NPD traits). Positive cases included posts assigned any of the following labels: (1) L7 (displays NPD traits); (2) L8 (claims to exhibit NPD traits); or (3) L9 (clinically diagnosed with NPD). We converted the annotations of posts from the r/Narcissism subreddit into binary class labels. Posts assigned any of the labels L7, L8, or L9 were classified as positive (ie, the author exhibited NPD traits). As posts could receive multiple labels, we applied explicit decision rules in setups A and B. A post was assigned to the positive class if it received at least one of the target labels (L7, L8, or L9), regardless of any co-occurring nontarget labels. For example, a post assigned both L4 and L7 was classified as positive. The decision rules for each setup are described in the corresponding sections, and the resulting label-to-class mappings are presented in Table 3 (setup A), Table 5 (setup B), and Table 7 (setup C).

**Table 3:** In setup A, the objective is to classify whether the author exhibits NPD^a^ traits.^b^

Experiment	L^c^7-9	L1-6	DT^d^	ST^e^	IS^f^
A1	+	N/A^g^	–	N/A	N/A
A2	+	N/A	N/A	N/A	–
A3	+	N/A	N/A	–	N/A
A4	+	–	N/A	–	N/A
A5	+	–	N/A	N/A	N/A

^a^NPD: narcissistic personality disorder.

^b^Positive indicators are marked with +.

^c^L: label.

^d^DT denotes posts from r/DeepThoughts.

^e^ST denotes posts from r/Showerthoughts.

^f^IS denotes posts from r/ImposterSyndrome.

^g^N/A: not applicable.

##### A1: r/Narcissism (+) Versus r/DeepThoughts

We used posts classified as NPD-trait-positive from the r/Narcissism subreddit and excluded the remaining posts from that subreddit. We then randomly sampled 185 posts from the r/DeepThoughts subreddit, where users share personal reflections, and used these as trait-negative control posts.

##### A2: r/Narcissism (+) Versus r/ImposterSyndrome

We used the NPD-trait-positive posts from the r/Narcissism subreddit and combined them with all available posts from the r/ImposterSyndrome subreddit, where users discuss their own experiences with impostor syndrome. These posts were treated as NPD-trait-negative. We then applied the SMOTE (Synthetic Minority Over-sampling Technique)-Tomek method to balance the data. This approach combines SMOTE [44] with Tomek Links [45].

##### A3: r/Narcissism (+) Versus r/Showerthoughts

We used the NPD-trait-positive posts from the r/Narcissism subreddit and randomly sampled an equal number of posts from the r/Showerthoughts subreddit, where users share spontaneous observations and reflections. These posts were treated as NPD-trait-negative.

##### A4: r/Narcissism (+) Versus (r/NPD-Trait-Negative and r/Showerthoughts)

We used the NPD-trait-positive posts from the r/Narcissism subreddit. We then created a control group consisting of an equal number of posts randomly sampled from the r/Showerthoughts subreddit and posts randomly sampled from the r/Narcissism subreddit that were labeled as negative (see Table 3). All of these posts were treated as NPD-trait-negative. We then randomly downsampled the combined trait-negative set by half to obtain equal numbers of posts in the positive and negative classes.

##### A5: r/Narcissism (+) Versus r/NPD-Trait-Negative

We designed a more challenging experimental setup using NPD-trait-positive posts from the r/Narcissism subreddit together with an equal number of posts from the same subreddit that were labeled as negative (see Table 3). This experiment was designed to determine whether a classifier could distinguish posts written by individuals exhibiting NPD traits from those written by individuals who do not. We expected lower predictive performance because of the higher level of noise in the dataset. Specifically, posts discussing narcissism in general or describing individuals with NPD traits other than the author were labeled as negative, although they may still contain NPD-related language, thereby introducing noise into the classifier. We therefore performed random undersampling rather than applying SMOTE-Tomek, as oversampling a noisy dataset could amplify the noise.

#### Setup B: Individuals Exhibiting Pathological Grandiosity Within Posts

We aimed to predict whether a post involves an individual (not necessarily the author) exhibiting NPD traits.

We replicated the experiments from setup A with a revised problem definition (Table 4), resulting in a different label-to-class mapping. Whereas setup A aimed to predict whether the author of a post (1) displayed NPD traits (L7), (2) claimed to exhibit NPD traits (L8), or (3) had a clinical diagnosis of NPD (L9), setup B aimed to predict whether the post involved an individual with NPD traits, regardless of whether that individual was the author or someone else mentioned in the post. Accordingly, we converted the annotations of posts from the r/Narcissism subreddit into binary class labels by including the additional labels L2, L4, and L6 in the positive class. The label-to-class mapping used in setup B is presented in Table 5.

**Table 4:** Experiment design for setup B and its objective^a^.

Experiment	Positive	Negative
B1	NPD^b^-trait-positive	r/DeepThoughts
B2	NPD-trait-positive	r/ImposterSyndrome
B3	NPD-trait-positive	r/Showerthoughts
B4	NPD-trait-positive	NPD-trait-negative and r/Showerthoughts
B5	NPD-trait-positive	NPD-trait-negative

^a^Detect involvement of individuals exhibiting pathological grandiosity traits within posts.

^b^NPD: narcissistic personality disorder.

**Table 5:** In setup B, the objective is to classify whether a post involves an individual exhibiting NPD^a^ traits.^b^

Experiment	L^c^2, L4, and L6-L9	L1, L3, and L5	DT^d^	ST^e^	IS^f^
B1	+	N/A^g^	–	N/A	N/A
B2	+	N/A	N/A	N/A	–
B3	+	N/A	N/A	–	N/A
B4	+	–	N/A	–	N/A
B5	+	–	N/A	N/A	N/A

^a^NPD: narcissistic personality disorder.

^b^Positive indicators are marked with +.

^c^L: label.

^d^DT denotes posts from r/DeepThoughts.

^e^ST denotes posts from r/Showerthoughts.

^f^IS denotes posts from r/ImposterSyndrome.

^g^N/A: not applicable.

#### Setup C: Fine-Grain Classification

##### Mutually Exclusive Class Assignment

**Figure 2:**
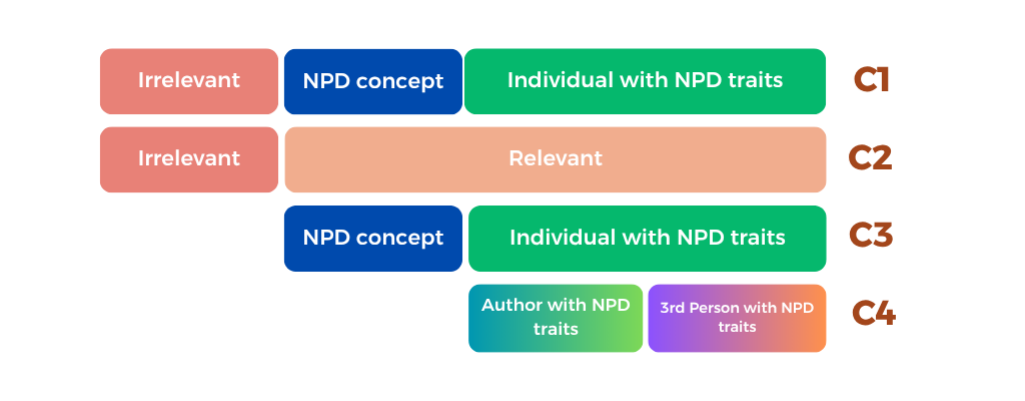
Layered view of the class structure for the fine-grained setup C experiments (C1-C4). Each row shows the class assignments for a given experiment. Block sizes and vertical alignment are schematic and do not indicate exact post counts or hierarchical nesting (see the setup C text for exact sample sizes). NPD: narcissistic personality disorder.

We used posts from the r/Narcissism subreddit to investigate increasingly fine-grained classification tasks (Tables 6 and 7; Figure 2). Unlike setups A and B, in which a post was assigned to the positive class if it contained any positive-class label regardless of co-occurring labels, setup C required mutually exclusive class assignments. Posts containing labels assigned to both the positive and negative classes for a given experiment were excluded to prevent label leakage. For example, in C4 (author vs third-person NPD markers), a post assigned both L4 (third person) and L7 (author expresses markers) was excluded. The same rule was applied in the multiclass experiment C1, in which posts spanning more than 1 of the 3 classes (irrelevant, NPD-related, or individual exhibiting NPD traits) were excluded. After these exclusions, the analyzed sample sizes were as follows: C1, n=935 (31 excluded; 183 irrelevant, 170 NPD-related, and 582 individual exhibiting NPD traits); C2, n=964 (2 excluded; 183 negative and 781 positive); C3, n=754 (29 excluded; 170 negative and 584 positive); and C4, n=602 (11 excluded; 410 negative and 192 positive).

**Table 6:** Experiment design for setup C: granular classification of NPD^a^ trait–related posts.

Experiment	Comparison	Objective
C1	Irrelevant vs NPD-related vs an individual exhibiting NPD traits	To determine whether a post involves an individual exhibiting NPD traits, is NPD-related, or is irrelevant.
C2	Irrelevant vs (NPD-related | an individual exhibiting NPD traits)	To determine whether the post is related to NPD traits, or individuals exhibiting NPD traits.
C3	NPD-related vs individuals exhibiting NPD traits	To determine whether a particular individual exhibiting NPD traits is involved in the post or the post is about narcissism.
C4	Author exhibiting NPD-traits vs a third person exhibiting NPD traits	To determine whether the mentioned individual exhibiting NPD traits is the author or another third person.

^a^NPD: narcissistic personality disorder.

**Table 7:** In setup C, the objective is to perform multiclass and layered binary classification.^a^

Experiment	L^b^1	L2	L3	L4	L5	L6	L7	L8	L9
C1^c^	a	b	c	c	b	c	c	c	c
C2^d^	d	e	e	e	e	e	e	e	e
C3^e^	–	f	g	g	f	g	g	g	g
C4^f^	–	–	h	h	–	i	i	i	i

^a^Cells marked with “–” indicate labels that were not used by the corresponding classifier.

^b^L: label.

^c^C1: 3-class classifier. a=irrelevant; b=narcissistic personality disorder related; and c=individual exhibiting narcissistic personality disorder traits.

^d^C2: a first-level binary classifier. d=irrelevant; and e=narcissistic personality disorder–related or an individual exhibiting narcissistic personality disorder traits.

^e^C3: second-level cascade binary classifier, which further classifies the ones classified as e in C2. f=narcissistic personality disorder related; and g=an individual exhibiting narcissistic personality disorder traits.

^f^C4: Third-level cascade binary classifier, which further classifies the ones classified as g in C3. h=a third person exhibiting narcissistic personality disorder traits; and i=the author exhibiting narcissistic personality disorder traits.

These samples are not nested, although Figure 2 presents C2-C4 as a conceptual cascade. Each experiment was trained and evaluated using its own ground-truth labels rather than the predictions from the preceding experiment. C3 excluded the 29 posts that were ambiguous with respect to the contrast between NPD-related content (L2 and L5) and posts involving individuals exhibiting NPD traits (L3, L4, and L6-L9). By contrast, C4 addressed a different classification task (author vs third person) and therefore reinstated those 29 posts, drawing from all 613 posts involving individuals exhibiting NPD traits while excluding only the 11 posts assigned to both classes (n=602 = 584 + 29 − 11). The individual-exhibiting class in C1 (n=582) was slightly smaller than that in C3 (n=584) because C1 additionally excluded the 2 posts that also carried the irrelevant label (L1).

Using posts from the r/Narcissism subreddit, we implemented a multiclass and cascade classification framework to investigate increasingly fine-grained classification tasks. The framework consisted of the following 4 steps:

- C1: a multiclass classification task that categorized each post as NPD related, an individual exhibiting NPD traits, or irrelevant.
- C2: a binary classification task using the same dataset to determine whether a post was NPD related or unrelated (normal discussion).
- C3: a binary classification task applied to the NPD-related posts to determine whether a post discussed the concept of NPD or a specific individual exhibiting NPD traits.
- C4: a binary classification task applied to posts mentioning specific individuals exhibiting NPD traits to determine whether the individual was the author or another person.

This stepwise approach first captures broad classification patterns (C1) and then incrementally refines the classification (C2-C4) to achieve greater specificity.

##### C1: Can We Classify This Post Into 3 Classes?

We aimed to classify posts into 1 of 3 categories: irrelevant (L1), NPD related (L2 and L5), or an individual exhibiting NPD traits (L3, L4, and L6-L9). We used a OneVsRestClassifier (one-vs-all) with LR for this multiclass classification task. This approach fits 1 classifier for each class, with each classifier trained to distinguish its target class from all remaining classes. As multiclass classification is inherently more complex than binary classification, lower predictive performance was expected.

##### C2: NPD Theme

As an alternative to experiment C1, we performed a series of binary classification tasks. In the first task (C2), we aimed to determine whether a post was related to the theme of NPD. Posts labeled normal talk (L1) were assigned to the negative class (unrelated), whereas all remaining posts were assigned to the positive class (NPD related).

##### C3: NPD-Related Versus Individuals Exhibiting NPD Traits

In this experiment, we included only posts classified as NPD related in C2 (ie, posts assigned labels L2-L9). We then categorized these posts into 1 of 2 classes: (1) NPD related (L2 and L5) or (2) individual exhibiting NPD traits (L3, L4, and L6-L9).

##### C4: Author Exhibiting NPD Traits Versus an Individual in the Post Exhibiting NPD Traits

We then aimed to distinguish whether the individual exhibiting NPD traits was the author of the post (L6-L9) or another person mentioned in the post (L3 and L4).

### Feature Engineering Summary

We used 2 main approaches for feature extraction: (1) frequency- and sentiment-based analysis (FSA) and (2) text-embedding models. The FSA approach involved standard NLP preprocessing and TF-IDF– and sentiment-based features, whereas the embedding approach used transformer-based models, including BERT [46], Large Language Model Meta AI 3 (LLaMA3) [47], MiniLM [48], Nomic [49], and OpenAI’s embedding API. Feature selection was performed using ANOVA [50] *F* values, and class imbalance was addressed through SMOTE-Tomek or random downsampling. For the FSA features, SelectKBest with f_classif was implemented within the scikit-learn pipeline fitted by GridSearchCV, ensuring that feature selection was performed independently within each cross-validation training fold without access to the corresponding validation fold or the held-out test set. For transformer embeddings, the feature-selection step was bypassed (passthrough), and no feature selection was performed. Classification models included LR, SVM, XGB, and RF, with 5-fold cross-validation used for hyperparameter tuning. Full methodological details are provided in Multimedia Appendix 2.

### Class Imbalance and Resampling Strategy

The prevalence of NPD traits in the general population is low (approximately 1%-6%) [11]. Consequently, when the r/Narcissism subreddit is compared with high-traffic control communities (eg, r/Showerthoughts, with approximately 72,000 posts), the raw corpus is inherently imbalanced. This reflects prospective surveillance settings in which the target class is expected to be rare. We therefore adopted a 2-step strategy: (1) setup A and B experiments, except A2/B2, were balanced through equal sampling or random undersampling during data preparation; and (2) experiments A2/B2 and setup C experiments (C1-C4) retained their natural class distributions and used SMOTE-Tomek to synthesize minority samples and remove Tomek links. For these imbalanced experiments, SMOTE-Tomek was applied within the training pipeline. For the prebalanced experiments, the resampling step was set to passthrough; neither SMOTE interpolation nor Tomek-link removal was performed.

We did not resample the held-out test folds; therefore, they retained their stratified class composition (50% positive in A1, A3-A5, B1, B3, B4, and B5; 74% in A2; and 89% in B2) rather than reflecting the 1%-6% population prevalence. Receiver operating characteristic-area under the curve (ROC-AUC) and recall are independent of prevalence as long as the class-conditional score distributions remain stable. By contrast, precision and *F*_1_-scores are prevalence dependent, and the values reported here correspond to the prevalence within the test sets. The “Limitations” section discusses the implications of this approach for deployment and the assumptions underlying its interpretation.

### Machine Learning Pipeline Overview

#### Feature Engineering and Classification Models

We compared 2 feature-engineering pipelines. The frequency and sentiment analysis (FSA) approach combined classical text features (TF-IDF n-grams and sentiment scores generated using TextBlob), extracted with scikit-learn and the Natural Language Toolkit. The second pipeline generated dense embeddings from transformer models, including all-MiniLM-L6-v2 via sentence-transformers v2.2.2 and OpenAI’s text-embedding-3-large. For each pipeline, we trained 4 classifiers: LR as an interpretable baseline, SVM for high-dimensional margin-based separation, RF for capturing nonlinear interactions, and XGB for enhanced predictive performance. These models were selected to represent both linear and ensemble approaches.

All models were evaluated using an 80/20 stratified train-test split (random_state=42), with hyperparameters optimized through 5-fold cross-validation within the 80% training set. We report *F*_1_-score (the harmonic mean of precision and recall), ROC-AUC, and accuracy on the held-out 20% test set. Binary predictions were generated using the default 0.5 decision threshold, and the test set was not used to optimize the operating point. For the multiclass experiment (C1), we report weighted *F*_1_-score (scikit-learn average=“weighted”), in which each class’s *F*_1_-score is weighted according to its support. To ensure full reproducibility, all stochastic steps, including data splitting, SMOTE-Tomek balancing, feature selection, and model training, used fixed random seeds. Environment details, exact code snippets, and package versions are provided in Multimedia Appendix 2.

Given the relatively small size of our dataset, we selected 5-fold cross-validation as a balance between reliable performance estimation and reasonable computational cost. Using 10-fold cross-validation would have reduced each validation fold to only 10% of the training data, potentially increasing variance in performance estimates. Moreover, our preliminary experiments showed negligible improvements in mean *F*_1_-score compared with 5-fold cross-validation. The remaining 20% of the data was held out entirely (not used during cross-validation) and evaluated only once after completion of all model tuning to provide an unbiased assessment on the test set.

#### Compliance With Machine Learning Reporting Guidelines

We adhered to the Guidelines for Developing and Reporting Machine Learning Predictive Models in Biomedical Research [51], documenting each stage of our workflow, from data sourcing and outcome definition to feature engineering, hyperparameter tuning, internal validation, and performance reporting. This ensured transparency and reproducibility (see Multimedia Appendix 3 for the completed checklist).

#### Data Collection and Cohort Definition

- Time window: We collected all publicly available Reddit posts from January 1, 2009, to August 31, 2019.
- Subreddits: We retrieved posts from r/Narcissism, r/ImposterSyndrome, r/Showerthoughts, and r/DeepThoughts.
- Language filter: We retained only English-language posts.
- Length filter: We excluded posts with fewer than 100 characters.

## Results

### Data Extraction Results

See Table 8 for the post count for each subreddit.

**Table 8:** Number of posts extracted from each subreddit.

Subreddit	Count, n
r/Narcissism	966
r/DeepThoughts	2216
r/Showerthoughts	72,740
r/ImposterSyndrome	63
Total	75,985

### Annotation Results

See Table 9 for the frequencies of each label and their overlaps. The rowSum column is calculated as 
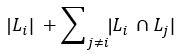
; therefore, it exceeds |*L_i_*| whenever any off-diagonal entry in the corresponding row is nonzero. This occurs because each multilabeled post is counted once for each additional label assigned.

Analysis of label co-occurrence revealed several notable patterns. The most frequent triple-label combination was the co-occurrence of L3, L7, and L8, which appeared in 4 posts. Other triple-label combinations included L3, L4, and L8 (2 posts), whereas the combinations {L2, L3, L4}, {L2, L4, L9}, and {L2, L7, L8} each appeared in 1 post.

For setup A, the positive class consisted of posts assigned labels L7, L8, or L9, comprising 185 posts. Inclusion-exclusion using the marginal totals and pairwise intersections from Table 9 confirms this: |L7 ∪ L8 ∪ L9| = |L7| + |L8| + |L9| − |L7∩L8| − |L7∩L9| − |L8∩L9| + |L7∩L8∩L9| = 53 + 123 + 17 − 7 − 0 − 1 + 0 = 185. The triple-intersection term is 0 because no post in the corpus was assigned all 3 labels (L7, L8, and L9). Each of these 185 posts contained either 1 or 2 of the 3 labels. See Multimedia Appendix 4 for the observed label overlaps.

**Table 9:** Co-occurrence matrix of annotated posts.^a^

Label	L^b^1	L2	L3	L4	L5	L6	L7	L8	L9	rowSum	diagonal	diagonalOff
L1	*185*	0	2	0	0	0	0	0	0	187	185	2
L2	0	*22*	2	6	1	0	1	3	1	36	22	14
L3	2	2	*145*	25	3	0	4	6	0	187	145	42
L4	0	6	25	*301*	11	0	0	6	1	350	301	49
L5	0	1	3	11	*178*	0	0	5	0	198	178	20
L6	0	0	0	0	0	*18*	0	0	0	18	18	0
L7	0	1	4	0	0	0	*53*	7	0	65	53	12
L8	0	3	6	6	5	0	7	*123*	1	151	123	28
L9	0	1	0	1	0	0	0	1	*17*	20	17	3
Sum	187	36	187	350	198	18	65	151	20	*1212*	*1042*	*170*

^a^Italicized diagonal entries represent marginal totals (|*L_i_*|), indicating the number of posts carrying label *L_i_* (possibly with other labels), rather than single-label counts. Off-diagonal entries (*i*,*j*) represent pairwise intersections (|*L_i_* ∩ *L_j_*|). See Table 1 for label definitions.

^b^L: label.

### Annotation Reliability Assessment

We conducted a comprehensive reliability assessment that included both interrater agreement (between annotators) and intrarater reliability (temporal stability). Full reliability metrics are provided in Multimedia Appendix 5. Reliability was assessed using 30 blinded r/Narcissism posts, with fair agreement on the 9-point scale (κ=0.39) and substantial agreement on binary classifications (setup A: κ=0.75; setup B: κ=0.79). Intrarater reliability showed fair agreement on the 9-point scale (κ=0.23) and substantial agreement on binary classifications (mean κ=0.74 for setup A and 0.66 for setup B), indicating good temporal stability. Cross-subreddit validation using 90 blinded posts (30 from each subreddit) confirmed minimal NPD trait presence in control communities, with annotators demonstrating high agreement (Gwet first-order agreement coefficient [AC1] is 0.93-1.00) regarding the expected low prevalence.

### Experimental Results

In this section, we present the performance of our different classification models. The primary objective was to determine which combination of text analysis approach (the “Embedding Model”) and algorithm (the “Classifier”) performed best in identifying posts containing NPD-trait markers. A successful model must achieve 2 goals: correctly identifying posts that contain NPD traits (measured by recall) and avoiding incorrect classification of posts without such traits (measured by precision). The *F*_1_-score provides a single metric that reflects the balance between these 2 objectives.

The results (see Table 10 and Textbox 1) demonstrate the feasibility of using machine learning to identify posts exhibiting NPD traits in online forums with high reliability. Across all experiments, embedding-based approaches consistently achieved high *F*_1_-scores, outperforming or matching the FSA approach. Among all models, the 2 OpenAI embedding models (text-embedding-ada-002 and text-embedding-3-large) showed the best average performance across experiments (essentially tied; see the “Principal Findings” section). In experiments A1-A3 and B1-B3, we achieved near-perfect *F*_1_-scores.

When evaluated on noisier datasets in A4-A5, we observed a slight decrease in performance; however, the best-performing embedding models still achieved *F*_1_-scores of approximately 0.89.

As shown in Table 10, the 2 OpenAI embedding models performed best on average (see the “Principal Findings” section). A companion interpretability analysis of the FSA + LR baseline (see Multimedia Appendix 1) found that, after keyword removal, the top predictive features shifted toward emotional, cognitive, and relational cues (eg, emotional reasoning phrases and relational conflict terms) rather than explicit diagnostic labels. LIME was applied for local explanations but was less informative than coefficient analysis because of a structural sparsity artifact in TF-IDF features. However, this finding may also partly reflect genuine reliance on topical information (see Section S7 in Multimedia Appendix 1; see also the “Limitations” section).

**Table 10:** Classification results.^a^

Experiments and embedding model			*F*_1_-score		ROC-AUC^b^		Accuracy		Precision		Recall		Classifier
**A1**													
	all-minilm	0.986		0.993		0.986		1.000		0.973		Logistic regression	
	llama3	0.986		1.000		0.986		1.000		0.973		Logistic regression	
	nomic-embed-text	0.986		0.995		0.986		1.000		0.973		Logistic regression	
**A2**													
	Fsa	1.000		1.000		1.000		1.000		1.000		Logistic regression	
	text-embedding-3-large	1.000		1.000		1.000		1.000		1.000		Random forest	
	text-embedding-ada-002	1.000		1.000		1.000		1.000		1.000		Random forest	
**A3**													
	all-minilm	0.986		0.999		0.986		1.000		0.973		Extreme Gradient Boosting	
	nomic-embed-text	0.986		0.997		0.986		1.000		0.973		Logistic regression	
	text-embedding-3-large	0.986		1.000		0.986		1.000		0.973		Logistic regression	
**A4**													
	text-embedding-ada-002	0.895		0.920		0.892		0.872		0.919		Support vector machine	
	all-minilm	0.889		0.951		0.892		0.914		0.865		Random forest	
	text-embedding-3-large	0.861		0.957		0.865		0.886		0.838		Extreme Gradient Boosting	
**A5**													
	text-embedding-ada-002	0.892		0.939		0.892		0.892		0.892		Logistic regression	
	text-embedding-3-large	0.853		0.948		0.865		0.935		0.784		Support vector machine	
	all-minilm	0.849		0.901		0.851		0.861		0.838		Random forest	
**B1**													
	text-embedding-3-large	1.000		1.000		1.000		1.000		1.000		Logistic regression	
	text-embedding-ada-002	0.995		1.000		0.995		0.990		1.000		Random forest	
	nomic-embed-text	0.981		0.999		0.980		0.971		0.990		Extreme Gradient Boosting	
**B2**													
	text-embedding-3-large	1.000		1.000		1.000		1.000		1.000		Support vector machine	
	text-embedding-ada-002	1.000		1.000		1.000		1.000		1.000		Support vector machine	
	nomic-embed-text	0.990		0.997		0.983		0.981		1.000		Support vector machine	
**B3**													
	nomic-embed-text	0.995		0.999		0.995		0.990		1.000		Random forest	
	text-embedding-3-large	0.995		0.999		0.995		0.990		1.000		Support vector machine	
	text-embedding-ada-002	0.995		0.998		0.995		0.990		1.000		Support vector machine	
**B4**													
	bert-base-uncased	0.860		0.897		0.858		0.848		0.873		Logistic regression	
	text-embedding-ada-002	0.850		0.913		0.848		0.838		0.863		Extreme Gradient Boosting	
	text-embedding-3-large	0.849		0.922		0.843		0.818		0.882		Extreme Gradient Boosting	
**B5**													
	nomic-embed-text	0.744		0.767		0.721		0.685		0.813		Random forest	
	bert-base-uncased	0.743		0.771		0.732		0.710		0.780		Random forest	
	text-embedding-3-large	0.735		0.791		0.716		0.686		0.791		Random forest	
**C1**													
	bert-base-uncased	0.685		0.809		0.684		0.688		0.684		OneVsRestClassifier	
	fsa	0.672		0.778		0.679		0.666		0.679		OneVsRestClassifier	
	text-embedding-ada-002	0.655		0.788		0.647		0.670		0.647		OneVsRestClassifier	
**C2**													
	text-embedding-ada-002	0.916		0.813		0.855		0.860		0.981		Random forest	
	all-minilm	0.906		0.766		0.834		0.837		0.987		Random forest	
	text-embedding-3-large	0.905		0.804		0.834		0.844		0.974		Random forest	
**C3**													
	text-embedding-ada-002	0.933		0.909		0.894		0.911		0.957		Extreme Gradient Boosting	
	nomic-embed-text	0.925		0.867		0.881		0.902		0.949		Extreme Gradient Boosting	
	all-minilm	0.913		0.853		0.854		0.852		0.983		Support vector machine	
**C4**													
	text-embedding-ada-002	0.875		0.934		0.917		0.854		0.897		Extreme Gradient Boosting	
	text-embedding-3-large	0.831		0.959		0.893		0.842		0.821		Random forest	
	nomic-embed-text	0.804		0.938		0.851		0.698		0.949		Logistic regression	

^a^The frequency- and sentiment-based analysis approach combines sentiment analysis with the term frequency-inverse document frequency method, whereas the remaining approaches use the specified text embedding models. The 3 best-performing embedding models for each experiment are presented. Complete results are provided in Sheet S2 in Multimedia Appendix 6. The *F*_1_-score refers to the binary *F*_1_-score for the binary experiments (A1-A5, B1-B5, and C2-C4); for the multiclass C1 experiment, weighted *F*_1_-score is reported (scikit-learn average=“weighted”).

^b^ROC-AUC: area under the receiver operating characteristic curve.

**Definition of variables in Table 10.**

- Embedding model: The text embedding method used to convert each Reddit post into numerical features before classification (eg, text-embedding-3-large, all-MiniLM, Large Language Model Meta AI 3 [LLaMA3]).

- Classifier: The machine learning algorithm applied to the extracted features (eg, logistic regression, random forest, support vector machine, Extreme Gradient Boosting).
- F1-score: The harmonic mean of precision and recall, providing a balance between false positives and false negatives.
- Area under the receiver operating characteristic curve: A performance metric used to measure the model’s ability to discriminate between classes across all classification thresholds.
- Accuracy: The proportion of all predictions (both positive and negative) that are correctly classified by the model.
- Precision: Also known as positive predictive value; the proportion of positively predicted instances that are true positives.
- Recall: Also known as sensitivity or true positive rate; the proportion of actual positive instances correctly identified by the model.

## Discussion

### Principal Findings

#### Language Patterns Associated With NPD-Trait Markers

Echoing De Choudhury et al’s [4] depression prediction work [4] and Kosinski et al’s [1] trait forecasting findings [1], our results indicate that online language contains strong signals of NPD-trait markers. Embedding-based models reliably distinguished psychiatrist-labeled NPD-trait posts from control posts across diverse experimental configurations (discussed below and in the “Limitations” section). The interpretability analysis of the FSA + LR baseline further showed that the classifier’s feature profile varied systematically across experimental designs.

After keyword removal, the FSA + LR model relied on features with plausible clinical relevance rather than explicit diagnostic labels. Reflective terms (eg, “aware”) carried positive coefficients for the NPD-positive class, consistent with self-referential reflective language in r/Narcissism (eg, a poster acknowledging awareness of these patterns in themselves). In the A5 no-keyword model, “aware” and “hurt” appeared among the positive predictors, consistent with reflective and distress-related language in the support community. As r/Narcissism is a self-selected community of users engaging with NPD-related content, these within-community word-frequency differences reflect discourse patterns in the support community itself. Whether they represent NPD-specific distress or broader emotional-distress vocabulary is addressed in the section “What Do These Automated Classifiers Actually Learn?”.

#### Task Complexity and Contextual Information

To assess the practical reliability of these models, we evaluated them under a range of conditions, from clean comparisons to more challenging, noisy scenarios.

We observed consistently high performance across metrics such as *F*_1_-score and accuracy, particularly in experiments A1-A3 and B1-B3. These experiments focused on detecting authors exhibiting NPD traits or posts involving individuals with NPD-consistent trait markers. The availability of contextual cues likely contributed to the superior performance observed in these experiments compared with the remaining experiments.

The performance decreases observed in experiments A4, A5, B4, and B5 were expected and can be attributed to the inclusion of noisy, negatively labeled data from the r/Narcissism subreddit. Many of these negatively labeled posts were related to pathological grandiosity markers or discussed individuals exhibiting NPD traits. As posts from both classes shared terminology related to narcissism, distinguishing between the classes became more challenging.

Experiment A5 still achieved relatively strong performance because author-specific contextual cues helped the model distinguish posts written by individuals exhibiting NPD traits. However, experiment B5 was more negatively affected because it lacked direct cues from the author. The model instead had to interpret indirect references, third-party mentions, and more complex contextual information.

This performance pattern also raises a construct validity concern specific to setup B. As the positive class includes 6 label types (L2, L4, L6, and L7-L9; see the “Methods” section, setup B), the training signal combines linguistically distinct populations, ranging from self-aggrandizing or trait-disclosing language to distress-oriented accounts of others’ behaviors. A classifier trained on such heterogeneous signals may learn a composite discourse pattern rather than specific psychological trait markers. The lower *F*_1_-score in B5 (0.744) compared with A5 (0.892) is consistent with this interpretation: when both classes share r/Narcissism-related vocabulary and the positive class contains heterogeneous psychological perspectives, classification becomes more challenging. Therefore, a positive classification in setup B indicates that a person exhibiting NPD traits is involved in the post (as the author or as the subject of discussion), rather than indicating that the author themselves exhibits specific trait markers.

Multiclass classification in experiment C1 aimed to differentiate among 3 related categories and therefore involved greater complexity than the binary classification tasks. As expected, C1 performed below the binary experiments. Nonetheless, it achieved a reasonable weighted *F*_1_-score of 0.685 (bert-base-uncased) for the 3-way classification task (irrelevant vs NPD-related vs an individual exhibiting NPD traits).

#### What Do These Automated Classifiers Actually Learn?

A crucial follow-up question is what these automated classifiers actually learn: do they simply identify keywords, or do they capture broader linguistic patterns?

We used identical hyperparameter ranges across models, limiting variation attributable to model tuning and allowing a more direct comparison of feature representations. Embedding models consistently outperformed the FSA approach, likely because of their richer representational capacity and ability to capture contextual linguistic patterns beyond surface-level features (see Table 10).

They demonstrated strong performance, with *F*_1_-scores ranging from 0.744 to 1.0. However, further validation across diverse datasets is required to confirm these findings. All embedding models performed similarly; the 2 OpenAI models showed the best overall performance, with mean *F*_1_-scores within 0.01 of each other, and text-embedding-ada-002 performing marginally better. It ranked first or tied for first in 9 of 14 experiments, whereas text-embedding-3-large ranked first or tied for first in 6 of 14 experiments. These counts overlap because the 2 models tied for first place in several experiments. The mean *F*_1_-scores across experiments were 0.907 (SD 0.101) for text-embedding-ada-002 and 0.897 (SD 0.106) for text-embedding-3-large. The complete results are provided in Sheet S2 in Multimedia Appendix 6.

We attribute the perfect precision or recall scores (1.0) achieved in a few experiments to evaluation on a small test set. Additionally, the clear separation of linguistic features between r/Narcissism and r/ImposterSyndrome enabled the models to achieve perfect *F*_1_-scores (1.0).

To assess whether the models captured linguistic patterns beyond explicit diagnostic labels, we retrained them after removing keyword families at 2 levels of aggressiveness using 2 complementary analyses. The first involved removing 4 keyword families (narcissism, npd, disorder, and personality) and evaluating the models using the same grid-search pipeline as in the main analysis across all feature models. The embedding models remained largely unaffected, with a mean decrease of only 0.37%, compared with 3.25% for the FSA models (Multimedia Appendix 6).

The second analysis involved more aggressive removal of 6 keyword families by additionally excluding the keyword families “self-absorbed” and “diagnose,” which are broader and not exclusively specific to NPD. This analysis was conducted separately using the FSA + LR interpretability pipeline. The FSA + LR pipeline showed a mean relative *F*_1_-score decrease of 3.6% across 13 configurations, with individual experiments ranging from a 15.0% decrease to an 8.4% increase. Two configurations showed slight improvements after keyword removal (B5: +8.4%; C3: +1.8%). These values represent the differences between the keyword-removed and original-text versions of the FSA + LR pipeline, not the changes in the best-model *F*_1_-scores reported in Table 10. The embedding models again showed minimal changes, with mean relative *F*_1_-score decreases of 1.9% for all-MiniLM-L6-v2 and 0.8% for text-embedding-3-large (per-experiment results are provided in Section S8 in Multimedia Appendix 1). The FSA and embedding results are not directly comparable because the FSA decrease is based on a single classifier, whereas the embedding decreases are averaged across 3 classifiers (LR, SVM, and RF). Therefore, this comparison should be considered descriptive rather than a like-for-like evaluation.

No experiment showed a substantial relative *F*_1_-score decline after keyword removal, indicating that the models retained predictive signal beyond explicit diagnostic labels. Per-experiment significance tests were underpowered because of the small test-set sizes (n=50-204); therefore, we based this robustness claim on the consistent pattern observed across experiments rather than on any individual test (see the “Statistical Power for Per-Experiment Comparisons” section). LR coefficient analysis of the keyword-excluded models showed that, in the absence of diagnostic terms, the highest-ranked features shifted toward broader emotional, cognitive, and relational cues (see Sections S4-S6 in Multimedia Appendix 1). However, LIME-based local explanations were less informative than expected because of a structural sparsity artifact in the TF-IDF features (see Section S7 in Multimedia Appendix 1).

Coefficient analysis of the FSA + LR pipeline provides additional evidence that the model captures signal beyond diagnostic keywords. In the no-keyword A2 model, the top positive coefficients included mental, behavior, important, issues, and society—terms reflecting general psychological and social vocabulary rather than diagnostic labels (although A2’s near-perfect classification may partly reflect topical separability between r/Narcissism and r/ImposterSyndrome). In the within-subreddit A5 model, where both classes shared community vocabulary, the top coefficients after keyword removal included think, low, second, aware, and look, suggesting reliance on common cognitive and observational terms. Across the deep-tier experiments, keyword removal shifted the composition of the top 20 coefficients from 5%-40% diagnostic terms to 0%, with broader emotional, cognitive, relational, and behavioral cues filling the vacated ranks. This shift is partly mechanical because removing keywords from a fixed top-20 list necessarily promotes the next-highest-ranked features. Thus, the analysis shows what the model relies on when keywords are unavailable, rather than demonstrating that nonkeyword features were always the primary drivers. A residual surrogate term (covert, shorthand for covert narcissist) remained highly ranked in the no-keyword models. A third ablation tier that additionally removed this term along with 2 other surrogate terms (tendencies and traits) produced negligible further changes in *F*_1_-score (see the “Statistical Power for Per-Experiment Comparisons” section). The keyword removal list did not exhaust NPD community jargon (eg, supply, love bombing, and gray rock). However, these terms did not appear among the highest-ranked coefficients in any deep-tier experiment (see Sections S4.4, S5.3, and S6.5 in Multimedia Appendix 1), indicating that they did not anchor the FSA + LR interpretability analysis, although other unlisted community terms may have contributed residual signal (see Section S9 in Multimedia Appendix 1).

These coefficient profiles reveal a design-dependent pattern in what the classifier relies on. In the cross-subreddit experiments (A1 and A2), where 8 of the top 20 positive coefficients in the original model were diagnostic keywords, keyword removal eliminated these terms and shifted the profile toward a mix of behavioral, cognitive, and relational cues (eg, mental, behavior, and abused in A2; told, self, exhausting, and therapy in A1). In A5, where both classes shared r/Narcissism vocabulary, NPD-related terms largely canceled out as a discriminative signal: even before keyword removal, only 1 of the top 20 positive coefficients was a diagnostic keyword. The features that remained discriminative in A5 were terms associated with distress, self-awareness, and interpersonal difficulty (eg, afraid, hurt, and aware). These represent the residual signal once shared NPD vocabulary is neutralized. This design-dependent variation indicates that the classifier adapts its feature reliance to the available vocabulary contrast rather than depending on a fixed set of keywords. Whether the residual signal in A5 reflects NPD-specific distress patterns or broader emotional distress within the r/Narcissism community remains an empirical question that the current design cannot resolve. A related sampling caveat applies to "aware" specifically, which may reflect self-recognition among users who already identify with NPD-consistent traits rather than NPD-trait expression more generally (see the “Feature Profile and Construct Interpretation” section). Coefficient and LIME analyses apply only to the FSA + LR baseline.

While these results demonstrate the potential of these models, they should be interpreted with caution given the limited dataset size. Further validation across broader online environments and real-world settings is needed.

### Limitations

#### Sample Size and Interpretation of Performance Metrics

When interpreting the results, several limitations should be considered. Our study was based on English-language data, although the approach could also be applied to other languages using embedding models. We used Reddit posts containing at least 100 characters as the data source. Although we expect the models to generalize to other platforms, different media may exhibit distinct linguistic dynamics that should be taken into account.

We identified 185 positively annotated posts, and this research can be extended as the subreddit grows and additional posts are annotated by mental health professionals. Experiments A1, A3, A4, and A5 used 185 positively labeled and 185 negatively labeled posts. Using an 80/20 train-test split resulted in a test set of 74 posts (37 per class). Although it is mathematically possible to achieve perfect scores in some experiments, the perfect precision and recall scores observed may be attributable to the limited test-set size, as correctly classifying a small number of cases can yield perfect performance metrics. This limitation is particularly relevant for experiment A2, in which the control group (r/ImposterSyndrome) contained only 63 posts, resulting in 13 test samples for this class (total test n=50: 37 positive and 13 negative). Therefore, the perfect precision and recall scores (1.0) achieved in this experiment should be interpreted with caution. To account for the statistical uncertainty associated with the small control sample, we calculated a 95% Wilson score CI of 0.772-1.000 for the 13-sample control class.

While the results demonstrate the feasibility of detecting NPD traits using machine learning, we recognize the inherent limitations of a small dataset, including the potential for overfitting. To address this, we balanced the dataset using undersampling (A5 and B5) and SMOTETomek (A2, B2, and setup C) and applied robust validation techniques, including 5-fold cross-validation. The consistent performance across embedding models is encouraging, but further research using larger and more diverse datasets is needed to confirm the generalizability of these findings. This study lays the groundwork for future research exploring scalable approaches for mental health applications in online forums. In the imbalanced experiments, the SMOTETomek step was applied to the feature representation used in each experiment, including transformer embeddings. SMOTE generates minority-class instances by interpolating between neighboring samples, which works well for sparse bag-of-words features but is more difficult to interpret in a dense contextual embedding space, where an interpolated point does not generally correspond to a linguistically coherent representation. The same concern applies to Tomek link removal. Therefore, this theoretical limitation also applies to these experiments.

To assess the practical impact of this limitation, we reran the 30 binary embedding pipelines (A1-A5 and B1-B5 × 3 classifiers) under 3 conditions: baseline SMOTETomek, no resampling, and class_weight=“balanced.” The mean absolute Δ*F*_1_-score between the baseline and no-resampling conditions was 0.006, with a maximum of 0.047. ROC-AUC was similarly stable (maximum |Δ|=0.038). The mean signed Δ*F*_1_-score was +0.000, and the direction of change was mixed across the largest differences, indicating that SMOTETomek neither systematically improved nor degraded performance at this scale. A 2×2 factorial analysis of the 6 A2/B2 cells, where SMOTE was actually applied, isolated the interpolation effect at 0.000 in 5 of the 6 cells, and the remaining cell differed by no more than 0.01 *F*_1_-score. Applying the same protocol to the 9 binary setup C cells (C2-C4 × 3 classifiers, where SMOTETomek was also applied) yielded a mean absolute Δ*F*_1_-score of 0.018, with a maximum of 0.071 and mixed directionality (4 of the 9 cells showed negative changes). ROC-AUC again remained stable (maximum |Δ|=0.024). For the multiclass C1 cells, scored using weighted *F*_1_-score and weighted one-versus-rest ROC-AUC as in Table 10, the Δ*F*_1_-scores were −0.009 (LR), +0.075 (SVM), and +0.074 (RF), whereas ROC-AUC again remained stable (maximum |Δ|=0.021). Thus, although the theoretical concern remains valid, its practical impact on the results presented in Table 10 appears to be limited for this corpus and these embedding models.

Our data collection ended in August 2019, before significant changes in online mental health discourse. The COVID-19 pandemic substantially altered patterns of online self-expression, help-seeking behavior, and mental health discussions across social media platforms. In addition, the emergence of new platforms (eg, TikTok) and changes in Reddit community dynamics may have influenced how individuals discuss narcissism and personality disorders online. These temporal factors may affect the model’s generalizability to current discourse, and validation using post-2019 data is needed before deployment in contemporary settings.

Our best-performing models rely on the OpenAI embedding APIs (text-embedding-ada-002 and text-embedding-3-large), which are proprietary and closed source. This dependency poses challenges for long-term scientific reproducibility because these models may be updated or deprecated by the vendor without notice. By contrast, open-weight alternatives such as BERT or LLaMA offer greater stability for replication studies. However, several open-source embedding models (eg, all-MiniLM and nomic-embed-text) achieved comparable performance in our experiments, providing reproducible alternatives for future implementations.

We conducted the interpretability analysis using the FSA + LR pipeline, which provides direct access to feature weights and coefficients. Detailed feature-level inference focused on the within-subreddit A5 experiment (FSA + LR baseline *F*_1_-score=0.667), in which both the positive and control posts originated from r/Narcissism. This design minimizes subreddit-level stylistic and topical confounding (see the “Subreddit Style Confounds” section), making A5 the most appropriate setting for attributing learned features to NPD-related discourse rather than community membership. Cross-experiment robustness is reported across all 13 binary experiments in Section S3 in Multimedia Appendix 1. A2 and A1 are presented as additional examples (see Sections S4 and S5 in Multimedia Appendix 1), but feature-level construct claims are limited to A5 (see Section S6 in Multimedia Appendix 1). The embedding models that achieved the highest *F*_1_-scores in this study (up to 0.892 for A5) remain opaque; therefore, feature-level interpretability claims apply only to this lower-performing baseline and may not generalize to the embedding models.

LIME provided local, instance-level explanations, but contextual words (eg, school, working, wife, and friends) occupied 53%-79% of the top 5 explanation slots across experiments. This predominance reflects both a TF-IDF sparsity artifact and genuine reliance on subreddit-specific life topics. The coefficient tables show that relational and contextual terms (eg, wife, friends, society, and men) carry substantial positive coefficients for the NPD-positive class. Thus, classification performance appears to reflect a combination of psychologically relevant cues and community-level topical patterns, whose relative contributions cannot be disentangled using the current methodology. Coefficient analysis serves as the primary source of interpretability evidence, whereas LIME provides supplementary local illustrations.

#### Single-Coder Feature Categorization

The 9-category feature-coding scheme was applied by a single coder without assessment of interrater reliability. Therefore, the category distributions presented in Multimedia Appendix 1 should be considered approximate. Section S7 in Multimedia Appendix 1 documents the boundary-case ambiguities and potential bias introduced by the default coding decisions.

We based this research on Reddit posts annotated by psychiatrists (NBA and OZ). Although these annotations provided a reliable foundation for model development, definitive clinical assessment would require formal diagnosis according to DSM-5 criteria followed by analysis of the patients’ online content. In addition, our annotation criteria focused on DSM-5 NPD diagnostic features, which primarily emphasize markers of grandiosity.

In setup B, the positive class comprised 6 label types representing both posts whose authors exhibited NPD traits (L7, L8, and L9) and posts referring to individuals with NPD traits without indicating that the author exhibited those traits (L2, L4, and L6). See Table 5 for the complete label composition and the “Task Complexity and Contextual Information” section for the implications for construct validity. Within r/Narcissism, L4 posts typically describe experiences of interpersonal harm (eg, references to a relative with narcissistic traits). By contrast, setup A provides a more specific classifier for identifying authors who themselves exhibit NPD traits.

#### Statistical Power for Per-Experiment Comparisons

The keyword-removal robustness analysis included 13 binary experiments with test-set sizes ranging from 50 to 204 posts. At these sample sizes, McNemar tests comparing the original-text and keyword-removed models on identical test sets yielded only 1-13 discordant pairs per comparison, which is too few in most cases for the test to reliably detect realistic effect sizes. Accordingly, the bootstrap 95% CIs for the *F*_1_-score differences were wide and frequently included 0. Therefore, a nonsignificant result reflects insufficient statistical power rather than equivalence and should not be interpreted as either confirming or refuting model robustness.

A1 was the only exception. With 13 discordant pairs—the largest number across all experiments—it was the only comparison with sufficient power to detect an effect, and keyword removal resulted in a statistically significant decrease in *F*_1_-score (McNemar *P*=.02). This outcome is expected for a cross-subreddit, keyword-dependent design (see Section S5.3 in Multimedia Appendix 1 for the coefficient profile). However, it does not alter the overall pattern: the robustness argument depends on whether any experiment showed a complete performance collapse after keyword removal, and none did. Thus, the robustness claim is based on the consistent pattern of point estimates across experiments, several of which showed little or no performance degradation, rather than on the results of any individual per-experiment test (see the “What Do These Automated Classifiers Actually Learn?” section).

#### Train/Test Independence

Usernames and other author identifiers were not recorded (see the “Privacy and Confidentiality” section). Consequently, the train-test split was performed at the post level rather than the user level, and user-disjoint partitioning was not possible. As a result, posts from the same author may have appeared in both the training and test sets. This could allow the model to learn author-specific writing styles rather than generalizable NPD-trait markers, potentially inflating performance on the held-out test set, particularly for prolific contributors. As author identifiers were not retained, we could neither quantify this effect nor re-partition the data on a user-disjoint basis. We therefore acknowledge this as a transparency limitation of the reported performance metrics.

#### Control Groups and Comparison With Other Clinical Forums

Our validation of the control subreddits (r/Showerthoughts, r/DeepThoughts, and r/ImposterSyndrome) confirmed minimal evidence of NPD traits, consistent with the expected prevalence in the general population. However, future research could strengthen this validation by annotating larger samples from these control communities. Incorporating additional data sources may also improve the robustness of comparative analyses.

Our control subreddits (r/DeepThoughts and r/Showerthoughts) represent neutral reflective discourse, whereas r/ImposterSyndrome represents self-focused but nongrandiose narratives. Future research should evaluate model performance on communities related to other personality disorders (eg, r/BPD) to assess specificity. However, such analyses would require new data collection, appropriate IRB approval, and disorder-specific annotation schemes and were therefore beyond the scope of this study.

The test-set prevalence (see the “Class Imbalance and Resampling Strategy” section) was substantially higher than the estimated population prevalence of 1%-6%. Consequently, the reported precision and *F*_1_-scores are likely to overestimate performance in real-world deployment. Adjusting precision for prevalence using Bayes theorem, *P* = (π·TPR)/(π·TPR + [1 − π]·FPR), where TPR is true-positive rate, shows the expected effect. For A5 (text-embedding-ada-002 + LR in Table 10; TPR=0.892, FPR=0.108), the adjusted precision is approximately 0.08 at π=0.01, 0.20 at π=0.03, and 0.35 at π=0.06. However, these point estimates are based on a very small number of false positives (4 of 37 in A5) and should therefore be interpreted with caution because of their wide uncertainty.

Propagating the 95% Wilson CI for A5’s FPR (0.043-0.247) through the Bayes formula at π=0.03 yields an adjusted precision of approximately 0.10-0.40. These values should be interpreted as illustrative orders of magnitude rather than as lower bounds for deployment precision. The adjustment also assumes that the test-set TPR and FPR remain unchanged in deployment, a strong assumption given that the negative class in real-world settings would differ from the control subreddits used in this study. By contrast, ROC-AUC and recall are invariant to prevalence. Any operational deployment would therefore require prevalence-aware calibration, such as Platt scaling, threshold tuning, or precision-recall analyses across a defined range of prevalence values, rather than relying on the operating point observed in the test set.

We acknowledge that r/Narcissism contains heterogeneous content, including posts from self-identified individuals with narcissistic traits, help seekers, individuals questioning whether they exhibit such traits, and people discussing others with narcissistic traits in their lives. As a result, the model learns from multiple linguistic contexts related to narcissism rather than from a purely clinical sample of individuals with confirmed diagnoses. However, the use of psychiatrist-validated labels (L7 and L8) helps ensure that the model captures clinically relevant linguistic patterns despite this source heterogeneity.

#### Feature Profile and Construct Interpretation

The classifier’s feature profile is design dependent (see the “What Do These Automated Classifiers Actually Learn?” section). The cross-subreddit experiments relied substantially on diagnostic keywords in the original model, whereas the within-subreddit A5 experiment discriminated primarily on distress, self-awareness, and relational cues once shared NPD vocabulary was neutralized. This variation cautions against adopting a single construct interpretation across all 13 experiments. Sections S5.3, S6.5, and S9 in Multimedia Appendix 1 present detailed evidence of these design-dependent feature profiles. For the scope of the keyword removal list and the potential influence of unlisted community jargon, see the “What Do These Automated Classifiers Actually Learn?” section and Section S9 in Multimedia Appendix 1.

#### Subreddit Style Confounds

We acknowledge that stylistic differences between subreddits may influence classification when control posts originate from communities such as r/Showerthoughts or r/DeepThoughts, which have writing conventions that differ from those of r/Narcissism. Experiments A5 and B5 use posts exclusively from r/Narcissism for both the positive and negative classes, thereby eliminating cross-subreddit stylistic variation—the same design feature that motivates A5 as the primary setting for the feature-level interpretability analysis (see Section S6 in Multimedia Appendix 1). A5 (n=370) achieved an accuracy of 0.892 and an *F*_1_-score of 0.892, substantially exceeding chance performance even after removing subreddit-level stylistic variation. A content-versus-style feature ablation analysis (cross-validated and reproducible using the published code package) showed that, within A5, content features (word TF-IDF semantics and sentiment) achieved 80.6% accuracy compared with 68.2% for style features (orthographic and punctuation statistics plus part-of-speech distributions), a 12.4-percentage-point advantage for content (SD 5.9 across folds). As the resampled cross-validation folds were not independent, we report this difference without a significance test. Although style features alone performed above the 50% chance baseline, content features provided substantially better discrimination. In the combined model, RF feature importance was 97% content and 3% style. However, because content and style features overlap, this marginal importance likely understates the standalone contribution of style, which is more directly reflected by the ablation analysis.

The advantage of content features is not attributable to cross-subreddit confounding. The difference in accuracy between content and style features was similar in the cross-subreddit experiments (mean 11.8 percentage points across A1-A3) and the within-subreddit A5 experiment (12.4 percentage points).

In the cross-subreddit experiments, the content signal included both established diagnostic vocabulary and community-specific terms. In the within-subreddit setting, both content and style signals may reflect community-level discourse norms in addition to trait-relevant variation. Future research could use adversarial debiasing and validation across diverse mental health communities (eg, r/BPD, r/depression, and r/anxiety) to further disentangle trait-relevant markers from community-level discourse norms.

### Comparison With Prior Work

Sumner et al [30] and Preoțiuc-Pietro et al [31] used machine learning to detect Dark Triad traits on Twitter, where posts were limited to 140 characters at the time of those studies.

Rathner et al [37] applied linear regression to LIWC scores derived from essays and correlated the results with Narcissistic Personality Inventory (dichotomous-response version) scores. They analyzed linguistic markers to predict narcissism and depression.

Our study focuses on predicting NPD traits from Reddit posts containing at least 100 characters and, unlike prior work, develops a classification model rather than a regression model.

In our previous work [28], we developed classification models to detect suicidal ideation using sentiment analysis, TF-IDF, and LIWC features on manually annotated Reddit data. In this study, we introduced text embedding models and found that they substantially improved classification performance. We also used multilabel annotations, enabling us to define different classification tasks based on different label distributions across classes.

### Ethical Implications and Limitations

It is important to acknowledge the ethical concerns and potential for misuse. Such a system could be used for nonclinical purposes, such as targeted advertising, workplace surveillance, or malicious profiling of individuals without their consent. These misuses could lead to discrimination, unfair treatment, or social alienation. To mitigate these risks, we emphasize that such a system should be used only under strict ethical guidelines and in collaboration with qualified mental health professionals.

Automated detection of symptom signals from social media carries a risk of misclassification: false positives may stigmatize users, whereas false negatives may fail to identify individuals in need. In addition, algorithmic bias may amplify disparities if the model overrepresents or underrepresents particular demographic groups [52]. We therefore caution against any standalone clinical deployment without rigorous validation, human oversight, and ongoing monitoring for unintended harms [53].

### Conclusions

This study investigated whether machine learning models could detect linguistic markers of NPD traits in Reddit posts. Using a psychiatrist-annotated corpus of 966 posts, we compared frequency- and embedding-based classifiers across 14 experimental configurations. The embedding models achieved *F*_1_-scores ranging from 0.744 to 1.0 across the binary experiments (the multiclass C1 experiment achieved a weighted *F*_1_-score of 0.685). The 2 OpenAI embedding models (text-embedding-ada-002 and text-embedding-3-large) performed best overall and were essentially tied, with mean *F*_1_-scores of approximately 0.90. The models successfully distinguished both posts authored by individuals exhibiting NPD traits (setup A) and posts in which a person exhibiting NPD traits was either the author or the subject (setup B). After removing explicit NPD-related keywords (six-family test), the mean relative *F*_1_-score decreased by 3.6% for the FSA + LR baseline, 1.9% for the all-MiniLM-L6-v2 embedding model, and 0.8% for the text-embedding-3-large embedding model, with no experiment showing a complete performance collapse (the cross-subreddit A1 experiment showed measurable but noncollapsing dependence on keywords; see the “Statistical Power for Per-Experiment Comparisons” section). A companion interpretability analysis (Multimedia Appendix 1) of the FSA + LR baseline showed that, after diagnostic terms were removed, the highest-ranked predictive features shifted toward emotional, cognitive, and relational cues. These findings demonstrate the feasibility of automated detection of NPD traits in online forum text and provide a foundation for future computational research on personality disorder assessment (see the “Feature Profile and Construct Interpretation” section for the boundaries of construct validity).

## Appendix

Multimedia Appendix 1 Interpretability analysis using Local Interpretable Model-Agnostic Explanations (LIME).

Multimedia Appendix 2 Technical details.

Multimedia Appendix 3 Checklist for adherence to guidelines for developing and reporting machine learning predictive models in biomedical research.

Multimedia Appendix 4 Details of the experiments.

Multimedia Appendix 5 Annotation reliability metrics.

Multimedia Appendix 6 Experiment results.

## Abbreviations

AC1: Gwet first-order agreement coefficient
AUC: area under the curve
BERT: Bidirectional Encoder Representations from Transformers
DSM-5: Diagnostic and Statistical Manual of Mental Disorders, Fifth Edition
FMINAREK: Fen Bilimleri ve Mühendislik Alanları İnsan Araştırmaları Etik Kurulu
FPR: false-positive rate
FSA: frequency- and sentiment-based analysis
IRB: institutional review board
LIME: Local Interpretable Model-Agnostic Explanations
LIWC: linguistic inquiry and word count
LLaMA3: Large Language Model Meta AI 3
LR: logistic regression
NLP: natural language processing
NPD: narcissistic personality disorder
RF: random forest
ROC: receiver operating characteristic
SMOTE: Synthetic Minority Over-sampling Technique
SVM: support vector machine
TF-IDF: term frequency-inverse document frequency
TPR: true-positive rate
XGB: Extreme Gradient Boosting

## Data Availability

The annotated dataset generated during this study is not publicly available because of ethical considerations related to the sensitive nature of the mental health–related content and the potential for reidentification of Reddit users discussing personal psychological experiences. As the posts may contain self-disclosures regarding NPD traits, clinical diagnoses, and personal relationships, unrestricted access could pose a risk to the original posters, who did not consent to research use of their content. In addition, the trained classification models could potentially be misused for nonclinical profiling or surveillance if deployed without appropriate oversight.

The annotated dataset is available from the corresponding author (AEA) upon reasonable request. Requests will be evaluated for legitimate research purposes and should include (1) institutional affiliation, (2) a description of the intended research use, (3) evidence of IRB approval or ethics review for the proposed research, and (4) an agreement to use the data solely for research purposes under appropriate clinical supervision. The code used for model training and evaluation is publicly available at the project’s GitHub repository [54].
