# Supplementary material for: Detecting Narcissistic Personality Disorder Traits on Forums: Proof-of-Concept Study
Source: J Med Internet Res. 2026 Jul 29;28:e75799. doi: 10.2196/75799 (PMC13469838; doi:10.2196/75799)
Supplement: Supplementary file 1 [file jmir_v28i1e75799_app1.pdf]

Multimedia Appendix 5: Model Interpretability Analysis

Contents

1. Analytical Objective **2**

2. Methodology **3**

Pipeline 3

Keyword Removal 3

LIME Configuration 3

3. Cross-Experiment Performance Summary **4**

4. Showcase Analysis: A2 (Narcissism+ vs ImposterSyndrome) **4**

4.1 Why A2 4

4.2 Keyword Audit 5

4.3 Three-Model Ablation 5

4.4 Coefficient Analysis (Top-20 Positive) 6

4.5 LIME Anchor Cases 8

4.6 Aggregate LIME Analysis 11

5. Supporting Analysis: A1 (Narcissism+ vs DeepThoughts) **12**

5.1 Keyword Audit 12

5.2 Three-Model Ablation 13

5.3 Coefficient Analysis 13

5.4 LIME Anchor Cases 16

5.5 Aggregate LIME Analysis 19

6. Hard Test: A5 (Narcissism+ vs Narcissism-) **20**

6.1 Why A5 is the Hardest Test 20

6.2 Keyword Audit 21

6.3 Three-Model Ablation 21

6.4 Key Finding 22

6.4.1 Random Feature-Removal Baseline 22
6.5 Coefficient Analysis 22
6.6 LIME Anchor Cases 25
6.7 Aggregate LIME Analysis 28

7. LIME Structural Limitation **29**

8. Embedding-Based Keyword Robustness Check **30**

9. Calibrated Conclusion **31**

10. Reproducibility **33**

1. Analytical Objective

A recurring concern with text classifiers trained on labeled community data is that they may rely primarily on explicit diagnostic keywords (e.g., 'narcissist', 'NPD', 'diagnosed') rather than linguistically meaningful cues. This appendix addresses that concern through a systematic three-tier analysis design:

1. **Cross-experiment performance summary** across all 13 binary experiments (excluding C1, which uses multiclass 3-label design), comparing original and keyword-removed models.
2. **Deep-tier analysis** for three showcase experiments (A2, A1, A5), including three-model ablation (original, no-keyword, no-surrogate), coefficient analysis, LIME anchor cases, and aggregate LIME category distributions.
3. **Structural limitation analysis** documenting a consistent LIME+TF-IDF artifact across all deep-tier experiments.

The keyword family list was expanded to include 'self absorbed' (and its variants), bringing the total to 6 keyword families: narcissism, npd, disorder, personality, self absorbed, and diagnose. In an earlier version of the A5 analysis, 'self absorbed' appeared as the top-1 feature in the original model's coefficient ranking; the current A5 analysis uses the expanded keyword list, so 'self absorbed' no longer appears in the coefficient tables below. This term has been added to the keyword removal list, confirmed absent from all no-keyword models (see keyword audits in Sections 4.2, 5.1, and 6.2), and the no-keyword analyses below demonstrate that the model retains meaningful signal without it.

As documented in Section 7, LIME with TF-IDF features proved less informative than expected for local explanations due to a structural sparsity artifact. The logistic regression coefficient analysis therefore carries the primary interpretability burden, with LIME providing supplementary local illustration.

All interpretability claims in this appendix apply exclusively to the FSA + Logistic Regression pipeline. Performance robustness may be discussed across model families, but direct feature-level interpretation is limited to this transparent, directly inspectable model. Embedding-based models were excluded from interpretability analysis because their feature representations are

high-dimensional and non-sparse, lacking the one-to-one correspondence between input tokens and feature dimensions that makes logistic regression coefficients and LIME text explanations directly interpretable.

2. Methodology

Pipeline

Each experiment uses a Feature-Set Ablation (FSA) pipeline with Logistic Regression as the classifier. Text features are extracted via TF-IDF (unigrams through trigrams) from post selftext and title, with additional sentiment features (TextBlob polarity and subjectivity, computed per field, yielding 4 features). The pipeline uses a ColumnTransformer (FeatureUnion-style) to combine TF-IDF and sentiment features before classification.

Keyword Removal

Six keyword families are removed for the no-keyword variant:

Family	Example terms
narcissism	narcissist, narcissism, narcissistic, narcs
npd	npd
disorder	disorder
personality	personality
self_absorbed	self absorbed, self-absorbed
diagnose	diagnosed, diagnoses, diagnosing, diagnosis, diagnostic

Removal is verified post-hoc via keyword audit (confirming zero remaining occurrences in the no-keyword training and test sets).

LIME Configuration

Local explanations use LIME (Local Interpretable Model-agnostic Explanations) with the following settings:

- 1,000 perturbation samples per explanation (5,000 on retry if unstable)
- Top-10 features per explanation
- 3 stability runs per anchor case (seeds: 42, 123, 456)
- Anchor cases retained only if stability overlap >= 3 of top-5 features across runs
- Unstable cases are flagged and excluded from core figures or labeled explicitly

Note on aggregate LIME slot counts: The aggregate LIME analysis reports the total number of top-5 feature slots across all test posts. This count may be less than N x 5 because some posts contain fewer than 5 non-zero TF-IDF features after keyword removal (particularly short posts or posts whose vocabulary is dominated by stop words), yielding fewer than 5 LIME explanation features.

3. Cross-Experiment Performance Summary

Experiment	N (test)	Original F1	No-Keyword F1	Delta F1	No-Surrogate F1
A1	74	0.853	0.725	-15.0%	0.732
A2	50	1.000	0.987	-1.3%	0.973
A3	74	0.889	0.783	-12.0%	—
A4	74	0.776	0.771	-0.6%	—
A5	74	0.667	0.620	-7.0%	0.630
B1	204	0.923	0.873	-5.5%	—
B2	115	0.975	0.954	-2.1%	—
B3	204	0.939	0.861	-8.2%	—
B4	204	0.700	0.697	-0.4%	—
B5	183	0.570	0.618	+8.4%	—
C2	193	0.803	0.799	-0.5%	—
C3	151	0.837	0.852	+1.8%	—
C4	121	0.634	0.610	-3.8%	—

Aggregate (n=13): mean F1 drop = -0.032 (SD=0.049, SE=0.014), median = -0.021, range = [-0.128, 0.048]. The standard deviation reflects genuine variability across experimental designs rather than measurement noise. We report these as descriptive aggregates rather than a formal significance test: the 13 experiments are not independent (the A-, B-, and C-series each share a common positive class drawn from the same post pool), so a one-sample test treating the deltas as independent would understate the true standard error. The mean relative F1 drop is 3.6%.

Key finding: The impact of keyword removal varies substantially by experimental design. Some experiments (A2, A4, C2 and B4) show near-zero degradation, suggesting the model relies on non-keyword features. A few (C3 and B5) slightly improve after keyword removal. Others (A1, A3, B3, A5, B1, C4 and B2) show moderate to large drops (2.1–15.0%), consistent with partial keyword dependence. No experiment collapses entirely, indicating that all models capture some signal beyond keywords.

Note: C1 is excluded from this analysis because it uses a multiclass (3-label) design that is not directly comparable to the binary experiments.

Caveat: Test set sizes vary substantially (50–204 posts). For smaller test sets (A-series: 50–74 posts), individual F1 deltas carry substantial uncertainty; bootstrap confidence intervals (reported in the deep-tier sections) should be consulted before drawing conclusions about any single experiment.

4. Showcase Analysis: A2 (Narcissism+ vs ImposterSyndrome)

4.1 Why A2

A2 contrasts r/NPD (Narcissism+) with r/ImposterSyndrome, representing a comparison between two distinct mental health communities. This experiment is an ideal showcase because:

- The original model achieves perfect classification (F1 = 1.000), providing a strong baseline to test whether keyword removal degrades performance.
- The two communities discuss different psychological experiences, so meaningful linguistic differences should exist beyond diagnostic keywords.
- Near-zero keyword dependency suggests the model captures genuine content differences.

Caveat: Because A2 compares two topically distinct communities, near-perfect classification may partly reflect topical separability rather than linguistically sophisticated feature learning. The A5 within-subreddit analysis (Section 6) provides a more stringent test of the model's ability to learn beyond keywords.

4.2 Keyword Audit

Family	Pre-removal Count	Post-removal Count	Status
narcissism	667	0	Verified
npd	105	0	Verified
disorder	37	0	Verified
personality	58	0	Verified
diagnose	53	0	Verified
self_absorbed	6	0	Verified

4.3 Three-Model Ablation

Test set size: N = 50.

Model	F1	Accuracy	Precision	Recall
Original	1.000	1.000	1.000	1.000
No-keyword	0.987	0.980	0.974	1.000
No-surrogate	0.973	0.960	0.973	0.973

Bootstrap 95% CIs and McNemar tests:

Comparison	F1 Delta 95% CI	McNemar p	N discordant
Original vs No-keyword	[0.000, 0.045]	1.000	1
No-keyword vs No-surrogate	[0.000, 0.047]	1.000	1

Note on McNemar power: With very few discordant pairs (1–13 across experiments), McNemar's test has low statistical power. Non-significant p-values should not be interpreted as evidence of equivalence; they reflect insufficient power to detect differences at these sample sizes.

Keyword removal reduces F1 by 0.013 (-1.3%). Further surrogate removal reduces F1 by an additional 0.014. The model retains strong performance (F1 = 0.973) even after removing both keyword families and prominent surrogates, suggesting it captures robust non-keyword signals.

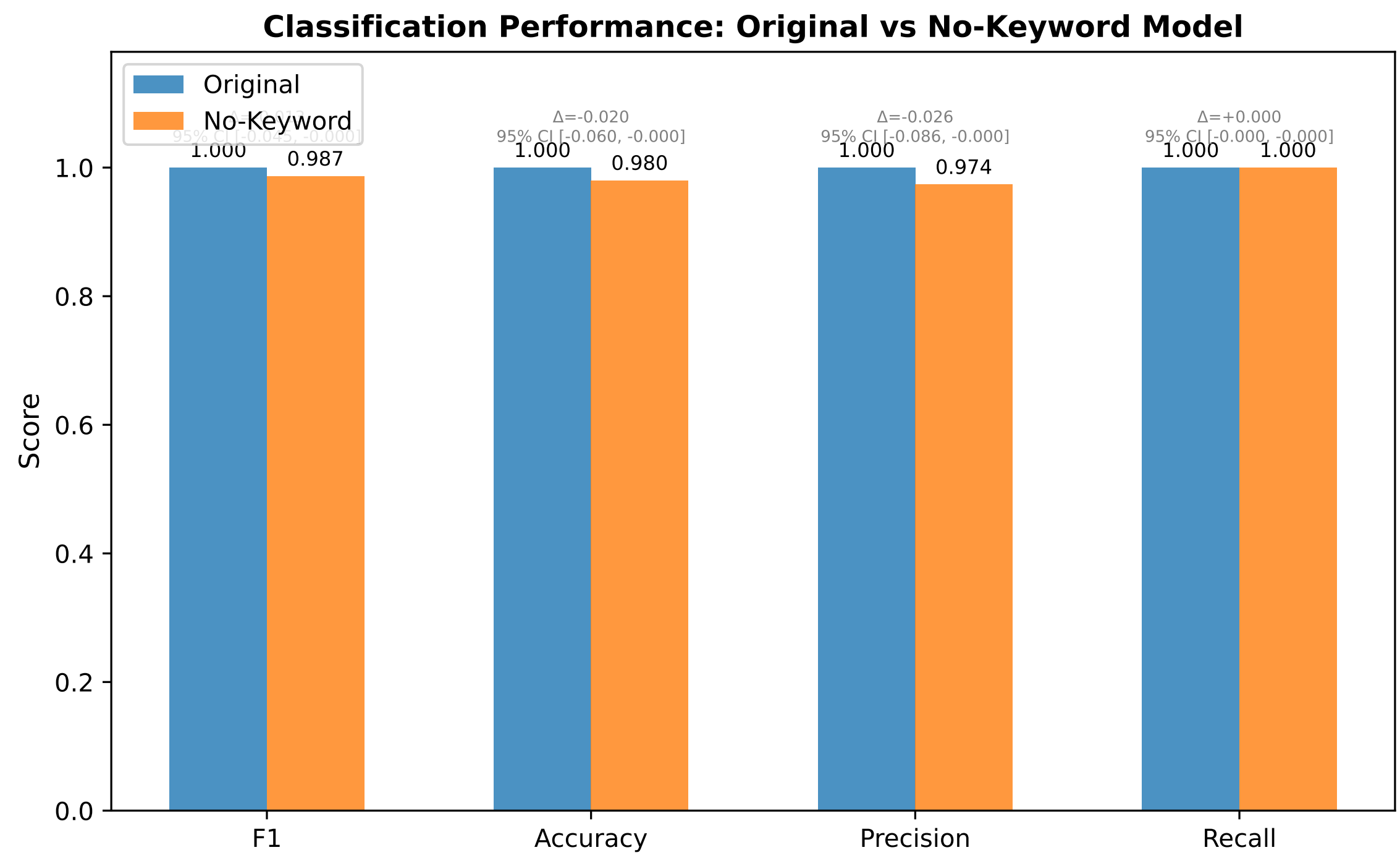

Figure 1: Classification performance: original vs no-keyword model (A2)

4.4 Coefficient Analysis (Top-20 Positive)

Original model — top-20 positive coefficients:

Rank	Feature	Coefficient
1	narcissism	0.2282
2	narcissist (selftext)	0.2219
3	narcissistic (selftext)	0.2184
4	important	0.2171
5	telling	0.2067
6	society	0.1965
7	social	0.1945
8	effort	0.1924
9	normal	0.1920
10	narcissist (title)	0.1908
11	friends	0.1895
12	say	0.1803
13	narcissistic (title)	0.1781
14	care	0.1779
15	narcissists	0.1699
16	narc	0.1678
17	npd	0.1635
18	anger	0.1630
19	loved	0.1612
20	trust	0.1598

No-keyword model — top-20 positive coefficients:

Rank	Feature	Coefficient
1	mental	0.2257
2	important	0.2159
3	behavior	0.2041
4	issues	0.2011
5	society	0.1892
6	social	0.1705
7	normal	0.1681
8	try	0.1660
9	friends	0.1582
10	empathy	0.1579
11	effort	0.1563
12	abused	0.1524
13	view	0.1488
14	needed	0.1467
15	usually	0.1459
16	loved	0.1455
17	care	0.1441
18	person	0.1430
19	act	0.1415
20	confused	0.1394

Category distribution shift (top-20 positive features):

Category	Original Count	No-Keyword Count	Delta
Explicit diagnostic term	8	0	-8
Lexical surrogate / near-synonym	0	1	+1
Emotional / affective cue	3	4	+1
Cognitive / metacognitive	1	3	+2
Relational / interpersonal	3	3	+0
Self-referential / self-focus	0	0	+0
Behavioral / action-oriented	1	3	+2
Contextual / topical	4	6	+2
Sentiment score (TextBlob)	0	0	+0

Observed shift: Explicit diagnostic terms: 8 -> 0 in top-20 positive. Broader cues (emotional + cognitive + relational + self-referential + behavioral) collectively: 8 -> 13. This redistribution is largely mechanical: removing 8 diagnostic terms from a fixed top-20 necessarily promotes the next-ranked features, which are non-diagnostic by construction; the coefficient analysis above is more informative than the category count shift.

Boundary-case note: 'empathy' (no-keyword model, rank 10) is coded as a lexical surrogate because lack of empathy is a core DSM-5 criterion for NPD. Coding it as contextual/topical would change the no-keyword surrogate count from 1 to 0; this ambiguity is one instance of the coding boundary decisions discussed in Section 7.

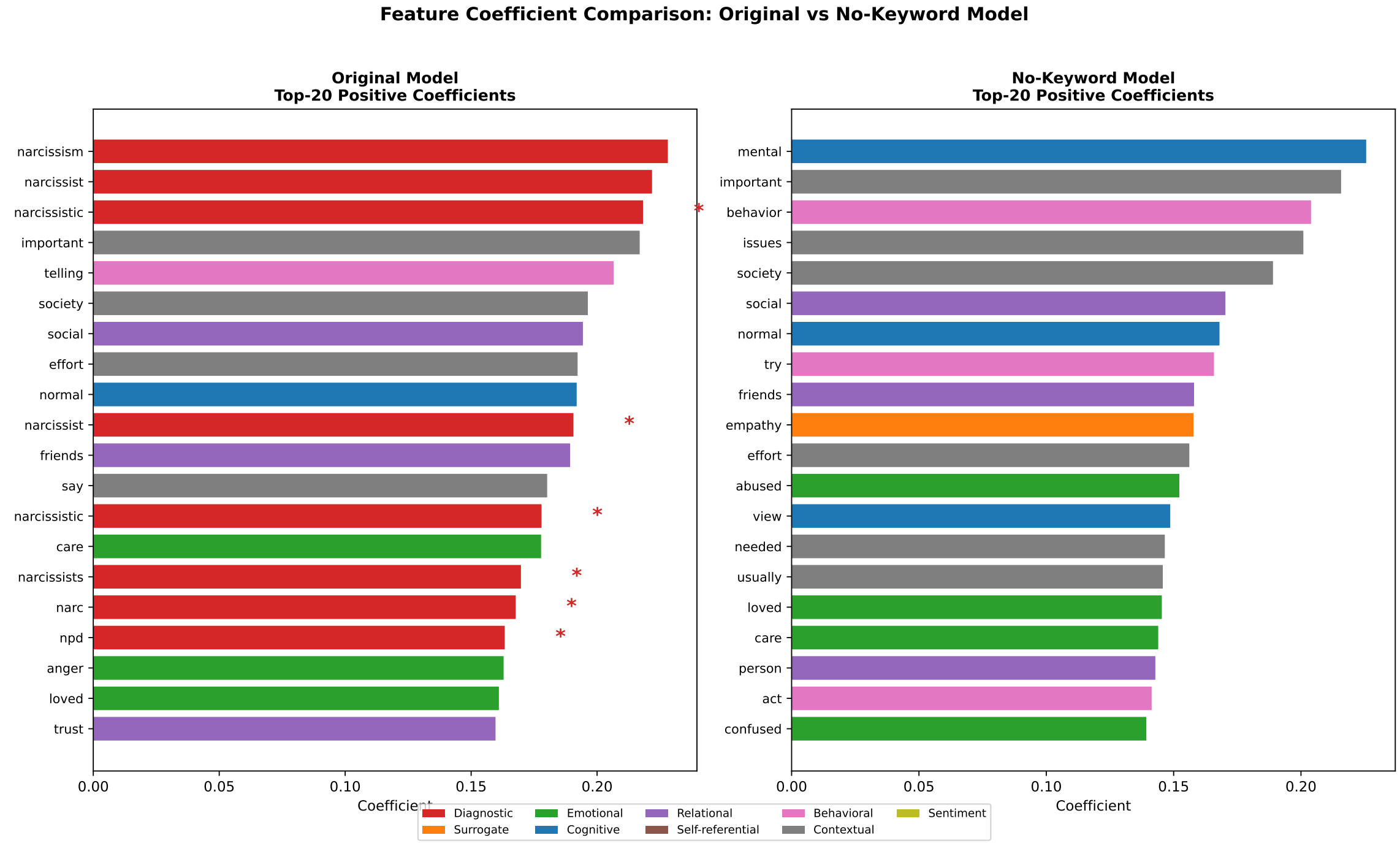

Figure 2: Top-20 positive coefficient comparison: original vs no-keyword model (A2)

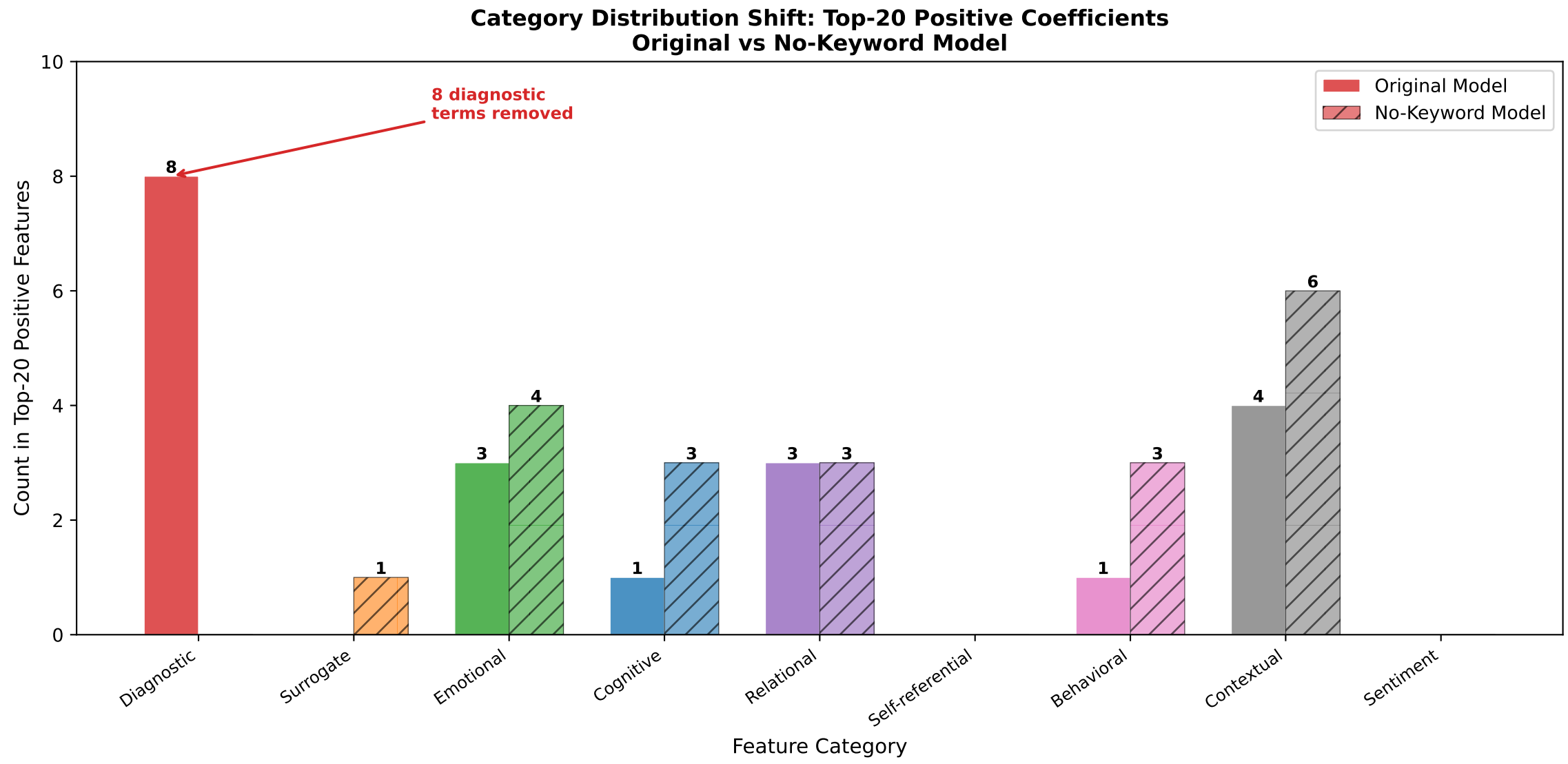

Figure 3: Category distribution shift in top-20 positive coefficients (A2)

4.5 LIME Anchor Cases

The following table shows LIME explanations for held-out test posts, comparing original and no-keyword model explanations. Each case was validated with 3 stability runs.

Post 103 (true=1, pred_nokey=1, prob=1.0000, STABLE)

> *If the isn't harming you, why not give them some leeway for what they can't...*

- Original top-5: effort (+0.0016), society (+0.0016), say (+0.0014), narc (+0.0013), blame

(+0.0013)

- No-keyword top-5: effort (+0.0019), abused (+0.0019), unless (+0.0017), got (-0.0015), issues (+0.0013)

Post 228 (true=1, pred_nokey=1, prob=0.9999, STABLE)

> *Nearly middle-aged and only now realizing I have . Beyond embarrassed, I'm so...*

- Original top-5: Yes (+0.0187), yes (+0.0177), working (-0.0142), emotionally (+0.0127), issues (+0.0120)
- No-keyword top-5: Yes (+0.0046), yes (+0.0045), issues (+0.0033), mental (+0.0032), working (-0.0030)

Post 116 (true=1, pred_nokey=1, prob=0.5304, FLAGGED UNSTABLE)

> *Please just hear me out. I'm close to tears and have been struggling so much...*

- Original top-5: school (-0.1385), friends (+0.1373), feel (-0.1203), feeling (-0.1027), realize (+0.0853)
- No-keyword top-5: school (-0.1542), friends (+0.1327), feeling (-0.1115), feel (-0.1114), covert (+0.0994)

Post 22 (true=0, pred_nokey=1, prob=0.6188, FLAGGED UNSTABLE)

> *Hi all, this one happened at my job. I've been working at this place for a...*

- Original top-5: deserve (-0.1884), working (-0.1541), know (-0.1535), thinking (+0.1408), new (-0.0904)
- No-keyword top-5: thinking (+0.2175), deserve (-0.1554), make (+0.1125), working (-0.0966), feel (-0.0848)

Post 20 (true=0, pred_nokey=0, prob=0.0000, FLAGGED UNSTABLE)

> *This keeps me from building the career I studied for. Back when I was at uni,...*

- Original top-5: Syndrome (-0.0239), Imposter (-0.0220), experience (-0.0219), working (-0.0186), career (-0.0186)
- No-keyword top-5: Syndrome (-0.0437), Imposter (-0.0393), career (-0.0351), experience (-0.0320), school (-0.0286)

(Note: Post 20 has stability overlap of 0 across 3 runs – no feature appeared consistently in the top-5. The displayed features are from a single seed and should not be interpreted as reliable.)

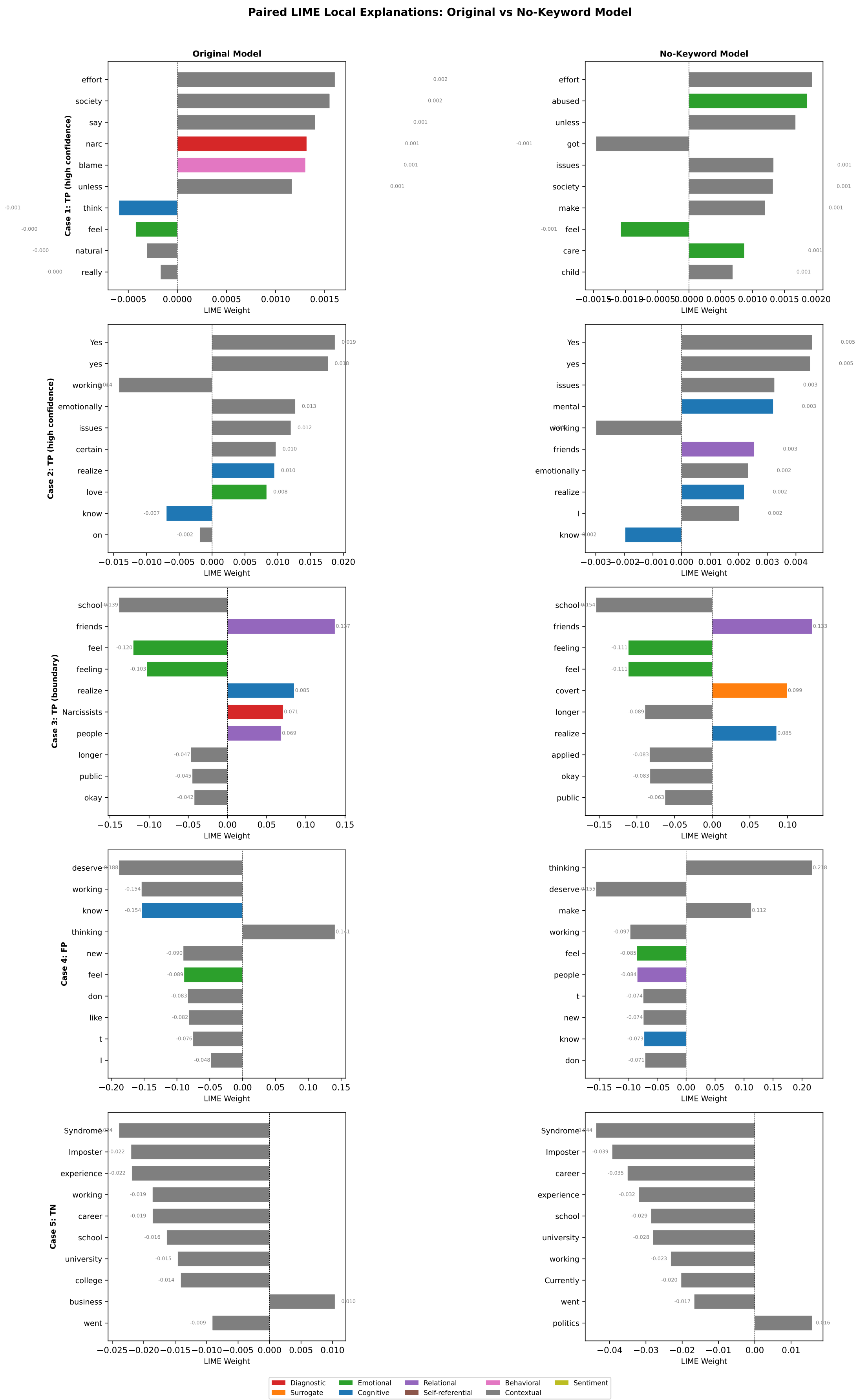

Figure 4: Paired LIME local explanations: original vs no-keyword model (A2)

4.6 Aggregate LIME Analysis

Aggregate LIME analysis across 50 no-keyword test posts (207 total top-5 feature slots):

Category distribution:

Category	Count	Fraction
Lexical surrogate / near-synonym	6	2.9%
Emotional / affective cue	26	12.6%
Cognitive / metacognitive	15	7.2%
Relational / interpersonal	24	11.6%
Self-referential / self-focus	6	2.9%
Behavioral / action-oriented	17	8.2%
Contextual / topical	113	54.6%

Contextual/topical features account for 54.6% of all LIME top-5 slots. This high fraction is a structural characteristic of LIME with TF-IDF features (see Section 7). Of the contextual/topical slots, 84 (40.6% of all slots) were assigned by default (not in the pre-specified coding guide).

Top-10 most frequent features in LIME top-5 (no-keyword model):

Rank	Feature	Count
1	try	7
2	person	7
3	usually	6
4	friends	6
5	issues	5
6	make	5
7	realize	5
8	care	5
9	self	5
10	relationships	5

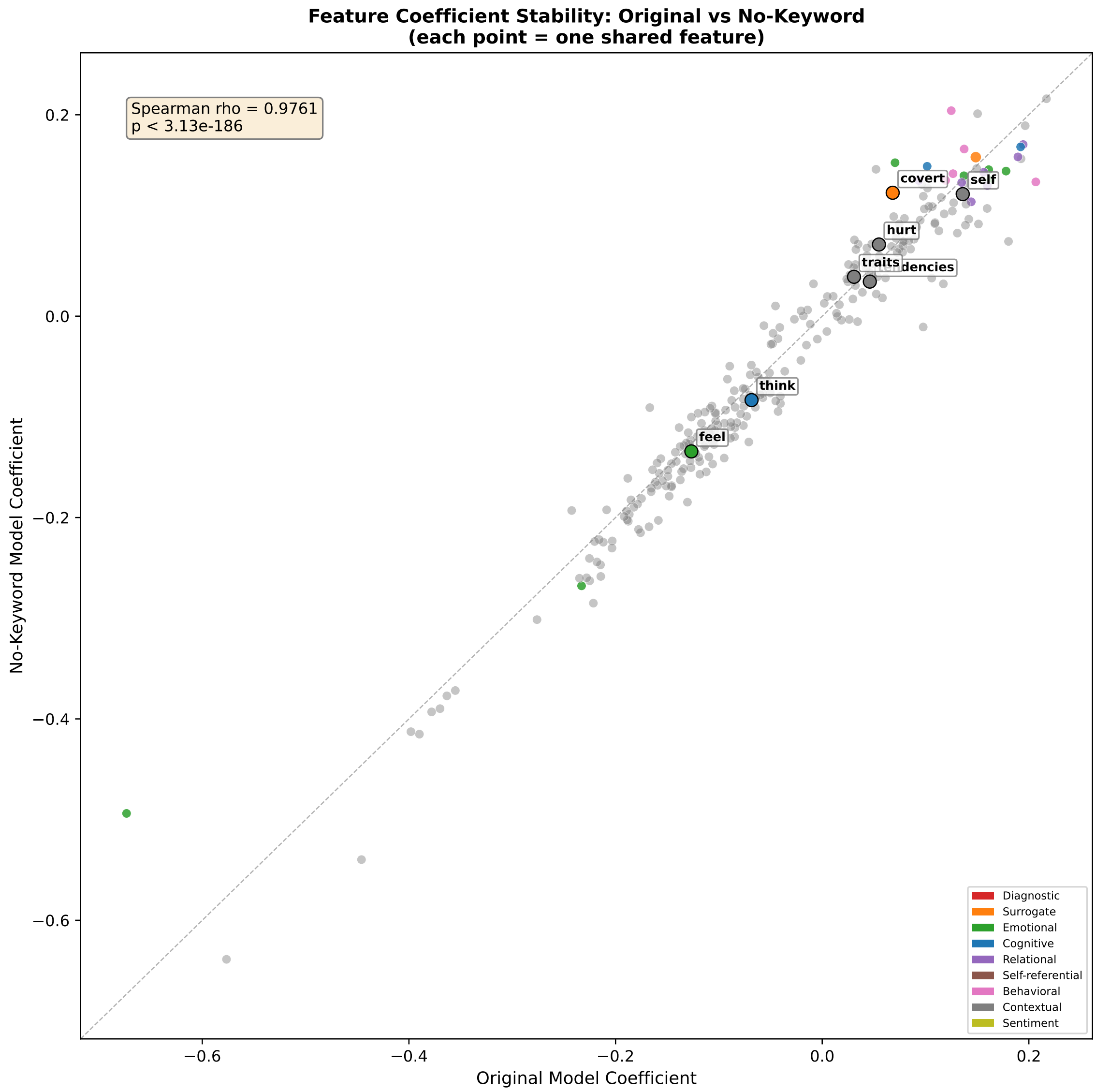

Figure 5: Feature coefficient scatter plot: original vs no-keyword (A2)

5. Supporting Analysis: A1 (Narcissism+ vs DeepThoughts)

A1 contrasts r/NPD with r/DeepThoughts, a general-purpose reflective community. This experiment shows the largest F1 drop among deep-tier experiments (-15.0%), making it informative for understanding which features emerge when keyword-based signals are removed.

5.1 Keyword Audit

Family	Pre-removal Count	Post-removal Count	Status
narcissism	667	0	Verified
npd	105	0	Verified
disorder	37	0	Verified
personality	62	0	Verified
diagnose	53	0	Verified
self_absorbed	6	0	Verified

5.2 Three-Model Ablation

Test set size: N = 74.

Model	F1	Accuracy	Precision	Recall
Original	0.853	0.865	0.935	0.784
No-keyword	0.725	0.743	0.781	0.676
No-surrogate	0.732	0.743	0.765	0.703

Bootstrap 95% CIs and McNemar tests:

Comparison	F1 Delta 95% CI	McNemar p	N discordant
Original vs No-keyword	[0.024, 0.242]	0.022	13
No-keyword vs No-surrogate	[-0.055, 0.030]	1.000	2

Keyword removal reduces F1 by 0.128. Further surrogate removal changes F1 only marginally (F1 0.732 vs 0.725; the no-keyword vs no-surrogate difference is within sampling noise — bootstrap CI spans zero), indicating that residual surrogates contribute negligibly beyond the keyword families.

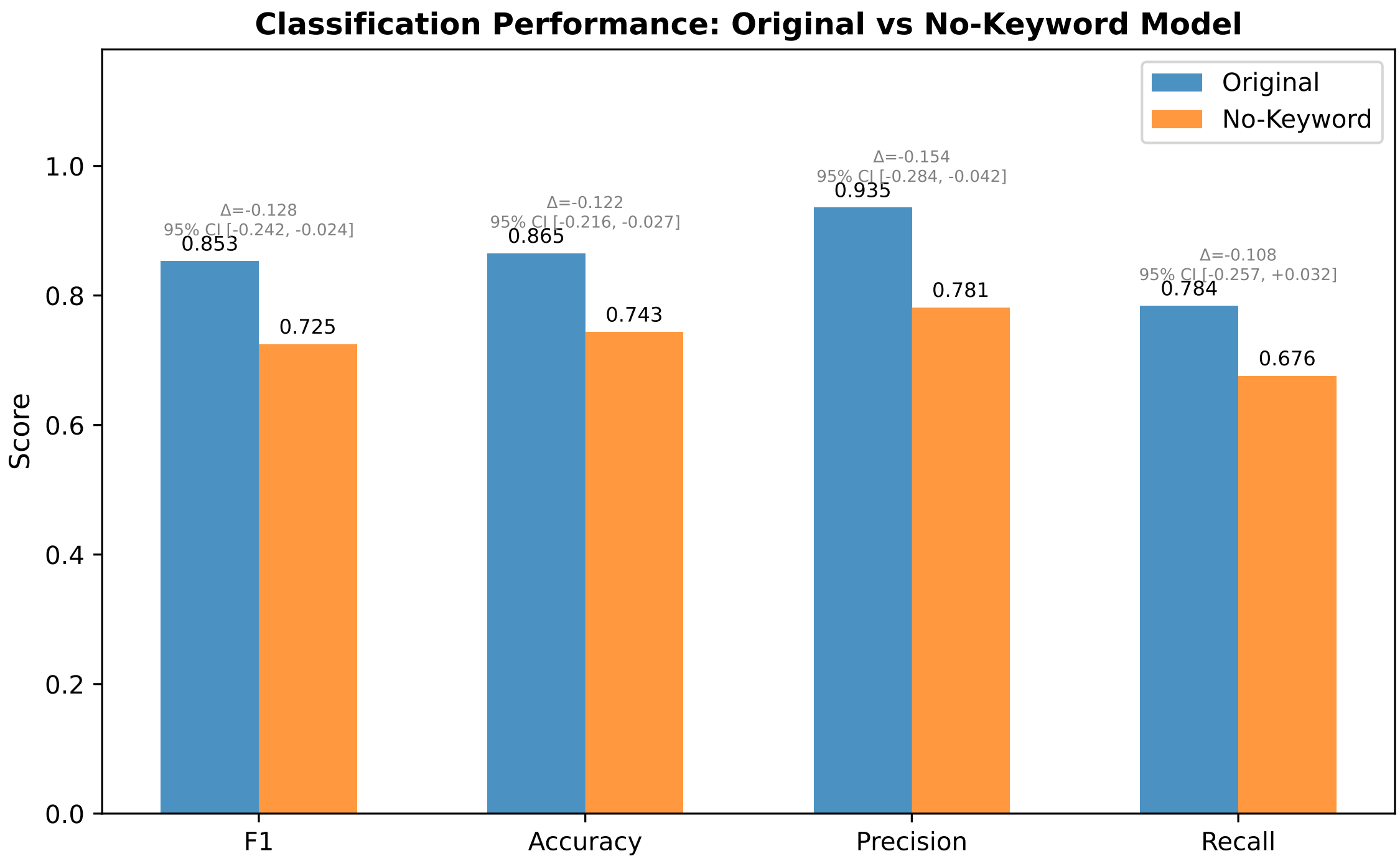

Figure 6: Classification performance: original vs no-keyword model (A1)

5.3 Coefficient Analysis

Original model — top-20 positive coefficients:

Rank	Feature	Coefficient
1	narcissists	0.9281
2	npd	0.4479
3	narcissist (title)	0.4376
4	sources	0.4204
5	narcissist (selftext)	0.4145
6	narcissism (selftext)	0.3994
7	narcissism (title)	0.3787
8	narcissistic	0.3457
9	exhausting	0.3265
10	told	0.3184
11	covert	0.3003
12	care people	0.2933
13	effort	0.2932
14	online	0.2925
15	looking	0.2891
16	superior	0.2819
17	narcs	0.2713
18	fault	0.2683
19	ex	0.2630
20	possibly	0.2549

No-keyword model — top-20 positive coefficients:

Rank	Feature	Coefficient
1	way	0.8790
2	smart	0.5282
3	sources	0.5186
4	told	0.4828
5	studies	0.4523
6	covert	0.4361
7	help	0.4215
8	tendencies	0.4109
9	self	0.4007
10	abused	0.3918
11	complex	0.3764
12	want change	0.3668
13	looking	0.3599
14	sent_selftext_subjectivity	0.3497
15	possibly	0.3455
16	exhausting	0.3400
17	care people	0.3370
18	shit	0.3323
19	therapy	0.3323
20	effort	0.3224

Category distribution shift (top-20 positive features):

Category	Original Count	No-Keyword Count	Delta
Explicit diagnostic term	8	0	-8
Lexical surrogate / near-synonym	1	2	+1
Emotional / affective cue	1	3	+2
Cognitive / metacognitive	1	3	+2
Relational / interpersonal	2	1	-1
Self-referential / self-focus	0	1	+1
Behavioral / action-oriented	1	4	+3
Contextual / topical	6	5	-1
Sentiment score (TextBlob)	0	1	+1

Observed shift: Explicit diagnostic terms: 8 -> 0 in top-20 positive. Broader cues (emotional + cognitive + relational + self-referential + behavioral) collectively: 5 -> 12. This redistribution is largely mechanical: removing 8 diagnostic terms from a fixed top-20 necessarily promotes the next-ranked features, which are non-diagnostic by construction; the coefficient analysis above is more informative than the category count shift.

Residual surrogate note: 'covert' (rank 6) is a residual lexical surrogate – shorthand for 'covert narcissist' in the NPD community. Its presence at a high rank in the no-keyword model indicates that some quasi-diagnostic signal persists even after keyword removal. The surrogate-removal step addresses this by removing such terms in the third ablation tier.

Boundary-case note: 'superior' (original model, rank 16) is coded as contextual/topical, but it is directly relevant to the DSM-5 grandiosity criterion for NPD. Coding it as a lexical surrogate would change the A1 original diagnostic term count from 8 to 9. This is one of the ambiguous boundary decisions noted in Section 7.

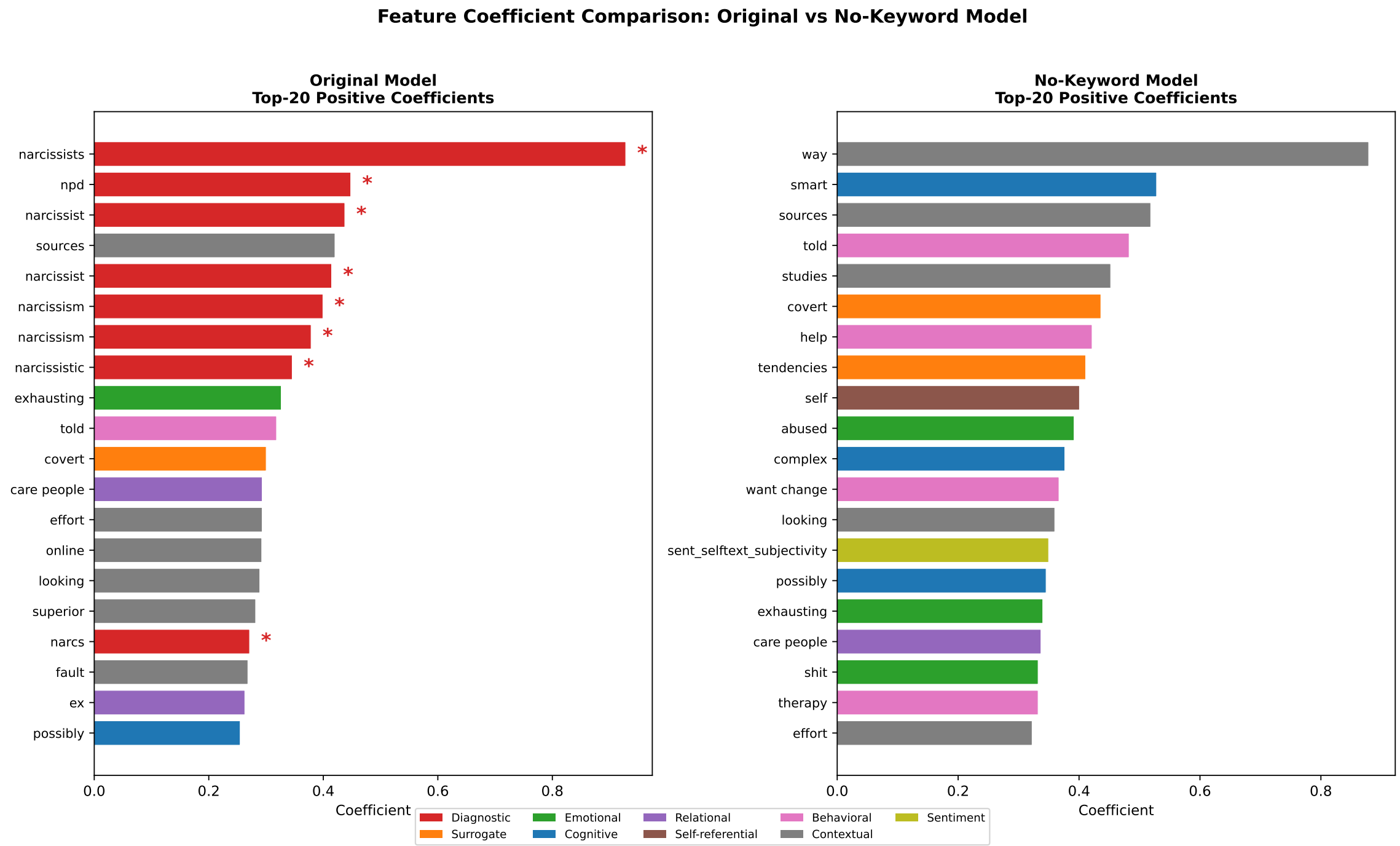

Figure 7: Top-20 positive coefficient comparison: original vs no-keyword model (A1)

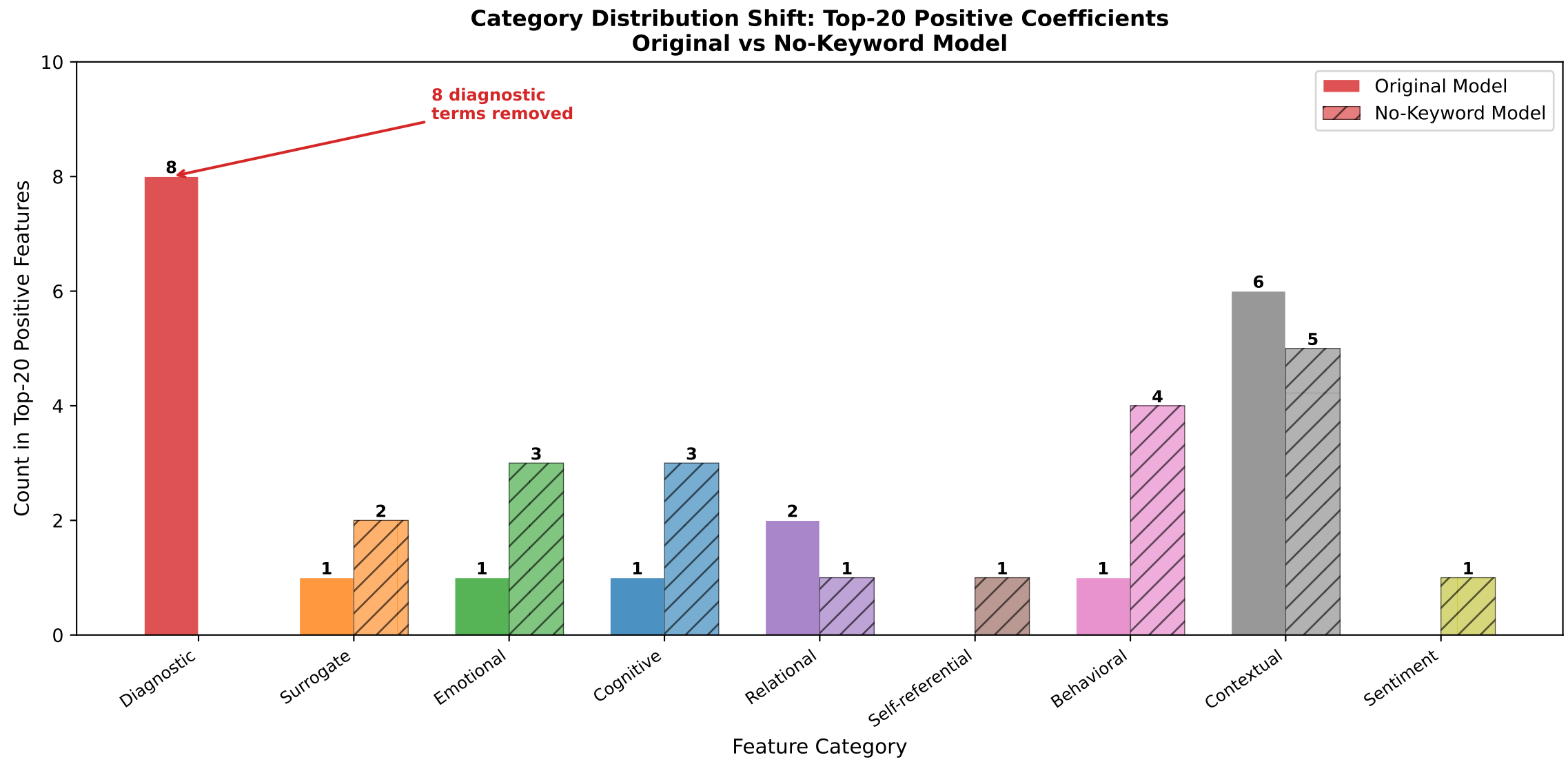

Figure 8: Category distribution shift in top-20 positive coefficients (A1)

5.4 LIME Anchor Cases

The following table shows LIME explanations for held-out test posts, comparing original and no-keyword model explanations. Each case was validated with 3 stability runs.

Post 363 (true=1, pred_nokey=1, prob=1.0000, STABLE)

> *Younger person here, and I bother people. I tell myself I'm smart, but the way I tex...*

- Original top-5: text (+0.1730), t (+0.1557), bother (+0.1492), narcissistic (+0.1332), don (+0.1294)
- No-keyword top-5: way (+0.1584), smart (+0.1338), t (+0.1201), personal (+0.0901), text (+0.0893)

Post 369 (true=1, pred_nokey=1, prob=1.0000, STABLE)

> *I'd call myself an unofficial , the covert kind. I score extreme for authority and superiority...*

- Original top-5: authority (+0.1875), extreme (+0.1390), narcissist (+0.0849), covert (+0.0737), diagnosed (+0.0693)
- No-keyword top-5: extreme (+0.1856), authority (+0.1711), covert (+0.1502), average (-0.0438), 1 (+0.0406)

Post 310 (true=1, pred_nokey=1, prob=0.6325, STABLE)

> *For a couple of years now I've known I'm , and I've been trying to work on my...*

- Original top-5: behaviors (-0.1661), narcissist (+0.1465), false (+0.1299), build (+0.1259), self (+0.1101)

- No-keyword top-5: act (-0.2461), problem (+0.2348), self (+0.2333), build (+0.1633), false (+0.1626)

Post 1 (true=0, pred_nokey=1, prob=0.9108, STABLE – **misclassified: false positive**)

> *I've always seen myself as level-headed and open-minded. It would be...*

- Original top-5: normal (+0.1548), admit (+0.1507), Earth (-0.1249), considered (+0.1077), question (-0.0984)
- No-keyword top-5: way (+0.4027), normal (+0.1798), considered (+0.1737), admit (+0.1578), Earth (-0.1202)

Post 328 (true=1, pred_nokey=0, prob=0.1394, STABLE – **misclassified: false negative**)

> *Is my husband the , or is it me? He wanted me to choose between him and my young child, and I refused...*

- Original top-5: level (-0.1768), different (-0.1573), says (-0.1468), husband (+0.1322), anymore (+0.1223)
- No-keyword top-5: says (-0.2050), way (+0.1975), level (-0.1898), different (-0.1677), husband (+0.1609)

1 of 6 anchor cases was flagged as unstable and excluded from core interpretation.

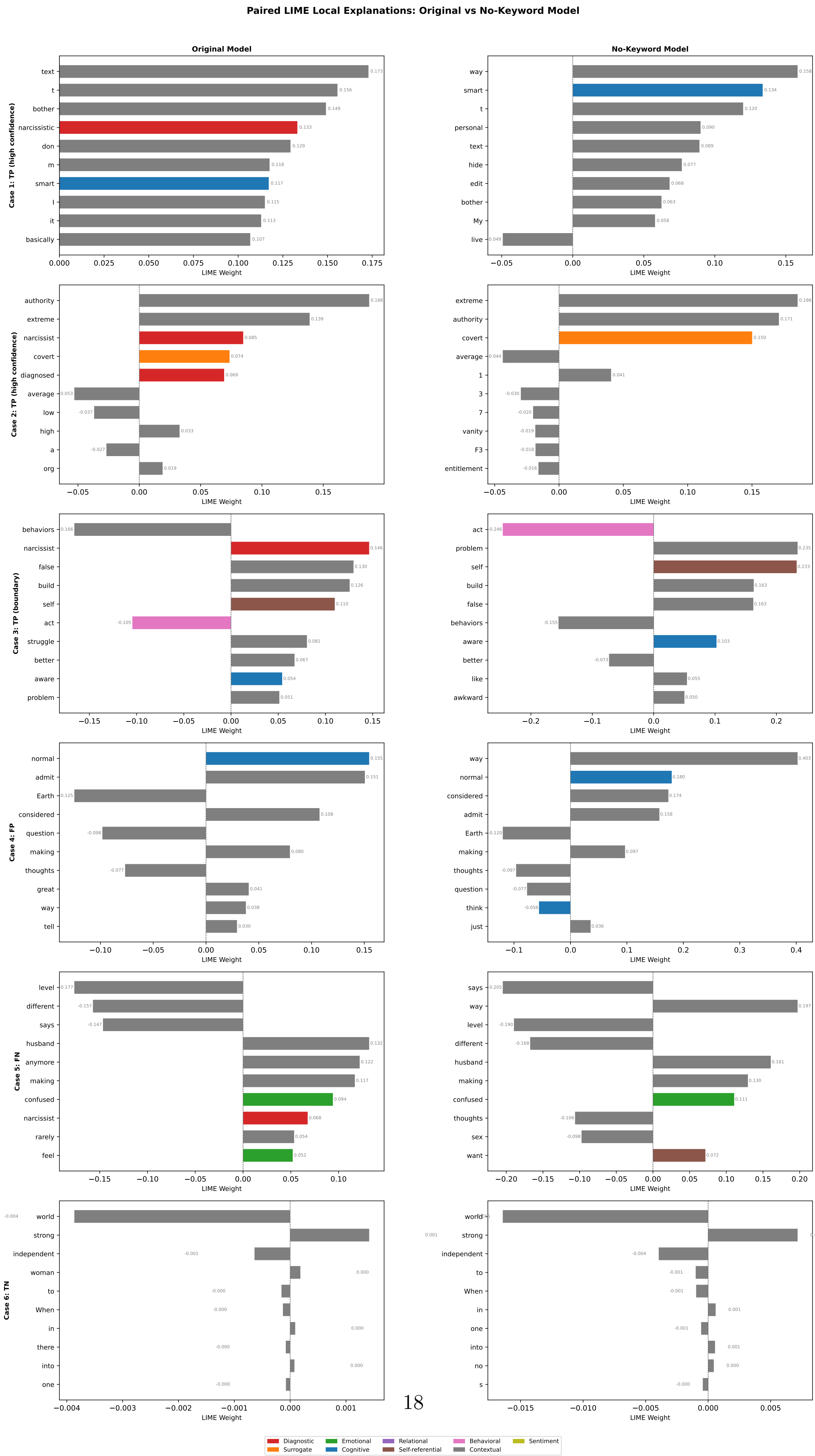

Figure 9: Paired LIME local explanations: original vs no-keyword model (A1)

5.5 Aggregate LIME Analysis

Aggregate LIME analysis across 74 no-keyword test posts (340 total top-5 feature slots):

Category distribution:

Category	Count	Fraction
Lexical surrogate / near-synonym	7	2.1%
Emotional / affective cue	17	5.0%
Cognitive / metacognitive	15	4.4%
Relational / interpersonal	15	4.4%
Self-referential / self-focus	8	2.4%
Behavioral / action-oriented	9	2.6%
Contextual / topical	269	79.1%

Contextual/topical features account for 79.1% of all LIME top-5 slots (see Section 7 for discussion). Of the contextual/topical slots, 170 (50.0% of all slots) were assigned by default (not in the pre-specified coding guide).

Top-10 most frequent features in LIME top-5 (no-keyword model):

Rank	Feature	Count
1	way	26
2	like	23
3	just	12
4	people	9
5	stop	6
6	normal	6
7	making	5
8	personal	5
9	issues	5
10	talk	5

Note: The dominance of 'way' (26 appearances) and other high-frequency function words in the A1 LIME top-5 illustrates the structural limitation discussed in Section 7. These features are linguistically vacuous and their prominence reflects LIME's sensitivity to high-TF-IDF-weight tokens rather than meaningful model behavior. The coefficient analysis (Section 5.3) provides a more reliable view of A1's learned features.

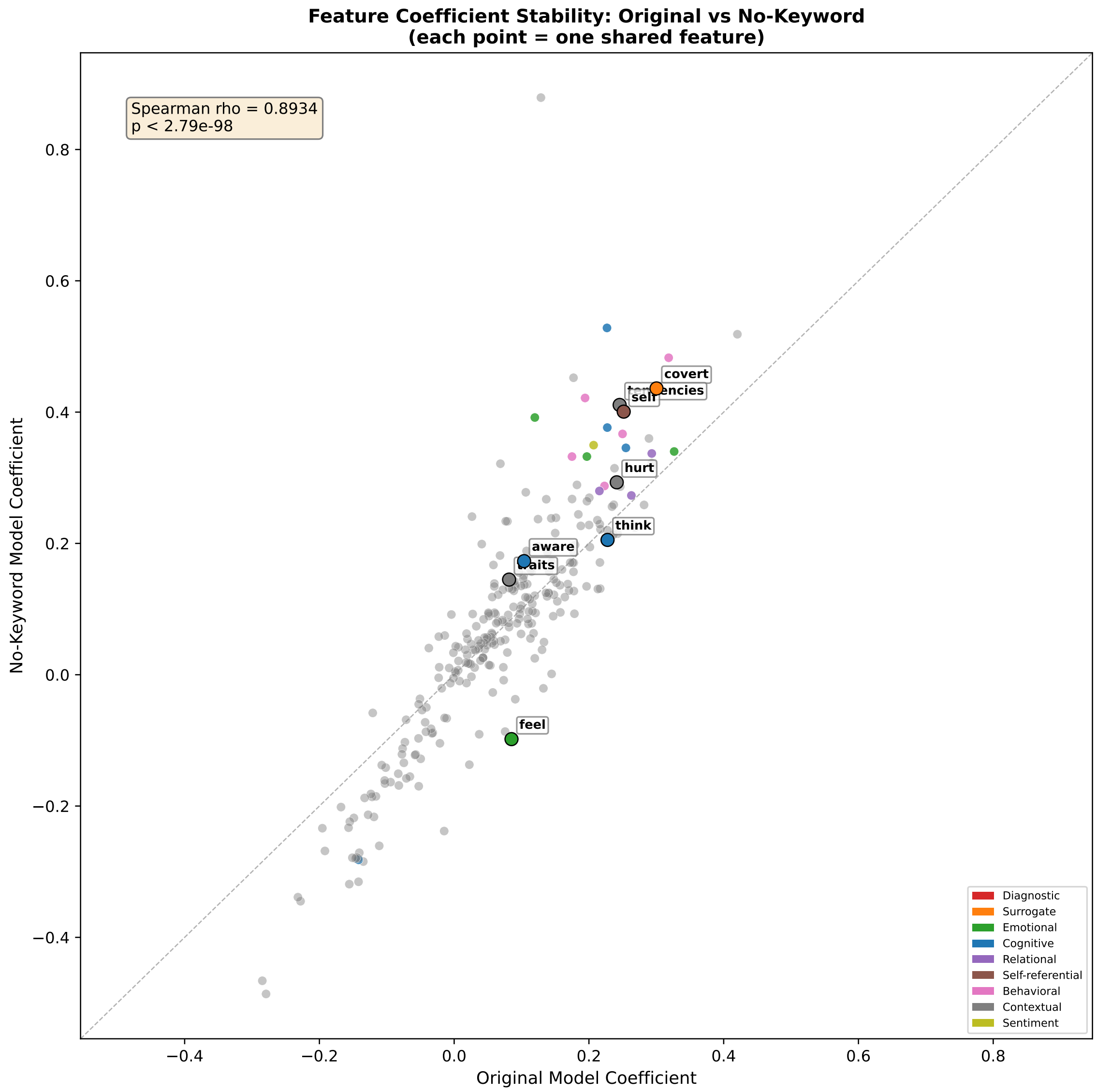

Figure 10: Feature coefficient scatter plot: original vs no-keyword (A1)

6. Hard Test: A5 (Narcissism+ vs Narcissism-)

6.1 Why A5 is the Hardest Test

A5 contrasts two subgroups from the same subreddit (r/NPD): users with diagnosed NPD (Narcissism+) and users without a diagnosis (Narcissism-). This is the hardest classification task because:

- Both classes share the same community vocabulary and topical context.
- Explicit diagnostic keywords appear in both classes (though at different rates).
- The baseline F1 is already moderate, reflecting genuine difficulty in distinguishing these subgroups.

6.2 Keyword Audit

Family	Pre-removal Count	Post-removal Count	Status
narcissism	1076	0	Verified
npd	157	0	Verified
disorder	82	0	Verified
personality	100	0	Verified
diagnose	64	0	Verified
self_absorbed	9	0	Verified

6.3 Three-Model Ablation

Test set size: N = 74.

Model	F1	Accuracy	Precision	Recall
Original	0.667	0.676	0.686	0.649
No-keyword	0.620	0.635	0.647	0.595
No-surrogate	0.630	0.635	0.639	0.622

Bootstrap 95% CIs and McNemar tests:

Comparison	F1 Delta 95% CI	McNemar p	N discordant
Original vs No-keyword	[-0.043, 0.144]	0.453	7
No-keyword vs No-surrogate	[-0.080, 0.055]	1.000	4

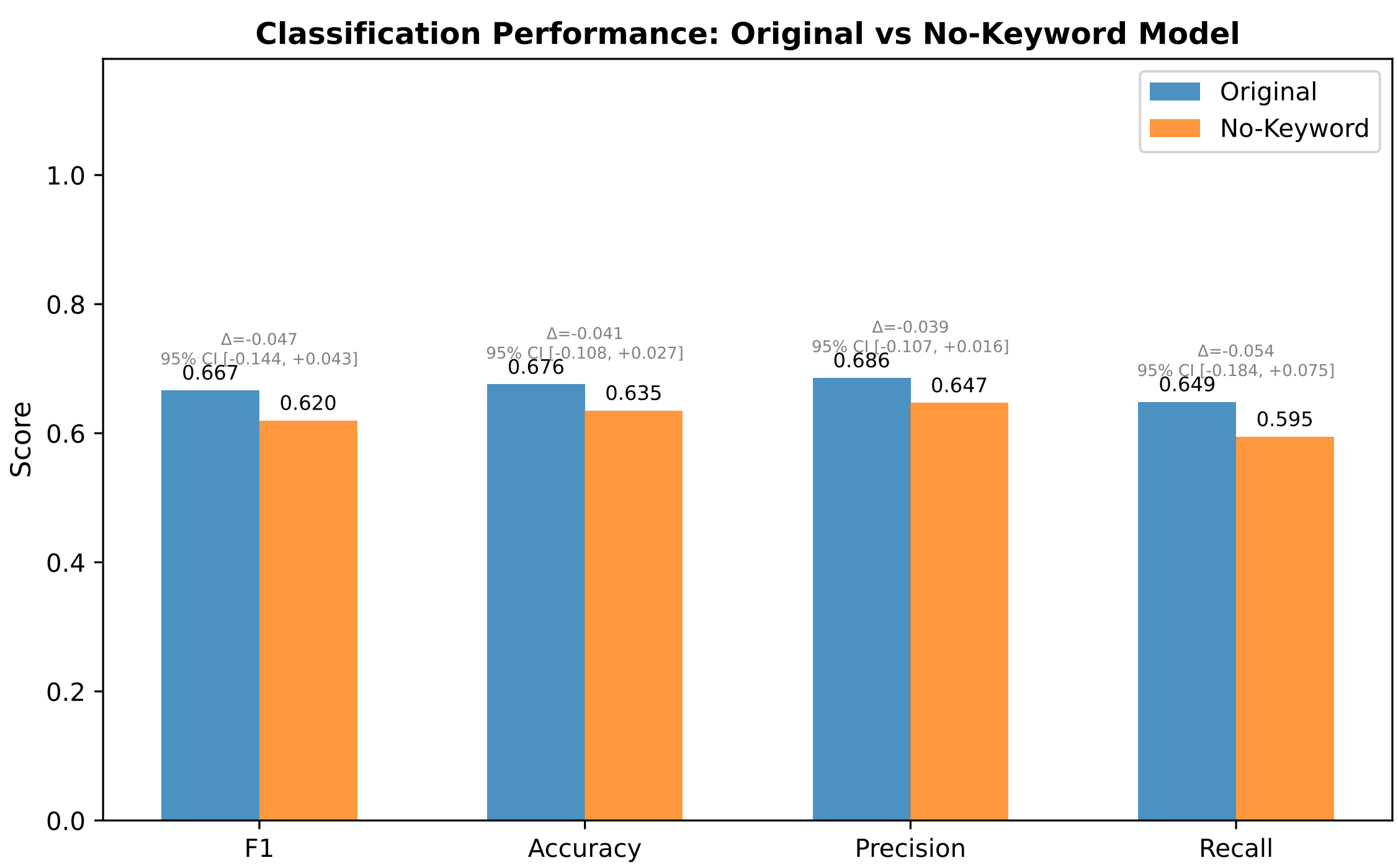

Figure 11: Classification performance: original vs no-keyword model (A5)

6.4 Key Finding

The point estimate of the keyword-removal F1 drop is 7.0% (from 0.667 to 0.620), but the bootstrap 95% CI for the original vs no-keyword delta spans zero ([-0.043, 0.144]), meaning this drop is not statistically distinguishable from no change at this sample size. The bootstrap 95% CI for the no-keyword vs no-surrogate F1 delta also spans zero ([-0.080, 0.055], with only 4 discordant pairs), meaning the small F1 increase from 0.620 to 0.630 after surrogate removal is within sampling noise and should not be interpreted as a meaningful difference. (The direction reversal – removing more features slightly improving F1 – can occur when a noisy or collinear feature is dropped from a regularized model, and is consistent with sampling variance at this sample size.)

With only 74 test posts, the A5 keyword-removal drop is too uncertain to interpret on its own: its bootstrap 95% confidence interval spans zero, so it is not distinguishable from no change. The random-removal baseline (Section 6.4.1) agrees — removing the diagnostic keywords degrades A5 no more than removing an equal number of arbitrary features. A firm single-experiment conclusion is therefore precluded; the cross-experiment pattern (Section 3), where no experiment collapses entirely after keyword removal, provides stronger aggregate evidence.

6.4.1 Random Feature-Removal Baseline

To contextualize the keyword-removal F1 drop, 1000 iterations of random feature zeroing (12 features per iteration, matching the keyword feature count, from the 300-feature post-selection space) were compared against keyword zeroing on the trained original model (zeroing keyword features without retraining — distinct from the retrained no-keyword model's 7.0% point-estimate drop in Section 6.3). The keyword-removal F1 drop (0.000) was larger than 36% of random-removal drops (mean random drop: 0.002, 95% range of random drops: [-0.036, +0.038]). Removing the diagnostic keywords thus degrades performance no more than removing a typical random feature subset of equivalent size in this within-community setting — an absence of a detectable difference, not positive proof of zero signal.

6.5 Coefficient Analysis

Original model — top-20 positive coefficients:

Rank	Feature	Coefficient
1	anymore	0.6875
2	aware	0.5865
3	second	0.5704
4	think	0.5427
5	thanks	0.4904
6	look	0.4857
7	low	0.4645
8	afraid	0.4551
9	problem	0.4404
10	actually	0.4330
11	hurt	0.4161
12	terrible	0.4018
13	edit	0.4005
14	learning	0.3956
15	wife	0.3944
16	sources	0.3899
17	covert	0.3750
18	narcs	0.3728
19	seeing	0.3723
20	sure	0.3629

The original A5 model has only 1 explicit diagnostic term in the top-20 – far fewer than A1 or A2. This reflects the shared-vocabulary nature of the within-subreddit comparison.

No-keyword model — top-20 positive coefficients:

Rank	Feature	Coefficient
1	low	0.6274
2	think	0.6216
3	look	0.5453
4	problem	0.5239
5	aware	0.5130
6	covert	0.5111
7	thanks	0.5035
8	second	0.5011
9	anymore	0.4982
10	hurt	0.4563
11	actually	0.4347
12	learning	0.4200
13	sure	0.4195
14	assume	0.4124
15	terrible	0.4001
16	afraid	0.3998
17	wife	0.3932
18	anybody	0.3863
19	hate	0.3782
20	especially	0.3590

Category distribution shift (top-20 positive features):

Category	Original Count	No-Keyword Count	Delta
Explicit diagnostic term	1	0	-1
Lexical surrogate / near-synonym	1	1	+0
Emotional / affective cue	3	4	+1
Cognitive / metacognitive	4	5	+1
Relational / interpersonal	1	2	+1
Self-referential / self-focus	0	0	+0
Behavioral / action-oriented	1	1	+0
Contextual / topical	9	7	-2
Sentiment score (TextBlob)	0	0	+0

Observed shift: Explicit diagnostic terms: 1 -> 0 in top-20 positive. Broader cues (emotional + cognitive + relational + self-referential + behavioral) collectively: 9 -> 12. This redistribution is largely mechanical: removing 1 diagnostic term from a fixed top-20 necessarily promotes the next-ranked features, which are non-diagnostic by construction; the coefficient analysis above is more informative than the category count shift.

Residual surrogate note: 'covert' appears at rank 6 in the A5 no-keyword model (rank 6 in A1; see Section 5.3 for why it functions as a residual lexical surrogate). The no-surrogate ablation step removes this term, and the negligible F1 change confirms that the model does not depend on it.

Boundary-case note: 'mental illness' (no-keyword model, rank 22) is coded as contextual/topical in the feature guide, but in the NPD subreddit context it functions as a quasi-diagnostic reference. Readers may reasonably classify it as a lexical surrogate; this ambiguity is one instance of the coding boundary decisions discussed in Section 7.

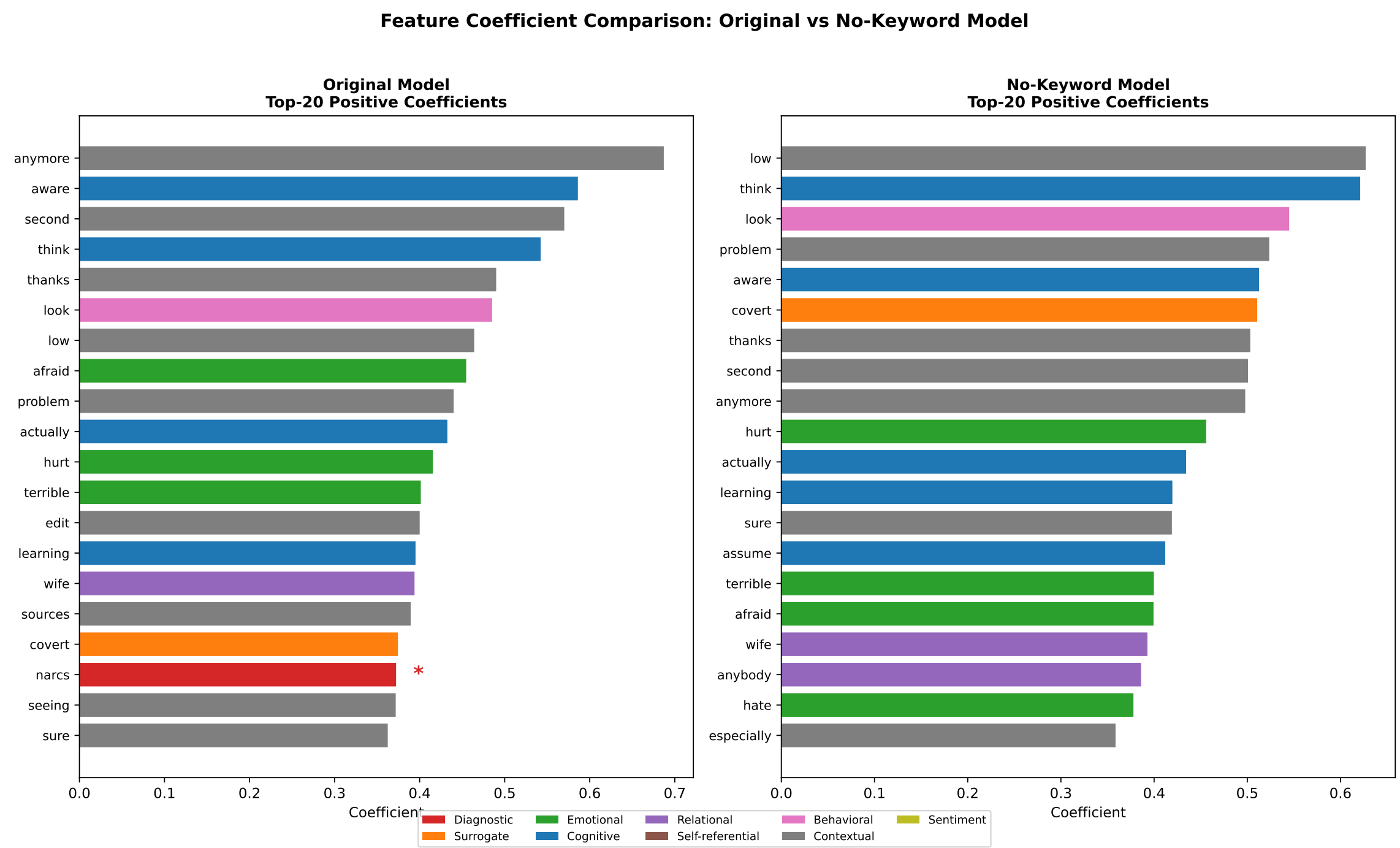

Figure 12: Top-20 positive coefficient comparison: original vs no-keyword model (A5)

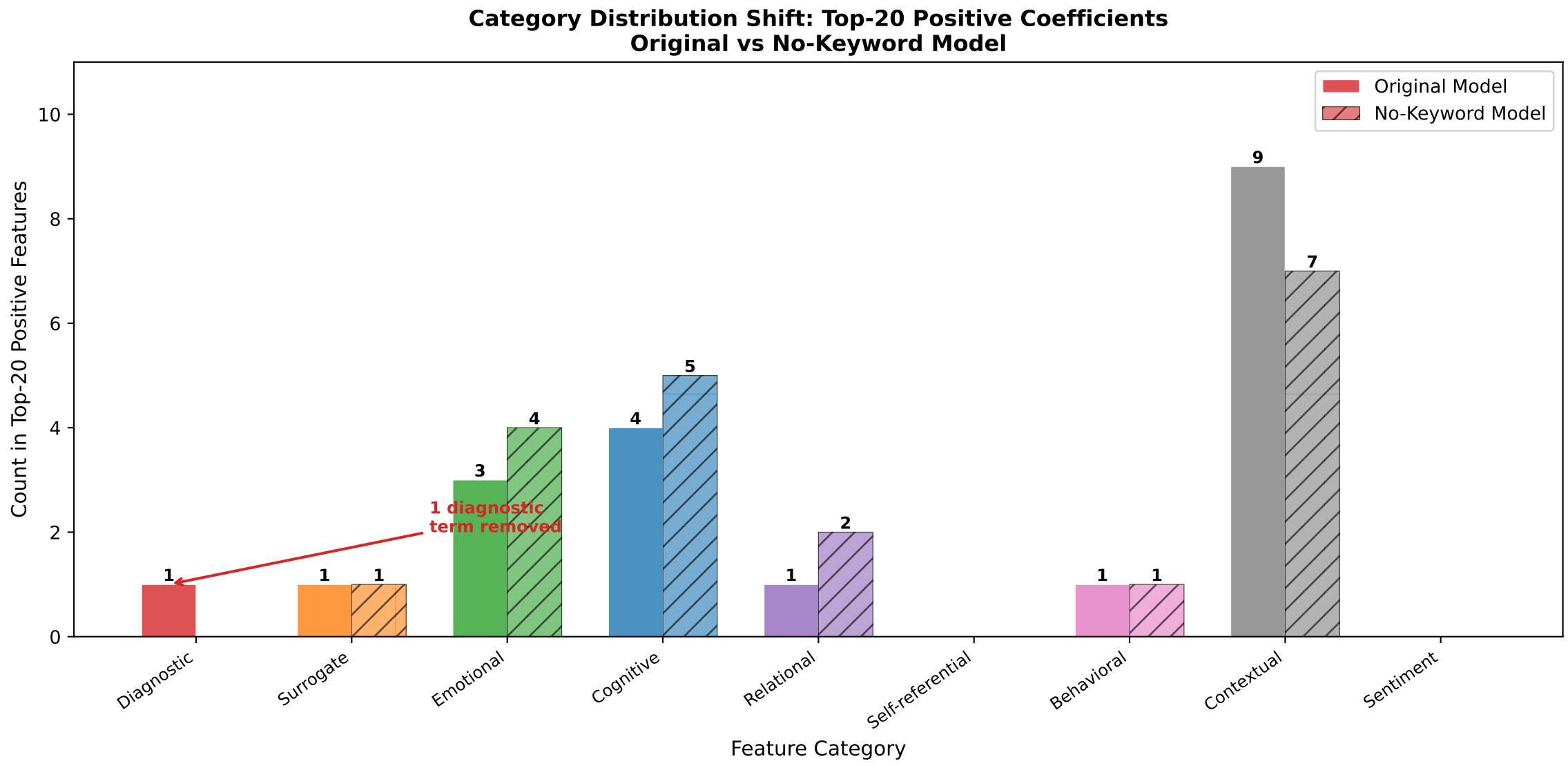

Figure 13: Category distribution shift in top-20 positive coefficients (A5)

6.6 LIME Anchor Cases

The following table shows LIME explanations for held-out test posts, comparing original and no-keyword model explanations. Each case was validated with 3 stability runs.

Post 369 (true=1, pred_nokey=1, prob=1.0000, STABLE)

> *I'd call myself an unofficial , the covert kind. I score extreme for authority and superiority...*

- Original top-5: authority (+0.1142), diagnosed (+0.1037), low (+0.1017), covert (+0.0811), F1 (-0.0367)
- No-keyword top-5: authority (+0.1693), low (+0.1665), covert (+0.1379), 3 (-0.0271), one (-0.0235)

Post 238 (true=1, pred_nokey=1, prob=1.0000, STABLE)

> *Please just hear me out. I'm close to tears and have been struggling so much...*

- Original top-5: enjoy (+0.0407), afraid (+0.0324), needed (+0.0179), though (+0.0171), silly (+0.0156)
- No-keyword top-5: enjoy (+0.0500), afraid (+0.0471), grew (+0.0437), awkward (+0.0220), tears (+0.0129)

Post 329 (true=1, pred_nokey=1, prob=0.5627, STABLE)

> *Even if I'll always carry that grandiose sense of being special (which I'll admit...*

- Original top-5: sure (+0.2350), special (-0.2024), great (+0.1848), empathy (-0.1679), better (+0.1493)

- No-keyword top-5: sure (+0.3125), great (+0.2448), better (+0.1816), special (-0.1403), relationships (+0.1026)

Post 54 (true=0, pred_nokey=1, prob=0.9883, STABLE – **misclassified: false positive**)

> ***You can't help a person who refuses to see they have a problem.*** *Picture...*

- Original top-5: ego (-0.2989), aware (+0.2763), deeply (+0.2362), problem (+0.2083), tend (+0.2080)
- No-keyword top-5: tend (+0.2626), deeply (+0.2099), ego (-0.1778), problem (+0.1291), aware (+0.1019)

2 of 6 anchor cases were flagged as unstable and excluded from core interpretation.

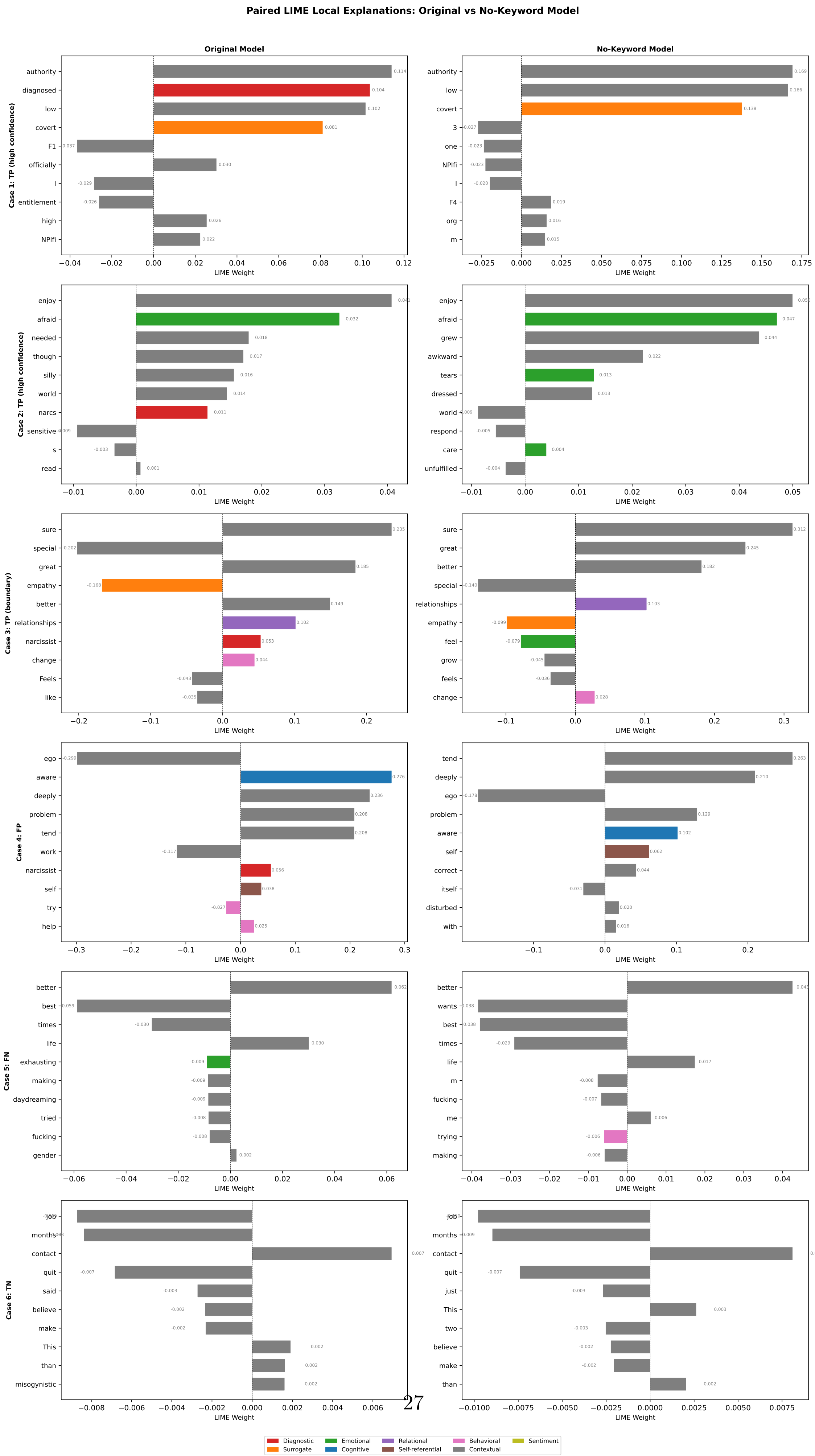

Figure 14: Paired LIME local explanations: original vs no-keyword model (A5).

6.7 Aggregate LIME Analysis

Aggregate LIME analysis across 74 no-keyword test posts (331 total top-5 feature slots):

Category distribution:

Category	Count	Fraction
Lexical surrogate / near-synonym	7	2.1%
Emotional / affective cue	24	7.3%
Cognitive / metacognitive	23	6.9%
Relational / interpersonal	14	4.2%
Self-referential / self-focus	10	3.0%
Behavioral / action-oriented	16	4.8%
Contextual / topical	237	71.6%

Contextual/topical features account for 71.6% of all LIME top-5 slots (see Section 7 for discussion). Of the contextual/topical slots, 157 (47.4% of all slots) were assigned by default (not in the pre-specified coding guide).

Top-10 most frequent features in LIME top-5 (no-keyword model):

Rank	Feature	Count
1	problem	9
2	thanks	9
3	sure	9
4	actually	8
5	look	8
6	better	7
7	life	7
8	want	7
9	aware	6
10	great	6

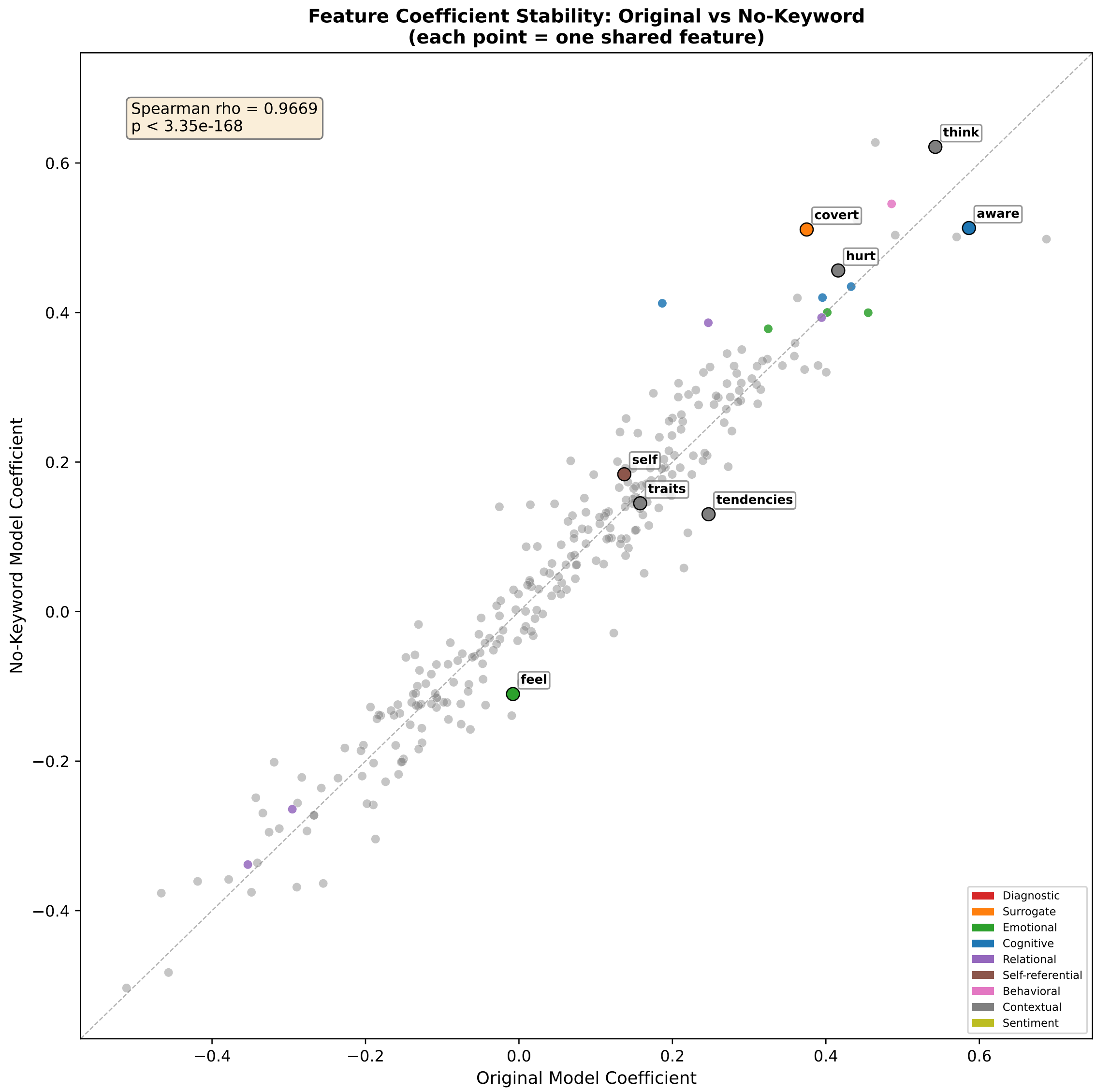

Figure 15: Feature coefficient scatter plot: original vs no-keyword (A5)

7. LIME Structural Limitation

Across all three deep-tier experiments, contextual/topical features dominate LIME's top-5 explanations:

Experiment	Contextual/Topical Fraction
A1	79.1%
A2	54.6%
A5	71.6%

The contextual/topical category accounts for 55%–79% of all LIME top-5 feature slots across experiments. This is a structural characteristic of combining LIME with TF-IDF features, not a model-specific failure:

- **TF-IDF features are sparse**: most features have zero weight for any given post, so LIME perturbations primarily affect the few non-zero features present.

- **Contextual words dominate non-zero slots**: common words that appear in a given post (e.g., 'school', 'working', 'time') naturally fill the top explanation slots.
- **Category-informative features are rare per post**: theoretically meaningful features (emotional, relational, cognitive cues) may appear in only 1-2 of the top-5 slots for any individual post, even when they are globally important in the model's coefficient vector.

Implication: LIME was applied to provide local, instance-level explanations. However, the TF-IDF sparsity artifact means that LIME top-5 explanations should not be interpreted as showing that the model relies primarily on contextual words. The logistic regression coefficient analysis (Sections 4.4, 5.3, and 6.5) provides a more reliable measure of global feature importance, because it reflects the full model rather than individual-post perturbation sensitivity.

Feature coding limitation: The 9-category coding scheme was applied by a single coder without inter-rater reliability assessment. Several category assignments are inherently ambiguous (e.g., 'confused' as emotional vs. cognitive; 'superior' as contextual vs. grandiosity-related). The category distribution shifts reported in Sections 4.4, 5.3, and 6.5 are sensitive to these boundary decisions, particularly given that 55%–79% of features fall into the default contextual/topical category. Readers should interpret the category distributions as approximate rather than precise.

Default-coding bias: Because uncategorized features default to contextual/topical, the reported fractions for psychologically meaningful categories (emotional, cognitive, relational, behavioral) are floor estimates. Any feature assigned to contextual by default that a second coder would place in a substantive category would shift the distribution toward higher non-contextual fractions. The true proportion of psychologically relevant features in LIME explanations may therefore be higher than reported.

8. Embedding-Based Keyword Robustness Check

To test whether the keyword-removal effect observed in the FSA pipeline generalizes to contextual representations, we repeated the original vs. no-keyword comparison using sentence embeddings (all-MiniLM-L6-v2, 384 dimensions) with three classifiers (Logistic Regression, Linear SVM, Random Forest). The same train/test splits were used. The table below reports the mean F1 across the three classifiers for each condition, alongside the FSA pipeline results from Section 3 for comparison.

Experiment	N (test)	FSA Orig F1	FSA No-KW F1	FSA Delta	Emb Orig F1	Emb No-KW F1	Emb Delta
A1	74	0.853	0.725	-15.0%	0.986	0.934	-5.3%
A2	50	1.000	0.987	-1.3%	0.982	0.962	-2.1%
A3	74	0.889	0.783	-12.0%	0.986	0.977	-0.9%
A4	74	0.776	0.771	-0.6%	0.855	0.854	-0.1%
A5	74	0.667	0.620	-7.0%	0.791	0.836	+5.7%
B1	204	0.923	0.873	-5.5%	0.963	0.895	-7.0%
B2	115	0.975	0.954	-2.1%	0.982	0.971	-1.1%
B3	204	0.939	0.861	-8.2%	0.985	0.955	-3.1%
B4	204	0.700	0.697	-0.4%	0.784	0.767	-2.2%
B5	183	0.570	0.618	+8.4%	0.703	0.678	-3.5%
C2	193	0.803	0.799	-0.5%	0.898	0.886	-1.3%
C3	151	0.837	0.852	+1.8%	0.889	0.880	-1.1%
C4	121	0.634	0.610	-3.8%	0.711	0.695	-2.4%

Aggregate: FSA mean relative F1 drop = 3.6% (mean absolute drop = 0.032, absolute SD 0.049, relative SD 6.1%); Embedding mean relative F1 drop = 1.9% (mean absolute drop =

0.017, absolute SD 0.026, relative SD 3.0%). The embedding pipeline shows roughly 52% of the FSA keyword-removal effect — a descriptive point-estimate ratio, not a tested difference, since the 13 experiments are non-independent (the A-, B-, and C-series each share a common positive pool) and the two pipelines are aggregated differently (see the note below).

Key observations:

1. **Embeddings show a smaller mean relative drop under keyword removal** (1.9% for embeddings vs. 3.6% for FSA). This is consistent with contextual embeddings distributing keyword semantics across the full 384-dimensional vector rather than relying on sparse token matches, though the per-experiment delta distributions overlap and the difference is reported descriptively rather than as a tested contrast.
2. **Experiments with the largest FSA drops (A1, A3, B3 and A5) show generally reduced drops under embeddings**, consistent with the FSA pipeline's keyword dependence reflecting a property of the bag-of-words representation rather than the underlying classification signal.
3. **A few experiments show slightly higher F1 after keyword removal under embeddings.** These small positive deltas are within the per-experiment sampling noise documented for these test-set sizes (the per-experiment McNemar and bootstrap comparisons are underpowered) and do not align across pipelines — the experiments that improve under FSA are not the ones that improve under embeddings — so they should not be read as a systematic benefit of keyword removal.
4. **Experiments that stay keyword-sensitive under both pipelines (A1, B1 and B3)** carry genuine discriminative signal from keyword presence even at the contextual level.

Note: Embedding F1 values reported here are the mean across Logistic Regression, Linear SVM, and Random Forest classifiers, whereas the FSA figures are for the single FSA + Logistic Regression pipeline. Averaging three classifiers mechanically narrows the embeddings' cross-experiment dispersion, so the FSA-vs-embedding comparison is descriptive rather than a controlled, scale-matched contrast. Per-classifier breakdowns are available in the supplementary output files. These results support performance-robustness claims but do not constitute direct interpretability analysis of the embedding models.

Headline-model replication: Repeating the ablation on the headline embedding model (text-embedding-3-large, 3072 dimensions) across the same 13 experiments and three classifiers produced a mean relative F1 drop of 0.8% (SD 1.8%, range: 3.8% drop to 2.7% increase), confirming the pattern holds with tighter cross-experiment variance than the MiniLM check (relative SD 1.8% vs. 3.0% for MiniLM).

9. Calibrated Conclusion

The evidence across 13 experiments supports a nuanced interpretation of keyword dependence in the FSA + Logistic Regression pipeline:

Keywords contribute contextually; cross-experiment patterns suggest the model retains signal without them. The mean relative F1 drop after keyword removal is 3.6%, with substantial variation across experiments. In some cases (A2, A4, C2 and B4), keyword removal has negligible impact; in a few (C3 and B5) it slightly improves performance; in others (A1, A3, B3, A5, B1, C4 and B2) it reduces performance by 2.1–15.0%. No experiment shows complete collapse.

Cross-experiment evidence:

- A2 (showcase): The model separates two mental health communities with near-perfect accuracy even after keyword and surrogate removal, relying on features like 'mental', 'behavior', 'confused', and 'abused'. (Note: 'empathy' also ranks high but is coded as a lexical surrogate due to its centrality in NPD diagnostic criteria.)
- A1 (supporting): The larger F1 drop suggests partial keyword dependence, but the no-keyword model still achieves reasonable discrimination using features like 'told', 'abused', 'self', and 'exhausting'.
- A5 (hard test): Even in the most challenging within-community comparison, the keyword-removal drop is not statistically distinguishable from zero (its bootstrap CI spans zero) and is no larger than removing arbitrary features of equal size, and the no-surrogate model shows no significant further change.

Residual surrogate ('covert'): The term 'covert' — shorthand for 'covert narcissist' (Sections 5.3 and 6.5) — appears at rank 6 in A1 and rank 6 in A5 no-keyword models. The no-surrogate ablation step removes it, and the negligible F1 change in both experiments confirms the models do not depend on this residual signal.

Scope limitations:

- The keyword family 'self absorbed' was added to the removal list and confirmed absent from all no-keyword models; the results above demonstrate that the models retain signal without it.
- These interpretability claims apply only to the FSA + Logistic Regression pipeline. Embedding-based models were not directly interpreted, but Section 8 reports their keyword-removal robustness for comparison.
- LIME explanations are subject to the structural limitation documented in Section 7. Coefficient analysis is the more reliable global importance measure. In practice, LIME was less informative than anticipated: the structural limitation means that local LIME explanations are dominated by contextual words, and the coefficient analysis is the more reliable evidence for the non-keyword signal claim.
- The keyword family list, while expanded to include 'self absorbed', may not capture all quasi-diagnostic terms. The surrogate removal step covers three terms ('covert', 'tendencies', 'traits') that were selected because they appeared in the top-20 no-keyword coefficients across deep-tier experiments and function as shorthand for NPD-related concepts (e.g., 'covert narcissist', 'narcissistic tendencies'). This represents a lower bound; other NPD community jargon (e.g., 'supply', 'love bombing', 'gray rock') was not removed and may carry residual diagnostic signal.

Descriptive feature-profile observation: The no-keyword feature profiles across deep-tier experiments share a descriptive pattern: terms associated with distress, self-awareness, and interpersonal difficulty (e.g., 'afraid', 'hurt', 'aware', 'therapy', 'want change') consistently rank higher than terms associated with grandiosity or entitlement. This pattern is consistent with the known composition of r/NPD as a support-seeking community, but we note it as a descriptive observation rather than a clinical finding, as it may reflect community self-selection rather than a psychologically generalizable signal.

10. Reproducibility

Parameter	Value
Pipeline	FSA + Logistic Regression (sklearn)
Text features	TF-IDF (1,3)-grams on selftext + title (min_df=0.02, k=300)
Sentiment	TextBlob polarity + subjectivity (per field, 4 features)
Classifier	LogisticRegression (C=1.0, max_iter=1000, random_state=42)
Train/test split	Stratified 80/20, saved per experiment
Keyword families	6 (narcissism, npd, disorder, personality, self_absorbed, diagnose)
LIME samples	1,000 per explanation (5,000 retry if unstable)
LIME stability	3 seeds (42, 123, 456), overlap >= 3/5
Feature coding	9-category guide, single coder (see limitation note in Section 7; full category definitions and rationale are in the `CATEGORY_GUIDE` dictionary in `analysis/feature_coding.py`)
Deep-tier experiments	A1, A2, A5
Standard-tier experiments	A3, A4, B1-B5, C2-C4
Excluded	C1 (multiclass, 3 labels)
