# Supplementary material for: Detecting Narcissistic Personality Disorder Traits on Forums: Proof-of-Concept Study
Source: J Med Internet Res. 2026 Jul 29;28:e75799. doi: 10.2196/75799 (PMC13469838; doi:10.2196/75799)
Supplement: Supplementary file 2 [file jmir_v28i1e75799_app2.docx]

# Appendix 2 - Technical Details

## Feature Engineering

We followed two different approaches: (i) frequency and sentiment analysis (ii) *text embedding* methods. For each experiment, we generated a random sampled dataset and applied each method on the dataset and compared the results.

### Frequency and Sentiment Analysis (FSA)

The first step in our frequency analysis process involved pre-processing the text data. We used *nltk* and *scikit-learn* packages for this purpose. This was needed to use only the relevant features within the text. We processed both the title and body of the reddit posts separately.

We started with cleaning the text where we removed urls, numbers and non-alphabetical characters. Then we moved on normalizing contractions (using the python’s contractions package) which involved expanding contracted words such as “it’s” to “it is”. This process ensured uniformity in our data. We then converted the text into lower case. This step was needed to avoid duplications caused by case differences. With this process, “Parent” and “parent” are counted as the same. We then applied the WordNet lemmatizer within the preprocessing function. Because it is invoked on the full text string rather than on individual tokens, its practical effect on multi-word posts is limited, and inflected word forms are generally preserved as distinct tokens. Finally, we tokenized the text by breaking down the text into individual words (also called tokens); tokens consisting solely of punctuation characters were excluded.

Once the tokens were ready, we removed the standard English stop words provided by scikit-learn. We used the tf-idf method on both title and body of the post to create 1-grams, 2-grams and 3-grams. This method quantifies the importance of a word in a document relative to its frequency in a corpus of documents. We could transform our text data into numerical form that could be analyzed by Machine Learning algorithms. We filtered out tokens with a document frequency below 2% (min_df = 0.02). We also applied sentiment analysis on the posts using textblob library and created 4 columns representing polarity (P) and subjectivity (S) for title and selftext data: Ptitle, Pselftext, Stitle, Sselftext

### Text Embedding Models

As an alternative to the combination of frequency and sentiment analysis, we used text embedding methods to convert the text of each post into a numerical form that our machine learning models could understand. We tested methods such as BERT [[1]](https://www.zotero.org/google-docs/?ohuiSA), LLaMA3 [[2]](https://www.zotero.org/google-docs/?4Tnjm9), MiniLM [[3]](https://www.zotero.org/google-docs/?M3SwsH), and Nomic [[4]](https://www.zotero.org/google-docs/?szAahV). Specifically, we used models like BERT base model (uncased), LLaMA3, all-MiniLM-L6-v2, nomic-embed-text, text-embedding-ada-002, and text-embedding-3-large. We used the Python *transformers* package and *Ollama* software [[5]](https://www.zotero.org/google-docs/?ZHOfsq) for most of these models. For the text-embedding-ada-002 and text-embedding-3-large models, we used the *openai* package.

## Feature Selection

Our feature selection process was a multi-step procedure designed to reduce noise and focus on the most predictive features. We aimed to enhance the performance of our Machine Learning model by reducing overfitting and training time and improving accuracy.

We first standardized each feature column with StandardScaler, which removes the mean and scales values to unit variance. We then removed zero-variance (constant) features with VarianceThreshold (threshold = 0), since constant features carry no discriminating information. Finally, we selected the most informative K features based on ANOVA F-values between the label and each feature. ANOVA is a statistical method that compares the means of more than two groups to determine whether there are statistically significant differences between them [6]; the higher the F-value, the more informative the feature. The optimal K for each experiment was chosen by Grid Search. This feature selection step (SelectKBest) was applied only to the frequency and sentiment analysis (FSA) features; for the transformer embedding models the corresponding pipeline slot is a passthrough, so no feature selection was applied to embeddings.

## Data Balancing

Real-world incidence of narcissistic traits in the general population is low (≈ 1 – 6 %) [11]. Consequently, when the Narcissism subreddit is contrasted with high-traffic controls (e.g., r/ShowerThoughts ≈ 72k posts) the raw corpus is necessarily imbalanced. This mirrors prospective surveillance settings in which the minority class is rare. We therefore adopted a two-step strategy: (i) the Setup A and B experiments except A2 and B2 were balanced at data preparation by equal sampling or undersampling, while (ii) Experiments A2 and B2 and the Setup C experiments (C1–C4) retained their natural class skew and relied on SMOTETomek (applied inside the training pipeline) to synthesise minority samples and remove Tomek links.

To avoid our models biasing towards the majority class in these experiments, we applied the SMOTETomek data balancing method. This method applies two methods consecutively: SMOTE [7] and Tomek Links [8]. SMOTE aims to generate synthetic samples by interpolating between existing minority class samples, increasing their representation in the dataset. Tomek Links remove ambiguous instances from the majority class that are close to the minority class boundary. Using both approaches within the SMOTETomek method improves class separation and reduces noise, making it particularly suitable for scenarios where class overlap is a concern. For experiments A5 and B5, we downsampled the larger (negative) class to balance the data, since SMOTETomek exacerbated the noise and performed poorly.

All held-out test folds remained *untouched*, so F1, ROC-AUC, and class-specific precision/recall are unbiased estimates for the test-set class distribution. Because that prevalence is far above the 1-6% population base rate, the reported precision and F1 overstate what deployment would show; prevalence-adjusted estimates are given in the main-text Limitations.

## Classification Algorithms

We used Logistic Regression (LR), Support Vector Machines (SVM), XG-Boost (XGB) and Random Forest (RF) classifiers in our study. LR has been known for its simplicity, efficiency and suitability for binary classification. Despite being a linear model, together with a nonlinear link function, it is a good choice for problems like text classification, where the decision boundary is not expected to be linear. Furthermore, LR provides probabilities for its predictions, which allows us to understand the confidence in its predictions. By adjusting the decision threshold, we can control the trade-off between predicting more true positives and reducing false positives. This is especially useful for mental health professionals who want to adjust the sensitivity when conducting surveillance on public platforms. However, in cases where LR failed to draw an accurate boundary, SVM, XGB and RF classifiers outperformed LR.

## Validation and Testing

For each experiment, we split the data into 80% for the train, 20% for the test and used the same split distribution for all feature models (FSA and embeddings).

We then applied the Grid Search method across a set of parameters (see the Hyperparameter Grids table below) and applied 5-fold cross validation to prevent overfitting and increase the model’s ability to generalize to unseen data. Cross validation involves splitting the train dataset into five folds, training with four folds and validating with the fifth one. This process was repeated five times, each with another fold as the validation set. We then calculated the average of each metric for each validation set. Comparing the validation performance of runs with different parameters, we found the best performing parameter set for each experiment - feature model combination. We then used these parameters to train the model with the whole train data (80%) and tested on the unseen test set (20%).

[[1]	J. Devlin, M.-W. Chang, K. Lee, and K. Toutanova, “BERT: Pre-training of Deep Bidirectional Transformers for Language Understanding,” May 24, 2019, *arXiv*: arXiv:1810.04805. doi: 10.48550/arXiv.1810.04805.](https://www.zotero.org/google-docs/?wb7w7E)

[[2]	H. Touvron *et al.*, “LLaMA: Open and Efficient Foundation Language Models,” Feb. 27, 2023, *arXiv*: arXiv:2302.13971. doi: 10.48550/arXiv.2302.13971.](https://www.zotero.org/google-docs/?wb7w7E)

[[3]	W. Wang, F. Wei, L. Dong, H. Bao, N. Yang, and M. Zhou, “MiniLM: Deep Self-Attention Distillation for Task-Agnostic Compression of Pre-Trained Transformers,” Apr. 06, 2020, *arXiv*: arXiv:2002.10957. doi: 10.48550/arXiv.2002.10957.](https://www.zotero.org/google-docs/?wb7w7E)

[[4]	Z. Nussbaum, J. X. Morris, B. Duderstadt, and A. Mulyar, “Nomic Embed: Training a Reproducible Long Context Text Embedder,” Feb. 03, 2025, *arXiv*: arXiv:2402.01613. doi: 10.48550/arXiv.2402.01613.](https://www.zotero.org/google-docs/?wb7w7E)

[[5]	*Ollama*. [Online]. Available: https://ollama.com/](https://www.zotero.org/google-docs/?wb7w7E)

[[6]	A. W. F. Edwards, “R.A. Fischer, statistical methods for research workers, first edition (1925),” in *Landmark Writings in Western Mathematics 1640-1940*, Elsevier, 2005, pp. 856–870. doi: 10.1016/B978-044450871-3/50148-0.](https://www.zotero.org/google-docs/?wb7w7E)

[[7]	N. V. Chawla, K. W. Bowyer, L. O. Hall, and W. P. Kegelmeyer, “SMOTE: Synthetic Minority Over-sampling Technique,” *J. Artif. Intell. Res.*, vol. 16, pp. 321–357, Jun. 2002, doi: 10.1613/jair.953.](https://www.zotero.org/google-docs/?wb7w7E)

[[8]	“Two Modifications of CNN,” *IEEE Trans. Syst. Man Cybern.*, vol. SMC-6, no. 11, pp. 769–772, Nov. 1976, doi: 10.1109/TSMC.1976.4309452.](https://www.zotero.org/google-docs/?wb7w7E)

## Technical Reproducibility

### 2.1 Environment

**Operating System and Hardware:**

- Operating System: cross-platform (developed on macOS; CI on Linux)
- Python Environment: the reported results were generated with the repository's pinned uv environment (pyproject.toml + uv.lock). Transformer embeddings were computed once in an earlier environment (pins below) and are cached (Parquet single source of truth); model training and all reported metrics read the cached embeddings.

**Python Version:**

- Python 3.13 (training environment, per the repository's requires-python >= 3.13; the cached embeddings were originally computed under Python 3.11 with torch 2.1.1)

Version-sensitive dependencies of the training environment (recorded in uv.lock; these determine the trained models and the reported metrics):

scikit-learn==1.7.2

imbalanced-learn==0.14.0

numpy==2.3.4

scipy==1.16.3

pandas==2.3.3

nltk==3.9.2

textblob==0.19.0

contractions==0.1.73

Version-sensitive dependencies of the embedding-generation environment (historical; these determine the cached embeddings):

sentence-transformers==2.2.2

transformers==4.35.0

tokenizers==0.15.0

torch==2.1.1

huggingface-hub==0.19.4

openai==1.23.6

**Global Random Seed:**

- Train/test split: random_state=42
- SMOTE-Tomek sampling: random_state=42
- Cross-validation: random_state=42

### 2.2 Data Acquisition

**Data Source:**

- Reddit API data stored in CSV files
- Experiment files: data/processed/experiments/experiment_{exp}.csv

**Data Loading Configuration:**

```
# From config.py
POSITIVE_LABELS = [7,8,9]  # Annotation labels for positive narcissism
EXPERIMENTS_DIR = PROCESSED_DIR / "experiments"

# From data/loader.py - experiment data reading
def read_experiment_data(experiment: str):
   df = pd.read_csv(EXPERIMENTS_DIR / f"experiment_{experiment}.csv")
   y = df["label"]
   X = df[["title", "selftext"]]
   return X, y
```

**Data Filtering:**

```
# From data/generate.py (applied when generating the experiment CSVs)
df = df[~df.selftext.isnull()]
df = df[df.selftext.str.len() > 99]  # Posts longer than 99 characters
```

### 2.3 Text Preprocessing (FSA Pipeline Only)

**Text Cleaning Pipeline:**

The preprocessing pipeline performs the following steps in sequence:

1. **Markdown and URL Removal:**

```
# Remove markdown links and formatting
text = text.replace("\\'", "'")
text = text.replace("can't", "can not")
text = re.sub(r"(?:__|[*#])|\[(.*?)\]\(.*?\)", "", text)

# Remove URLs using regex pattern
text = re.sub(
   r"(http:\/\/www\.|https:\/\/www\.|http:\/\/|https:\/\/)?[a-z0-9]+([\-\.]{1}[a-z0-9]+)*\.[a-z]{2,5}(:[0-9]{1,5})?(\/.*)?",
   "", text
)
```

2. **Text Normalization:**

```
# Remove digits completely
text = "".join([i for i in text if not i.isdigit()])

# Fix common formatting issues
text = text.replace("i'm", "I'm")
text = text.replace("\n", " ")
text = text.replace(" 's", "'s")
```

3. **Contraction Expansion:**

```
import contractions
text = contractions.fix(text)  # Expands contractions like "don't" → "do not"
```

4. **Case Normalization and Lemmatization:**

from nltk.stem import WordNetLemmatizer

lemmatizer = WordNetLemmatizer()

text = text.lower().strip()

text = lemmatizer.lemmatize(text) # Applied to the full string; limited effect on multi-word posts

**Tokenization:**

```
import string
from nltk import word_tokenize

# NLTK word tokenization; tokens made solely of punctuation are excluded
def tokenize_without_punctuation(text):
    tokens = word_tokenize(text)
    return [t for t in tokens if not all(c in string.punctuation for c in t)]

TfidfVectorizer(
   preprocessor=preprocess,        # Custom preprocessing pipeline above
   tokenizer=tokenize_without_punctuation,  # word_tokenize + punctuation-only token filtering
   token_pattern=None              # Use custom tokenizer instead of regex
)
```

**Stop Words Configuration:**

```
# Enhanced stop words list
from sklearn.feature_extraction.text import ENGLISH_STOP_WORDS
additional_stops = {'ha', 'le', 'u', 'wa'}
stop_words = list(set(ENGLISH_STOP_WORDS) | set(additional_stops))

# Keyword-omission ablation (--omit runs): these diagnostic keywords are
# not TF-IDF stop words: a KeywordRemovalTransformer strips them (including
# simple stem/variant forms) from the text before vectorization; embedding
# models use keyword-stripped (nokey) embedding variants instead.
EXCLUDED_KEYWORDS = [
   "narcissism", "narcissist", "narcissists", "npd",
   "narcisistic", "narcissistic", "disorder",
   "personality"
]
```

**Sentiment Analysis Implementation:**

```
import textblob

def get_sentiment_pair(post):
   """
   Extract sentiment features from text using TextBlob.

   Returns:
       tuple: (normalized_polarity, subjectivity)
   """
   # Create TextBlob object and extract sentiment
   sent = textblob.TextBlob(post).sentiment

   # Normalize polarity from [-1,1] to [0,1] range
   polarity = abs((sent.polarity + 1.0) / 2.0)

   # Ensure values are in valid range
   assert 0.0 <= polarity <= 1.0

   return (polarity, sent.subjectivity)

# Usage in SentimentTransformer
def estimate_sentiment(self, X: pd.Series) -> np.ndarray:
   result = np.array([get_sentiment_pair(post) for post in X])
   return result  # Returns array of (polarity, subjectivity) pairs
```

**Sentiment Feature Details:**

- **Polarity**: Sentiment orientation normalized to [0,1] where values closer to 1 indicate stronger sentiment (positive or negative)
- **Subjectivity**: Measure of opinion vs. fact in [0,1] range where 0 is objective and 1 is subjective
- Applied separately to both title and selftext fields
- Results in 4 sentiment features: title_polarity, title_subjectivity, selftext_polarity, selftext_subjectivity

### 2.4 Feature Engineering

#### Pipeline 1: FSA (Frequency and Sentiment Analysis)

**TF-IDF Parameters:**

```
TfidfVectorizer(
   preprocessor=preprocess,
   tokenizer=tokenize_without_punctuation,  # word_tokenize + punctuation-only token filtering
   token_pattern=None,
   ngram_range=(1, 3),          # Unigrams, bigrams, trigrams
   smooth_idf=True,
   min_df=0.02,                 # Min document frequency (fraction = 2%)
   stop_words=stop_words        # Enhanced stop words list
)
```

**Feature Components:**

- Title TF-IDF features: ngram_range=(1,3), min_df=0.02
- Selftext TF-IDF features: ngram_range=(1,3), min_df=0.02
- Sentiment features for both title and selftext using TextBlob

#### Pipeline 2: Embeddings

**Transformer Models:**

```
EMBEDDING_MODELS = [
   "fsa",                          # Frequency and Sentiment Analysis approach
   "text-embedding-3-large",       # OpenAI text-embedding-3-large
   "text-embedding-ada-002",       # OpenAI Ada-002
   "bert-base-uncased",            # BERT Base
   "llama3",                       # LLaMA-3
   "nomic-embed-text",             # Nomic Embed
   "all-minilm"                    # All-MiniLM-L6-v2
]
```

**Embedding Configuration:**

- Title embeddings: Separate embedding vectors for title text
- Selftext embeddings: Separate embedding vectors for selftext
- Feature concatenation: Title + selftext embeddings combined
- Dimension: 384 (all-MiniLM), 768 (BERT, nomic-embed-text), 1536 (text-embedding-ada-002), 3072 (text-embedding-3-large), 4096 (LLaMA3)

### 2.5 Model Training & Tuning

**Train/Test Split:**

```
# From data/loader.py - the 80/20 split is pre-computed, not recomputed here
# Fixed 80/20 stratified split (random_state=42) generated once into
# data/splits/experiment_splits.pkl, then reused for every model
train_idx, test_idx = get_experiment_split(experiment)
```

**Cross-Validation Configuration:**

```
# 5-fold stratified cross-validation applied on train data.
GridSearchCV(
   pipeline,
   param_grid,
   cv=StratifiedKFold(n_splits=5, shuffle=True, random_state=42),
   scoring='accuracy',      # CV model-selection metric (F1 is the reported test metric)
   n_jobs=min(8, cpu_count - 2),  # Bounded parallelism
   verbose=0
)
```

**Hyperparameter Grids:**

| **Model** | **Parameters** | **Values** |
| --- | --- | --- |
| **Logistic Regression** | C | [0.01, 0.1, 1.0, 10.0, 100.0] |
|  | penalty | ['l2'] |
|  | solver | ['liblinear'] |
| SVM (CalibratedLinearSVC) | C | [0.01, 0.1, 1.0, 10.0, 100.0] |
| Random Forest | n_estimators | [100, 300] |
|  | max_depth | [10, 20, None] |
|  | min_samples_split | [2, 5] |
| XGBoost | max_depth | [3, 6, 10] |
|  | learning_rate | [0.05, 0.1] |

For the multiclass experiment C1 (Logistic Regression within OneVsRestClassifier only), only the regularization strength C is tuned; the solver is scikit-learn's default lbfgs and the penalty is L2.

**Feature Selection:**

We used SelectKBest(f_classif) for feature selection on the FSA features only; for transformer embeddings this step is a passthrough. The number of selected features (kbest__k) was tuned jointly with the classifier over {100, 300, 500} ({100, 300} for XGBoost).

**Data Balancing:**

```
SMOTETomek(
   random_state=42,
   sampling_strategy='auto'    # Automatic balancing strategy
)
```

### 2.6 Validation & Testing

**Cross-Validation Metrics Aggregation:**

```
# Mean across 5 folds for model selection
best_score = grid_search.best_score_  # Mean CV score
```

**Final Evaluation on Test Set:**

```
# From evaluation/metrics.py evaluate_model
def evaluate_model(model, X_test, y_test, average="binary"):
   # Binary predictions at the fixed 0.5 decision threshold (no test-set tuning)
   y_pred = (y_pred_proba[:, 1] >= 0.5).astype(int)

   metrics = {
       'accuracy': accuracy_score(y_test, y_pred),
       'precision': precision_score(y_test, y_pred, average=average),
       'recall': recall_score(y_test, y_pred, average=average),
       'f1': f1_score(y_test, y_pred, average=average),
       'roc_auc': roc_auc_score(y_test, y_pred_proba[:, 1]),
   }
```

**Reported test-set metrics:**

- Binary classification: F1-score
- Multiclass (c1): F1-weighted
- Additional metrics: Accuracy, Precision, Recall, ROC-AUC
