# Supplementary material for: Detecting Narcissistic Personality Disorder Traits on Forums: Proof-of-Concept Study
Source: J Med Internet Res. 2026 Jul 29;28:e75799. doi: 10.2196/75799 (PMC13469838; doi:10.2196/75799)
Supplement: Supplementary file 3 [file jmir_v28i1e75799_app3.docx]

### Multimedia Appendix 6: Checklist for Adherence to Guidelines for Developing and Reporting Machine Learning Predictive Models in Biomedical Research

This checklist details our adherence to the key recommendations for reporting on the development and evaluation of machine learning predictive models.

| **Item/Topic** | **Reported** | **Location in Manuscript & Details** |
| --- | --- | --- |
| **Title & Abstract** |  |  |
| 1. Model Identification | Yes | **Title & Abstract:** The title ("Detecting NPD-Traits...") and abstract clearly identify the study's purpose as developing a classification model for a health-related outcome. |
| 2. Structured Summary | Yes | **Abstract:** The abstract is structured to provide a summary of the study's background, objective, methods, key results (mean F1 = 0.90), and conclusions regarding the model's potential and limitations. |
| **Introduction** |  |  |
| 3. Background & Rationale | Yes | **Introduction:** This section details the clinical significance of NPD, the challenges in diagnosis, and the rationale for using NLP on social media data to detect trait markers. |
| 4. Objectives | Yes | **Introduction / Aims, Objectives...:** The objective is clearly stated: "to develop and rigorously evaluate embedding-based classifiers for detecting narcissistic grandiosity in Reddit discourse." |
| **Methods** |  |  |
| 5. Study Design | Yes | **Methods / Experiments Design:** The study is a retrospective analysis of publicly available text data. The design of 14 distinct classification experiments (Setups A, B, C) is described in detail. |
| 6. Data Source | Yes | **Methods / Data Collection:** The data source is identified as the Google BigQuery Reddit archive (2008–2019), with specific subreddits (r/Narcissism, r/DeepThoughts, etc.) listed. |
| 7. Eligibility Criteria | Yes | **Methods / Subreddit selection and post filtering:** Inclusion criteria (English language, >100 characters) and exclusion criteria (empty or deleted posts) are explicitly stated. |
| 8. Outcome Definition | Yes | **Methods / Data Labelling & Experiments Design:** The outcome ("NPD-trait-positive") is clearly defined based on a 9-label schema developed with and annotated by two psychiatrists. The specific labels constituting a positive case are detailed for each experimental setup (e.g., L7, L8, L9 for Setup A). |
| 9. Predictor Variables | Yes | **Methods / Feature Engineering Summary & Machine Learning Pipeline Overview:** Predictors (features) are described in two main categories: (1) Frequency and Sentiment Analysis (FSA) using TF-IDF and sentiment scores, and (2) Transformer-based text embeddings (e.g., text-embedding-3-large, llama3). |
| 10. Data Preprocessing | Yes | **Methods / Class imbalance & resampling strategy:** Steps for handling class imbalance, a key preprocessing step, are detailed (random undersampling for noisy data, SMOTETomek for cleaner data). |
| 11. Model Development | Yes | **Methods / Machine Learning Pipeline Overview:** The model development process is fully described, including the algorithms used (Logistic Regression, SVM, Random Forest, XGBoost), the 80/20 train-test split, and the use of 5-fold cross-validation on the training set for hyperparameter tuning. |
| 12. Performance Evaluation | Yes | **Methods / Machine Learning Pipeline Overview & Results / Table 10:** Performance metrics are clearly defined (F1, ROC AUC, Accuracy, Precision, Recall) and reported on the held-out 20% test set. |
| **Results** |  |  |
| 13. Data Flow & Counts | Yes | **Results / Data Extraction Results & Table 8:** The number of posts extracted from each subreddit is reported, providing a clear account of the data used. |
| 14. Model Performance | Yes | **Results / Experiment Results & Table 10:** The performance of the best models for all 14 experiments is presented in a clear tabular format, allowing for easy comparison. |
| **Discussion** |  |  |
| 15. Limitations | Yes | **Discussion / Limitations:** A detailed discussion of limitations is provided, including dataset size, temporal scope (pre-2020 data), language limitations (English-only), and the potential for overfitting. |
| 16. Interpretation & Implications | Yes | **Discussion & Conclusion:** The results are interpreted in the context of prior work, and the clinical implications for mental health screening and surveillance are discussed, along with a strong emphasis on ethical considerations. |
| **Other** |  |  |
| 17. Code Availability | Yes | **Methods / Data Availability:** The manuscript states in the Data Availability section that "The code used for model training and evaluation is publicly available at <https://github.com/aladagemre/npd-traits>". |
| 18. Ethical Approval | Yes | **Methods / Ethical Considerations:** The study's ethical approval (Boğaziçi University IRB Exemption No. 2025-13) is explicitly stated. |
