# Supplementary material for: Detecting Narcissistic Personality Disorder Traits on Forums: Proof-of-Concept Study
Source: J Med Internet Res. 2026 Jul 29;28:e75799. doi: 10.2196/75799 (PMC13469838; doi:10.2196/75799)
Supplement: Supplementary file 4 [file jmir_v28i1e75799_app4.docx]

# Appendix 1

## **Experiment Design - Data Distribution**

Another visualization of the data distribution.

|  | **Narcisissm** | | | | | | | | | **DT** | **ST** | **IS** |
| --- | --- | --- | --- | --- | --- | --- | --- | --- | --- | --- | --- | --- |
| **EX** | **L1** | **L2** | **L3** | **L4** | **L5** | **L6** | **L7** | **L8** | **L9** |  |  |  |
| **A1** |  |  |  |  |  |  | + | + | + | - | - | - |
| **A2** |  |  |  |  |  |  | + | + | + | - | - | - |
| **A3** |  |  |  |  |  |  | + | + | + | - | - | - |
| **A4** |  |  |  |  |  |  | + | + | + | - | - | - |
| **A5** | - | - | - | - | - | - | + | + | + |  |  |  |
| **B1** |  | + |  | + |  | + | + | + | + | - | - | - |
| **B2** |  | + |  | + |  | + | + | + | + | - | - | - |
| **B3** |  | + |  | + |  | + | + | + | + | - | - | - |
| **B4** |  | + |  | + |  | + | + | + | + | - | - | - |
| **B5** | - | + | - | + | - | + | + | + | + |  |  |  |
| **C1** | IR | NR | NI | NI | NR | NI | NI | NI | NI |  |  |  |
| **C2** | IR | NR or NI | | | | | | | |  |  |  |
| **C3** |  | NR | NI | NI | NR | NI | NI | NI | NI |  |  |  |
| **C4** |  |  | NP | NP |  | NA | NA | NA | NA |  |  |  |

**Figure B1:** Experiments and the subreddits used together with labels. Abbreviation descriptions are listed below:

**EX:** Experiment ID,

**L1-L9:** Posts in Narcissism subreddit with labels L1-L9.

**DT:** Posts in DeepThoughts subreddit

**ST:** Posts in ShowerThoughts subreddit

**IS:** Posts in Imposter Syndrome subreddit

**IR:** Irrelevant to NPD or individuals with NPD

**NI:** Individual with NPD-trait

**NR:** NPD-related

**NA:** Author exhibiting NPD-traits

**NP:** Individual with NPD-trait (other than author)

**+:** posts are assumed NPD-trait-positive

**-:** posts are assumed NPD-trait-negative

**Orange:** posts are included in the experiment as positive

**Blue:** posts are included in the experiment as negative

## **Grid Search Parameters**

We initially explored a parameter space broader than the one reported below to obtain interim results. Following this, we narrowed it empirically to the best-scoring range, which is the final grid used for all reported experiments and listed here.

For Experiment C1 (multiclass; Logistic Regression within OneVsRestClassifier only):

- Logistic Regression
- Classifier C_values (regularization strength) in {0.01, 0.1, 1.0, 10.0, 100.0}
- Classifier Solver: lbfgs (scikit-learn default; fixed, not grid-searched)
- Classifier Penalty: L2
- KBest
- KBest Scoring Function = f_classif (ANOVA)
- KBest K in {100, 300, 500} (FSA pipeline only)

For the rest of the experiments:

- Logistic Regression
  - Classifier C_values (regularization strength) in {0.01, 0.1, 1.0, 10.0, 100.0}
  - Classifier Solver: liblinear
  - Classifier Penalty: L2
- KBest
  - KBest Scoring Function = f_classif (ANOVA)
  - KBest K in {100, 300, 500}, {100, 300} for XGBoost (FSA pipeline only)
